# Light, Heat, and Force‐Responsive Polyolefins

**DOI:** 10.1002/advs.202307568

**Published:** 2024-01-06

**Authors:** Weicheng Qu, Zhengxing Bi, Chen Zou, Changle Chen

**Affiliations:** ^1^ Key Laboratory of Precision and Intelligent Chemistry Department of Polymer Science and Engineering University of Science and Technology of China Hefei 230026 China

**Keywords:** olefin polymerization, photo‐thermochromic/mechanochromic polymers, shape‐memory materials, spiropyrans, stimuli‐responsive materials

## Abstract

Stimuli‐responsive polymers have found applications as shape‐memory materials, optical switches, and sensors, but the installation of these responsive properties in non‐polar and inert polyolefins is challenging. In this contribution, a series of spiropyran (SP)‐based comonomers are synthesized and copolymerized with ethylene or ethylene/cyclic monomers. In addition to great mechanical and surface properties, these functionalized polyolefins responded to light, heat, and force, which induced changes in the polymer structure to transmit color or mechanical signals. These interesting responsive properties are also installed in a series of commercial polyolefin materials through reactive extrusion, making the scalable production of these materials possible.

## Graphical abstract

A spiropyran (SP)‐based polyolefin is shown here, which can simultaneously achieve exciting functions such as mechanochromism, photochromism, thermochromism, shape memory, and so on. More importantly, these interesting responsive properties are also installed in a series of commercial polyolefin materials through reactive extrusion, making the scalable production of these materials possible.

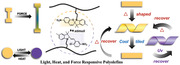

## 1 Introduction

**Scheme 1:**
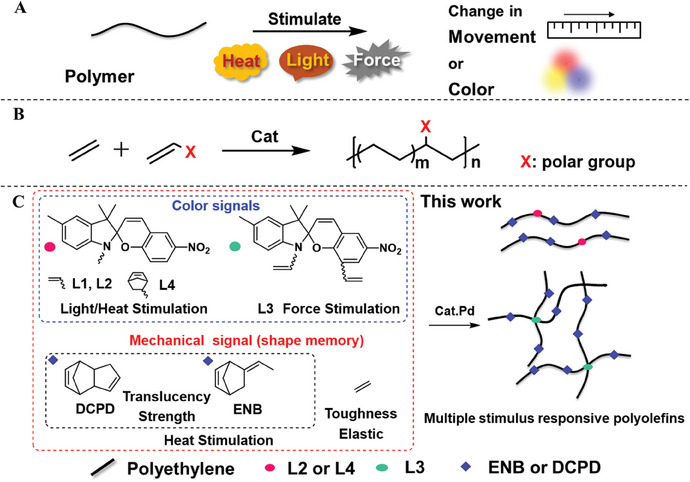
A) Schematic diagram of a polymer's response to stimuli. B) Transition metal‐catalyzed copolymerization of ethylene with polar comonomers. C) Preparation of photo‐thermochromic and mechanochromic polymers by the direct copolymerization of spiropyran and cyclic monomers with ethylene.

Stimuli‐responsive polymers have attracted widespread interest.^[^ ^1^, ^2^, ^3^, ^4^, ^5^ ^]^ For example, external stimuli such as light, heat, or electricity can alter a polymer's internal energy and lead to macroscopic movement or deformation,^[^ ^6^, ^7^, ^8^, ^9^, ^10^, ^11^, ^12^ ^]^ making these polymers useful as artificial muscles^[^ ^13^, ^14^ ^]^ or shape memory materials.^[^ ^15^, ^16^ ^]^ Selected polymers can also undergo light, heat, or mechanical force‐induced discoloration, giving them applications in the fields of optical rewritable data storage,^[^ ^17^ ^]^ optical switches,^[^ ^18^, ^19^ ^]^ force or strain sensing,^[^ ^20^ ^]^ and fluorescent probes^[^ ^21^ ^]^ (**Scheme** **1**A).^[^ ^22^, ^23^ ^]^ Generally, these stimuli‐responsive functional materials are prepared via the incorporation of specific stimuli‐responsive functional groups into polymers such as hydrogels,^[^ ^24^ ^]^ liquid crystal elastomers,^[^ ^25^ ^]^ or acrylics.^[^ ^26^ ^]^ In addition to a limited polymer scope, most studies have focused on polymers with only one response for color change^[^ ^27^ ^]^ or mechanical motion (such as shape memory).^[^ ^28^ ^]^ It is highly challenging to achieve multiple stimuli (light, heat, and force) and multiple responses (color change and mechanical motion) in a single polymer system.

In contrast to widely studied responsive polymers, the introduction of similar responsive functional groups into non‐polar polyolefins is more synthetically challenging. The construction of such responsive polyolefins could potentially realize wide‐scope and large‐scale applications, especially because of the huge annual production of polyolefins and their wide applications, low costs, and outstanding mechanical properties.^[^ ^29^ ^]^ However, their non‐polar nature presents a serious limitation.^[^ ^30^, ^31^ ^]^ Recently, transition metal‐catalyzed copolymerization of olefins with polar comonomers has provided an effective route for introducing various functional groups that can improve properties such as surface, adhesion, dyeability, and compatibility with other polymers (Scheme 1B).^[^ ^32^, ^33^, ^34^, ^35^ ^]^ Recent advances have afforded polyolefins with diverse functionalities, including outstanding mechanical properties,^[^ ^36^ ^]^ self‐healing,^[^ ^37^ ^]^ adhesion,^[^ ^38^ ^]^ degradability,^[^ ^39^ ^]^ antibacterial properties,^[^ ^40^, ^41^ ^]^ and dynamic crosslinking ability.^[^ ^42^ ^]^

Spiropyran (SP), is one of the most well‐studied stimuli‐responsive functional groups in all mechanoresponsive, photochromic materials.^[^ ^43^, ^44^ ^]^ When the force is transferred to the unstable spiral C‐O bond, or under the action of specific ultraviolet light, the colorless SP is activated to the color part merocyanine (MC). SP is a model mechanical and optical carrier in the basic research of polymer response behavior,^[^ ^45^ ^]^ which lays the foundation to realize implement multiple stimuli (light, heat, and force) and multiple responses (color change and mechanical motion) in a single polymer system. In this work, we demonstrate the synthesis of responsive polyolefins by the direct copolymerization of ethylene with SP‐based monomers or their copolymerization with cyclic monomers 5‐ethylidene‐2‐norbornene (ENB) and dicyclopentadiene (DCPD) using late transition metal catalysts (Scheme 1C). The introduction of cyclic monomer reduces the crystallinity and processing temperature of the material. The responsive polyolefins exhibited photochromism, thermochromism, and mechanochromism after the incorporation of different SP‐based monomers. By adjusting the content of SP‐based monomers and cyclic monomers, these polyolefin materials showed shape memory properties upon stimulation by light at room temperature or stimulation by heat. These functionalized polyolefin materials achieved three stimulus responses, light, force, and heat, to transmit mechanical or color signals. These functionalized polyolefin materials also showed a high molecular weight, tunable surface properties, and great mechanical properties.

## 2 Results and Discussion

### 2.1 Synthesis of Comonomers

Comonomer L1 was synthesized by the one‐step reaction of allyl‐substituted indole and benzaldehyde (**Figure** **1**). To obtain SP‐based monomers containing olefin substituents with different alkyl chain lengths (L2), double olefin units (L3), or cyclic norbornene structures (L4), pre‐formed hydroxyl functionalized SPs were esterified with acids.^[^ ^26^, ^46^ ^]^ Yields of more than 75% were achieved for all reactions. These comonomers were characterized by ^1^H NMR, ^13^C NMR, and ESI‐MS (Figures S1–S15, Supporting Information).

**Figure 1:**
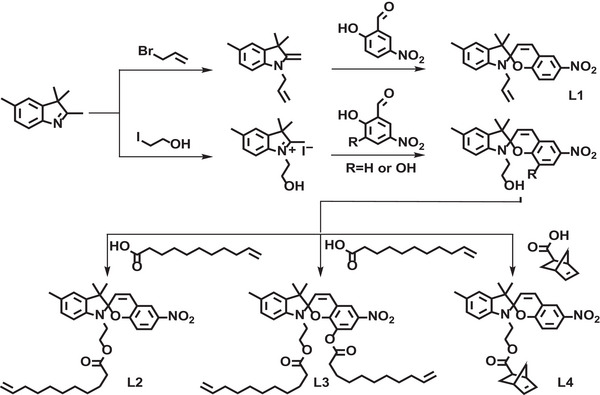
Synthesis of spiropyran‐based comonomers.

### 2.2 Synthesis of Responsive Polyolefins

In this work, a biaryl‐substituted phosphine‐sulfonate palladium (PO‐Pd) catalyst was selected because of its high tolerance toward polar functional groups and ability to generate high‐molecular‐weight copolymers.^[^ ^47^ ^]^ Comonomer L1 bearing an allyl group showed a low activity and low copolymer molecular weight because the SP group poisoned the catalyst (**Table** **1**, entry 1). In contrast, the long methylene spacer in comonomer L2 limited this poisoning (Table 1, entries 2 and 3), enabling high copolymer molecular weights (up to 3.19 × 10^5^ g mol^–1^) and high activities (up to 1.8 × 10^5^ g mol^−1^ h^−1^). To improve the elasticity of the polymer products, cyclic comonomers (ENB and DCPD) were introduced, whose incorporation ratios reached 15.4 mol%, while maintaining moderate SP comonomer (L2) incorporation (up to 0.7 mol%) and high copolymer molecular weights (up to 2.28 × 10^5^ g mol^−1^) (Table 1, entries 4 and 5). The force‐induced discoloration of the SP group requires that both sides of the spiral ring be connected to polymer segments. Therefore, comonomer L3 was deliberately designed and incorporated into a series of high‐molecular‐weight copolymers or terpolymers (Table 1, entries 6–8). To further increase the incorporation ratio of the SP comonomer and thus the transparency of the resulting polymers,^[^ ^48^ ^]^ a cyclic norbornene group was linked with SP (comonomer L4). Indeed, the incorporation rate of comonomer L4 reached 2 mol% (Table 1, entries 9–11). Finally, ethylene was copolymerized with comonomers L3, L4, and DCPD to prepare polyolefins with multiple stimuli‐responsive functional groups (Table 1, entry 12). These polar copolymers were prepared using transition metal catalysts, and the incorporation ratio of polar or cyclic monomers could be adjusted to obtain responsive polyolefin materials with different mechanical properties.

**Table 1:** Ethylene copolymerization studies with PO‐Pd.

Ent.^a)^	Comonomer 1	Comonomer 2	Yield/g	Act.^b)^/ 10^5^	X_mol_%^c)^	*T*_g_/*T*_m_ ^d)^ [°C]	*M*_n_ ^e)^/10^4^	PDI ^e)^
1	L1(0.5 M)	–	0.56	0.6	1.0	–/124.2	7.9	1.8
2	L2(0.5 M)	–	1.80	1.8	0.8	–/123.0	31.9	2.0
3	L2(1.0 M)	–	1.46	1.5	1.7	–/118.9	12.1	2.2
4	L2(0.5 M)	ENB	1.52	1.5	0.4/15.4	12.6/–	22.8	1.9
5	L2(0.5 M)	DCPD	1.60	1.6	0.7/6.9	−7.5/81.6	22.5	2.0
6^f)^	L3(0.5 M)	–	2.52	2.5	0.6	–/119.2	30.5	2.2
7^f)^	L3(0.5 M)	ENB	2.20	2.2	0.4/9.1	13.8/117.5	18.4	1.6
8^f)^	L3(0.5 M)	DCPD	2.35	2.4	0.8/5.0	–/89.0	13.1	2.3
9	L4(0.5 M)	–	1.38	1.4	2.0	–/118.8	26.2	1.8
10	L4(0.5 M)	ENB	1.64	1.6	1.2/15.4	24.6/–	16.6	1.6
11	L4(0.5 M)	DCPD	1.96	2.0	1.2/6.5	8.4/73.3	26.9	1.7
12^f)^	L3(0.25 M)/L4(0.25 M)	DCPD	2.05	2.1	0.6/0.6/5.6	0.5/63.1	21.4	1.7

^a)^ Conditions: PO‐Pd 10 µmol; 20 mL Tol; *P* = 8 atm; *t* = 1 h; *T* = 80 °C; Monomer concentration, mol L^−1^; ENB/DCPD, 1.0 mol L^−1^;

^b)^ Activity = 10^5^ g·mol^−1^·h^−1^;

^c)^ Monomer incorporation ratio, determined by ^1^H NMR;

^d)^ Determined by differential scanning calorimetry (DSC, second heating);

^e)^ *M*_n_ = 10^4^ g mol^−1^, *M*_n_ and PDI were determined by gel permeation chromatography (GPC) in 1,2,4‐trichlorobenzene at 160 °C;

^f)^ The insertion ratio of the polymer involving L3 unit is the sum of L3 inserted on one side and L3 inserted on both sides. L3 inserted on both sides takes proportion of 17.5%, 25%, 11.7%, 26.5% correspond to entries 6–8, 12 respectively.

### 2.3 Thermal and Mechanical Properties of Functionalized Polyolefins

The thermal properties of these copolymers were studied by differential scanning calorimetry (DSC), which showed that the binary copolymers (E‐L1^0.5^, E‐L2^0.5^, E‐L2^1.0^, E‐L3^0.5^_,_ and E‐L4^0.5^, correspond to the polymers prepared under the conditions shown in Table 1, entries 1–3, 6, and 9 respectively, where the superscript represents the comonomers concentration) possessed high melting points (118.8–124.2 °C), exhibiting characteristic thermal properties of plastic materials. The introduction of cyclic monomers DCPD or ENB reduced the copolymers’ crystallinity and melting point, transforming the plastic polymers into elastomers, and resulting in glass transition temperatures from −7.5 to 24.6 °C.

**Figure 2:**
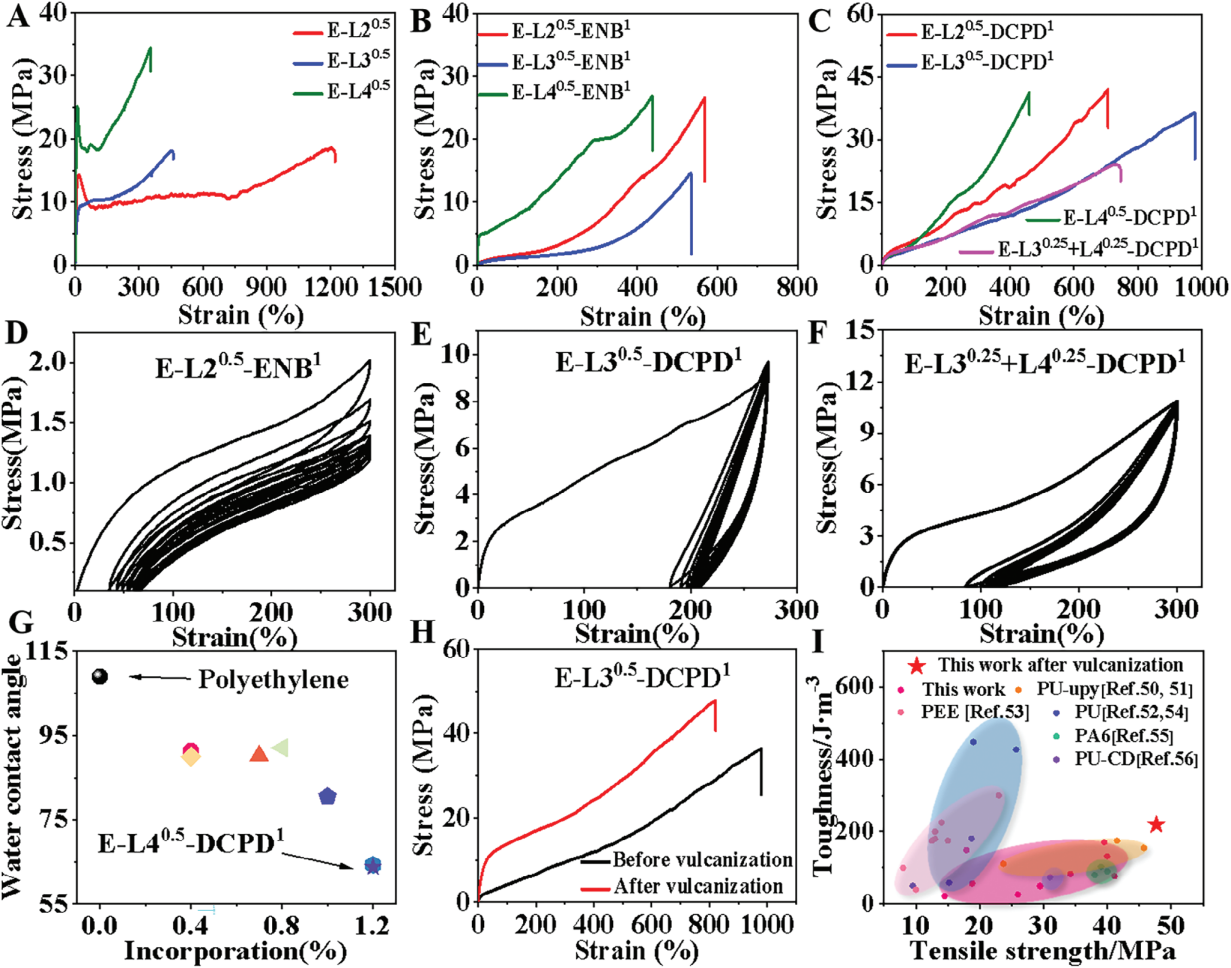
A) Stress‐strain curves for selected binary copolymers (E‐L2^0.5^, E‐L3^0.5^, and E‐L4^0.5^). B) Stress–strain curves for selected terpolymers (E‐L2^0.5^‐ENB^1^, E‐L3^0.5^‐ENB^1^, and E‐L4^0.5^‐ENB^1^). C) Stress–strain curves for selected terpolymers/tetrapolymers (E‐L2^0.5^‐DCPD^1^, E‐L3^0.5^‐DCPD^1^, E‐L4^0.5^‐DCPD^1^, and E‐L3^0.25^+L4^0.25^‐DCPD^1^). D–F) Plots of hysteresis experiments with 10 cycles at a strain of 300 % for E‐L2^0.5^‐ENB^1^, E‐L3^0.5^‐DCPD^1^, and E‐L3^0.25^+L4^0.25^‐DCPD^1^. G) Scatterplot of spiropyran monomer incorporation ratio versus water contact angles (WCA) for selected terpolymers/tetrapolymers. H) Stress‐strain curves for the terpolymer E‐L3^0.5^‐DCPD^1^ before and after vulcanization. I) The toughness and stress values of functional polymers with stimulus‐responsive color changes reported in previous literature, including polyurethane (PU), polyurethanes with multiple hydrogen bonds (PU‐upy), polyether‐ester‐urethane (PEE), PU‐carbon dot cross‐linked (PU‐CD), polyamide 6 (PA6), and so on.

Tensile tests were carried out to investigate the mechanical properties of these functionalized polyolefins (**Figure** **2**). The binary copolymers (E‐L2^0.5^, E‐L3^0.5^, and E‐L4^0.5^) exhibited significant plasticity, with Young's moduli of 520, 210, and 560 MPa, respectively. They also exhibited great stress at break (18–35 MPa) and strain at break (350–1200%) (Figure 2A, and Figures S16 and S17, Supporting Information). Terpolymers/tetrapolymers (E‐L2^0.5^‐ENB^1^, E‐L2^0.5^‐DCPD^1^, E‐L3^0.5^‐ENB^1^, E‐L3^0.5^‐DCPD^1^, E‐L4^0.5^‐ENB^1^, E‐L4^0.5^‐DCPD^1^, and E‐L3^0.25^+L4^0.25^‐DCPD^1^, correspond to the polymers prepared under the conditions shown in Table 1, entries 4, 5, 7, 8, and 10–12 respectively, where the superscript represents the comonomers concentration.) also exhibited outstanding tensile properties, with stress and strains reaching 40 MPa and 980%, respectively (Figure 2B,C). Except for E‐L4^0.5^‐ENB^1^, all samples exhibited elastomeric properties due to the random copolymer unit of ethylene and comonomers, which served as the “soft” unit in these copolymers. The crystalline polyethylene unit served as the “hard” unit and physical crosslinking point. These interactions improved the elasticity and tensile properties of the copolymers. In addition, the incorporation of cyclic monomers increased the rigidity of the polymer main chain and its mechanical strength. The cyclic tensile tests (the polymer sample was loaded and unloaded 10 times under 300% strain) of these elastomers also demonstrated elastic recovery rates of up to 78% (Figure 2D–F, and Figures S18 and S19, Supporting Information).

### 2.4 Surface Properties

The surface properties of these copolymers were studied by measuring their water contact angles (WCA; Figure S20, Supporting Information), which showed that the hydrophilicity of the SP‐based copolymers was significantly higher compared with those of pure polyethylene due to the introduction of polar groups (Figure 2G). Compared with polyethylene (109°), incorporating these polar comonomers significantly reduced the WCA to 63°. The high content of SP groups in the terpolymers containing comonomer L4 contributed to their much lower WCA values.

### 2.5 Sulfur Vulcanization

Sulfur vulcanization is one of the most widely used industrial strategies to improve the mechanical properties of synthetic rubber.^[^ ^38^ ^]^ Within these synthesized copolymers, the introduction of a diene‐type norbornene comonomer provided reactive sites for vulcanization. Sulfur vulcanization studies were performed for terpolymer E‐L3^0.5^‐DCPD^1^ using a sulfur‐containing commercial formula. The stress‐strain curve in Figure 2H shows a significant increase in Young's modulus and tensile strength after vulcanization, with a slight decrease in strain. The application of industrial vulcanization technology also gives these newly developed functional polyolefin materials practical application potential. The toughness and stress of the vulcanized terpolymer E‐L3^0.5^‐DCPD^1^ reached 220 MJ m^−3^ and 48 MPa. SP‐based responsive polymers have been widely studied in the literature.^[^ ^45^, ^49^, ^50^ ^]^ These newly developed functional polyolefins are among the best materials with a great balance between high stress and high toughness (Figure 2I and Figure S21, Supporting Information).^[^ ^51^, ^52^, ^53^, ^54^, ^55^, ^56^, ^57^ ^]^

### 2.6 Photo‐Thermochromism of Functionalized Polyolefins

**Figure 3:**
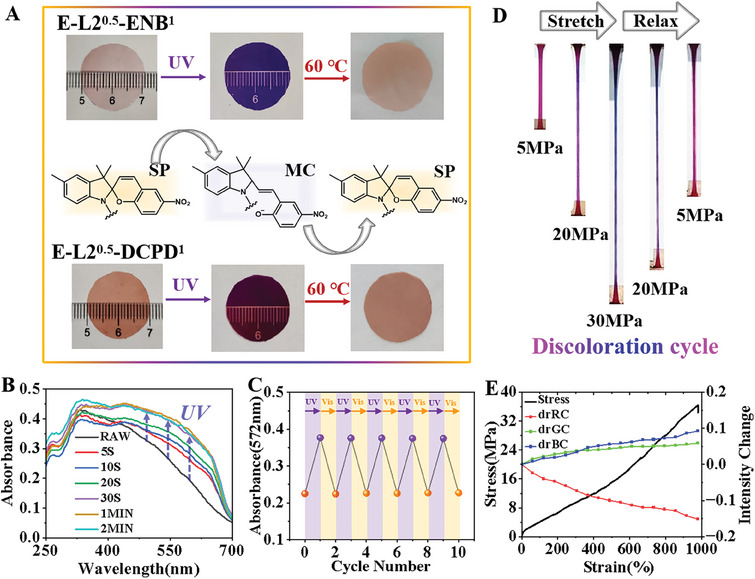
A) Photo‐thermochromism of the terpolymers of E‐L2^0.5^‐ENB^1^ and E‐L2^0.5^‐DCPD^1^. B) UV–vis absorption spectra of E‐L2^0.5^‐DCPD^1^. Different curves refer to how long the sample was exposed to UV radiation, where the black line represents the UV absorption spectrum without irradiation. C) Photofatigue resistance of terpolymer E‐L2^0.5^‐DCPD^1^ upon alternating UV and visible‐light irradiation. D) Mechanical response discoloration cycle of terpolymer E‐L3^0.5^‐DCPD^1^. The sample was stretched to a stress of 30 MPa at 10 mm min^–1^, and then the stress was released. Color changes were recorded at stress values of 5, 20, 30, 20, and 5 MPa in sequence. E) Change in the intensity in drRC, drGC, and drBC as a function of strain of the terpolymer E‐L3^0.5^‐DCPD^1^.

Photochromic or thermochromic polymer materials can undergo color responses triggered by light irradiation or upon heating, which may find applications in information storage/transmission or sensors.^[^ ^58^ ^]^ In this work, a photo/thermochromic SP group was introduced into the polyolefin molecular chain to render these copolymers light/heat‐responsive (Figure S22, Supporting Information). Terpolymers containing cyclic monomers (ENB or DCPD) exhibited outstanding light response due to their greater transparency (Figure S23, Supporting Information). For example, the terpolymers E‐L2^0.5^‐ENB^1^ and E‐L2^0.5^‐DCPD^1^ were cut into circular thin films with a diameter of 1 cm and a thickness of 0.1 mm. The color states of the films were recorded before and after irradiation under UV light for 30 s. As shown in **Figure** **3**A, these polymers became discolored as the pattern covered with a ruler became visible. The terpolymer E‐L2^0.5^‐ENB^1^ containing ENB appeared as indigo blue after irradiation, while the terpolymer E‐L2^0.5^‐DCPD^1^ containing DCPD appeared as wine red. Subsequently, the discolored polymer film returned to its initial state after being heated at 60 °C, completing one photothermal response cycle. The color response of these terpolymers originated from the transition between the SP and MC states of the SP group in the polymer. In addition, UV–vis absorption spectra showed that the terpolymer E‐L2^0.5^‐DCPD^1^ achieved discoloration in about 30 s (Figure 3B). Subsequently, the photofatigue resistance of the terpolymer E‐L2^0.5^‐DCPD^1^ was studied by monitoring its absorbance at 572 nm under alternating UV and visible light irradiation (five cycles) (Figure 3C). Even after five cycles of UV and visible light irradiation, the efficiency of the photoinduced SP/MC transformation was almost unaffected, indicating excellent photofatigue resistance. The uniform distribution of the SP functional group in the polymer matrix achieved via direct copolymerization may be a key reason for their homogeneous photo‐thermochromism.

### 2.7 Mechanochromism of Functionalized Polyolefins

In addition to light and heat stimuli, force is another main source of external stimulus for intelligent responsive polymer materials.^[^ ^59^ ^]^ The key to obtaining a mechanochromic material based on a SP unit is the transfer of mechanical force stimulus to the carbon‐oxygen bonds in the helical ring structure.^[^ ^60^ ^]^ Therefore, the crosslinkable comonomer L3 was introduced into the polymer network to give it mechanochromism (Movie S1, Supporting Information). For a better demonstration, we carried out one mechanical response discoloration cycle on a tensile tester with terpolymer E‐L3^0.5^‐DCPD^1^ (Figure 3D). As the elongation and stress increased, the polymer spline narrowed and changed from purple to blue. Subsequently, the blue color disappeared during stress recovery and then changed back to purple. This indicated that the SP group was covalently bonded to the polyolefin main chain, allowing it to conduct the force and guide the dissociation of the SP ring, thus achieving mechanochromism. To quantify the color change and relative strain‐dependent activation of SP in terpolymer E‐L3^0.5^‐DCPD^1^, the corresponding intensity change of red (drRC), green (drGC), and blue channels (drBC) are shown in Figure 3E as a function of the strain of the sample after being subjected to a force. The results show that the red intensity decreased almost linearly, while the blue and green intensities increased linearly. In addition to directly synthesizing responsive polyolefins, the sulfurized sample also achieved mechanochromism (Figures S24 and S25, Supporting Information).

### 2.8 Multiple‐Stimuli Responses of Functionalized Polyolefins

**Figure 4:**
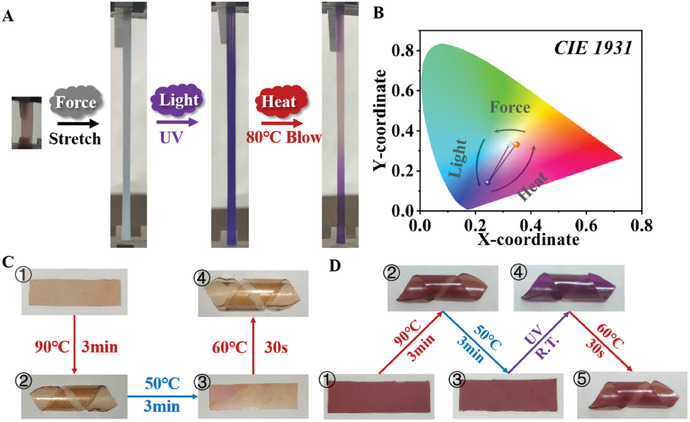
A) The tetrapolymer E‐L3^0.25^+L4^0.25^‐DCPD^1^ underwent color changes under force, light, and heat stimuli in sequence. B) The digital image analysis of the tetrapolymer E‐L3^0.25^+L4^0.25^‐DCPD^1^ under force, light, and heat stimuli in sequence. C) Shape memory function in response to heat stimulation using the copolymer E‐L2^0.5^‐DCPD^1^. 1) The raw spline. 2) Wound around a glass rod and heated to 90 °C to obtain a spiral shape. 3) Tiling and cooling. 4) Heating to restore the spiral shape. D) Shape memory function in response to light stimulation at room temperature using the copolymer E‐L3^0.25^+L4^0.25^‐DCPD^1^. 1) The raw spline. 2) Wound around a glass rod and heated to 90 °C to obtain a spiral shape. 3) Tiling and cooling. 4) Exposure to UV radiation at room temperature. 5) Heat to restore sample color.

The above‐mentioned photochromic and mechanochromic polymers were prepared by the incorporation of photothermal‐responsive or force‐responsive vinyl functional monomers. Subsequently, comonomers L3 and L4 were simultaneously introduced into the polyolefin backbone, thus achieving a polyolefin with multiple responses to light, mechanical, and thermal stimulation. As shown in **Figure** **4**A, the tetrapolymer E‐L3^0.25^+L4^0.25^‐DCPD^1^ was sequentially subjected to force, UV, and thermal stimuli, and the sample exhibited different color changes (Movie S2, Supporting Information). Digital image analysis also showed the average color coordinate movement of the sample under different external stimuli (Figure 4B and Figure S26, Supporting Information).

### 2.9 Shape Memory Properties of Functionalized Polyolefins

Movement provides another response mode for polymers in addition to color changes for stimuli‐responsive polymers.^[^ ^61^ ^]^ Shape memory polymers stimulated by heat or light have attracted significant attention.^[^ ^62^ ^]^ Here, we adjusted the incorporation ratio of the third monomer (DCPD) based on the above photo‐thermochromic terpolymer to synthesize a polymer with an appropriate chain structure and crystallinity to increase its shape memory properties. This enabled the polymer to achieve a color response and respond to polymer movement. For example, E‐L2^0.5^‐DCPD^1^ (*T*_g_ =  −7.5 °C and *T*_m_ =  81.6 °C) was shaped at a temperature above *T*_m_ (90 °C), tiled at 50 °C, and then cooled to room temperature. Its original shape was restored by thermal stimulation (60 °C, Figure 4C). This was because when the temperature rose to the deformation temperature of the material, the crystals that acted as physical cross‐linking sites in the terpolymer melted, and the molecular chains could relatively slip under the action of an external force. When the temperature decreased below the deformation temperature of the material, along with the removal of external force, the terpolymer recrystallized, and its temporary shape was fixed. When heated again, the material returned to its original shape under the action of an internal driving force.

More importantly, the shape memory function of the prepared tetrapolymer E‐L3^0.25^+L4^0.25^‐DCPD^1^ could be achieved with similar results under light stimulation at room temperature (Figure 4D and Movie S3, Supporting Information). When stimulated by UV light, the SP groups absorbed the light, thus releasing internal stress under energy provided by the UV light. On the other hand, the SP group of the L3 unit underwent isomerization under UV irradiation, serving as the cross‐linking point of the polymer network to induce molecular chain motion. This may have accelerated the conduction of internal stress within the polymer and restored its initial shape. The tetrapolymer E‐L3^0.25^+L4^0.25^‐DCPD^1^ showed obvious discoloration after irradiation, and further heating restored its color while keeping its shape unchanged at 60 °C. This novel polyolefin elastomeric material could interconvert between mechanical or color signals under thermal and/or light stimulation (Figures S27 and S28, Supporting Information).

### 2.10 Application to Commercial Polymers

**Figure 5:**
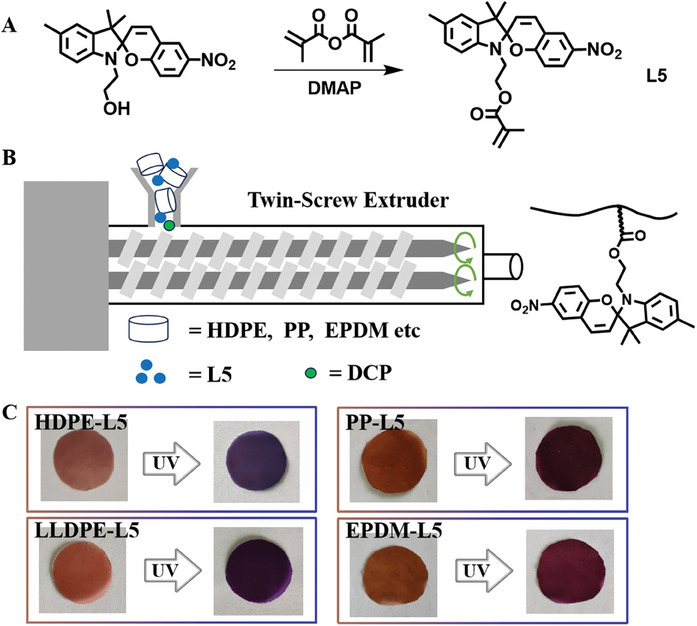
A) Synthesis schematic of monomer L5. B) Use of a twin screw extruder to convert a commercial polymer into a photochromic polymer. C) Photochromism of the selected blend samples (HDPE‐L5, PP‐L5, LLDPE‐L5, and EPDM‐L5). The color changes of all samples were recorded after exposure to UV radiation for 30 s.

Many commercial polymers are difficult to insert with SP through coordination polymerization, so we explored the possibility of postpolymerization functionalization.^[^ ^63^ ^]^ Reactive extrusion using a twin screw extruder is an efficient strategy to access grafted polymers with great commercial potential.^[^ ^64^ ^]^ A methyl acrylate‐based SP monomer (L5, **Figure** **5**A) was prepared and characterized. This monomer could be easily grafted to a series of commercial polymers (including HDPE, LDPE, PP, and EPDM) in one step to prepare stimulus‐responsive polymers (Figure 5B and Figure S29, Supporting Information). FTIR spectroscopy indicated the successful installation of SP groups under general blending conditions). All of these grafted commercial polymers achieved color signal transmission under light stimulation (Figure 5C).

## 3 Conclusion

A series of SP‐functionalized polyolefin thermoplastics or elastomers were prepared via late transition metal‐catalyzed copolymerization. By adjusting the structure of SP‐based comonomers and the content of the third comonomer (ENB and DCPD), light, force, and thermal responsivity could be installed and tuned. These functionalized polyolefin materials demonstrated versatile photochromic, thermochromic, mechanochromic, and shape memory functions. More importantly, the shape memory function of functionalized polyolefin could be achieved under light stimulation at room temperature. The introduction of a polar SP functionality and the high molecular weight of these polyolefins ensured their excellent surface and mechanical properties. Finally, reactive extrusion using a twin screw grafting strategy was used to endow commercial polyolefins with stimulus‐responsive properties.

## Conflict of Interest

The authors declare no conflict of interest.

## Author Contributions

W.Q. and Z.B. contributed equally to the work. C.C. conceived the project. C.Z. performed experiments regarding the synthesis and characterization. All authors contributed to data analysis and paper writing.

## Supporting information

Supporting Information

Supplemental Movie 1

Supplemental Movie 2

Supplemental Movie 3

## Data Availability

The data that support the findings of this study are available in the supplementary material of this article.
