# Supplementary material for: Light, Heat, and Force‐Responsive Polyolefins
Source: Adv Sci (Weinh). 2024 Jan 6;11(11):2307568. doi: 10.1002/advs.202307568 (PMC10953547; doi:10.1002/advs.202307568)
Supplement: Supplementary file 1 — Supporting Information [file ADVS-11-2307568-s003.pdf]

ADVANCED SCIENCE
Open Access

Supporting Information

for *Adv. Sci.*, DOI 10.1002/advs.202307568

Light, Heat, and Force-Responsive Polyolefins

Weicheng Qu, Zhengxing Bi, Chen Zou and Changle Chen**

Supporting Information

Light, Heat, and Force-Responsive Polyolefins

Weicheng Qu, Zhengxing Bi, Chen Zou, Changle Chen**

Table of Contents

1. General methods......3
2. Experimental Section 6
2.1. Monomer synthesis process 6
2.2. Procedure for polymerization 18
2.3. Blending modification method 18
2.4. The synthesis and NMR spectrum of PO-Pd catalyst 20
3. Results on multiple specimens are shown for reproducibility......21
4. The cyclic tensile tests 22
5. Water contact angle of the polymer samples 24
6. Supplementary illustration......25
7. Shape memory properties of functionalized polyolefins......28
8. Photochromic ability of blended samples......29
9. The relationship between the introduction of spiropyran-based monomers and the variation of UV absorption......30
10. ^{1}H NMR and ^{13}C NMR of copolymer 33
11. DSC of copolymer......45
12. GPC of copolymer 51
13. References 57

1. General methods

All experiments were carried out under a dry nitrogen atmosphere using standard Schlenk techniques or in a glovebox. Deuterated solvents used for NMR spectroscopy were dried and distilled prior to use. ^{1}H, ^{13}C NMR spectra were recorded on a Bruker Ascend Tm 600 spectrometer at ambient temperature unless otherwise stated. The chemical shifts of the ^{1}H and ^{13}C NMR spectra were referenced to tetramethylsilane. Samples were preheated for at least 30 min before acquiring data. Molecular weight and molecular weight distribution of the polymer were determined by gel permeation chromatography (GPC) with a PL-220 equipped with two Agilent PLgel Olexis columns at 160 ^{o}C using trichlorobenzene as a solvent, and the calibration was made using polystyrene standard and are corrected for linear polyethylene by universal calibration using the Mark–Houwink parameters of Rudin: K = 1.75 × 10^{-2} cm^3/g and R = 0.67 for polystyrene and K = 5.90 × 10^{-2} cm^3/g and R = 0.69 for polyethylene. Dichloromethane, THF, and hexanes were purified in solvent purification systems. DSC measurements were performed on a TA Instruments DSC Q10. Samples (ca. 5 mg) were annealed by heating to 150 °C at 10 °C /min, cooled to -50 °C at 10 °C /min, and then analyzed while being heated to 150 °C at 10 °C /min. UV−vis absorption spectra and transparency characterization were obtained using a Shimadzu 3600 spectrophotometer. The scanning range of the wavelength was 400 ~ 800 nm. The CET-HXUV 300 (50 W, 100-240 V, 200-2500 nm) was used as light source.

Mechanical properties of the polyethylene sample. Stress/strain experiments were performed at room temperature at 10 mm/min using a UTM2502 universal tester. At least three specimens of each polymer were tested. The test specimens had the following dimensions, gauge length: 28 mm; width: 2 mm; thickness: 0.5 mm.

Water contact angle measurement. Water contact angles on polymer films were measured with Contact Angle Meter SL200B (Solon Tech. Co. Ltd.) by the dynamic sessile drop method. Samples for water contact angle measurements were prepared by the evaporation of 3 to 5 % (w/w) solutions in toluene onto glass slides under ambient conditions. The solvent was evaporated on top of a glass slide for 10 minutes, and a second layer of the polymer solution was then applied in order to make the film thicker. The water contact angles of the polymer thin films were measured using a contact angle goniometer at 25 °C with an accuracy of ±3°. The reported values are the average of at least six measurements made at different positions of the film.

Preparation of sulfur vulcanized crosslinked polymer. A total amount of 3.0 g copolymer was dissolved in 100 mL of THF at 60 ℃ under nitrogen. After 30 min of stirring, add

formulation (ie 150 mg ZnO, 30 mg stearic acid, 15 mg accelerator MBT, accelerator 20 mg TMTD, 30 mg sulfur), the solvent was drained and dried under vacuum at 45 °C for 36 hours. The rubber mixture was thermoformed using a hydraulic press at 10 MPa, 160 °C, and cured for 20 minutes to produce a 1 mm thick sheet for subsequent use.

Discoloration in response to optical/thermal stimulation measurement. The CET-HXUV 300 (50 W, 100-240 V, 200-2500 nm) was used as light source. All the materials were cut into round films with a diameter of 1 cm and a thickness of 0.1 mm and irradiated under ultraviolet light for 30 s. The color state before and after irradiation was recorded using a Shimadzu 3600 spectrophotometer. The photo-fatigue resistance of the ternary copolymer first record the ultraviolet absorption value of the original polymer at 572 nm, then record after 30 seconds of ultraviolet light, and then record after visible light irradiation until the polymer returns to its original color. This process was repeated five times.

Discoloration in response to mechanical stimulation measurement. The experiment was performed at room temperature at 10 mm/min using a UTM2502 universal tester. The test specimens had the following dimensions, gauge length: 28 mm; width: 2 mm; thickness: 0.5 mm. The sample was stretched at a constant speed from 0 to 30 MPa, then at the same speed contracted back to 0 MPa, and the discoloration process was photographed and recorded.

RGB color analysis Mechanical activation was analyzed using RGB color analysis. Optical images were taken by a NIKON Z5 camera, photos were taken every 30 s as the samples were strained. The photos were analyzed in Adobe Photoshop CS6 Software. After the background was white balanced, the average intensity of red (R), green (G) and blue (B) channel at the region of interest was obtained and used for calculating RGB ratio, RGB ratio was calculated as $rRC = R/(R + G + B)$, $rGC = G/(R + G + B)$ and $rBC = B/(R + G + B)$. The intensity change in each channel, drRC, drGC and drBC were obtained by subtracting the initial RGB ratio from the RGB ratio at each point, respectively.

Multiple stimulus responses measurement. The test specimens had the following dimensions, gauge length: 28 mm; width: 5 mm; thickness: 0.5 mm. Tetrapolymer **E-L3$^{0.25}$+L4$^{0.25}$-DCPD[1]** was stretched to 500% strain, showing the mechanochromism. Then, under the tensile force, the sample was covered under ultraviolet light for 30 seconds, and its current color state was recorded. Then, the middle part of the sample was blown with a digital display hot air gun set to 80 ℃ for 30 seconds, the color fading was observed, and the color changes in the middle part of the sample were recorded.

Discoloration shape memory in response to optical/thermal stimulation measurement. Thermal stimulation: The test specimens had the following dimensions, gauge length: 40 mm;

width: 10 mm; thickness: 0.5 mm. For a sample with glass transition temperature of -7.5℃ and melting point of 81.6 ℃ (**E-L2$^{0.5}$-DCPD^{1}**), it can be formed by hot pressing for 3 min at 90 ℃, tiled at 50 ℃ and then cooled to room temperature, and the original shape can be restored by heating the sample again (60 ℃).

Optical stimulation: The CET-HXUV 300 (50 W, 100-240 V, 200-2500 nm) was used as light source. The test specimens had the following dimensions, gauge length: 40 mm; width: 10 mm; thickness: 0.5 mm. **E-L3$^{0.25}$+L4$^{0.25}$-DCPD^{1}** can be formed by hot pressing for 3 min at 90 ℃, tiled at 50 ℃ and then cooled to room temperature, irradiate 5 min under ultraviolet light instead of heating, restore the original shape without the need for other auxiliary energy. While restoring the original shape, the polymer will turn purple, proving that spiropyran is activated to the color part anthocyanin. Heating the sample again (no high temperature is needed), and the color of the sample can be restored.

2. Experimental Section

2.1. Monomer synthesis process

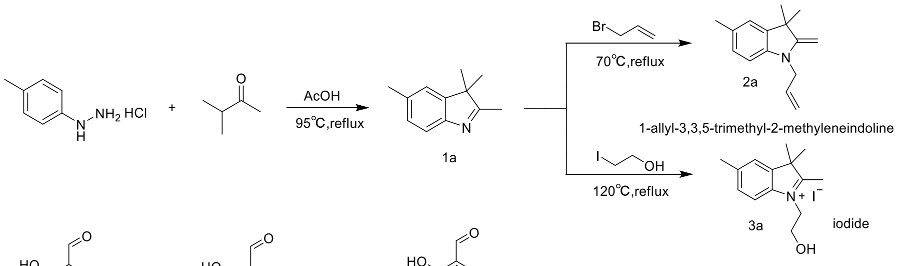

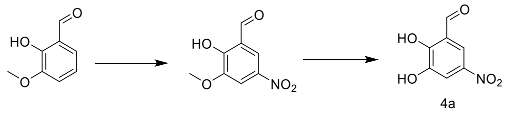

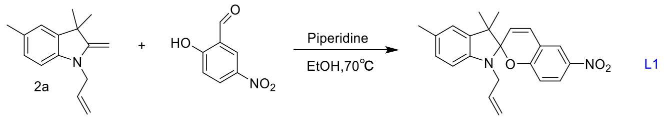

1'-allyl-3',3',5'-trimethyl-6-nitrospiro[chromene-2,2'-indoline]

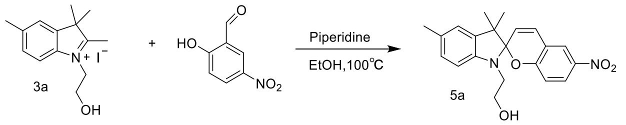

2-(3',3',5'-trimethyl-6-nitrospiro[chromene-2,2'-indolin]-1'-yl)ethan-1-ol

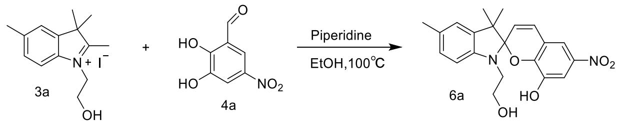

1'-(2-hydroxyethyl)-3',3',5'-trimethyl-6-nitrospiro[chromene-2,2'-indolin]-8-ol

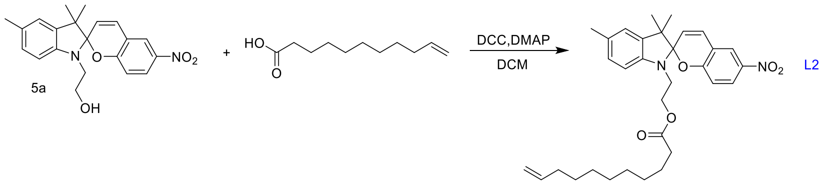

2-(3',3',5'-trimethyl-6-nitrospiro[chromene-2,2'-indolin]-1'-yl)ethyl undec-10-enoate

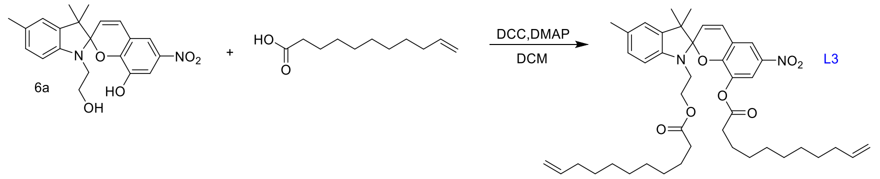

3',3',5'-trimethyl-6-nitro-1'-(2-(undec-10-enoyloxy)ethyl)spiro[chromene-2,2'-indolin]-8-yl undec-10-enoate

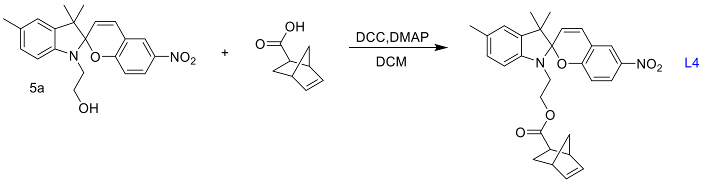

2-(3',3',5'-trimethyl-6-nitrospiro[chromene-2,2'-indolin]-1'-yl)ethyl (1*S*,2*R*,4*S*)-bicyclo[2.2.1]hept-5-ene-2-carboxylate

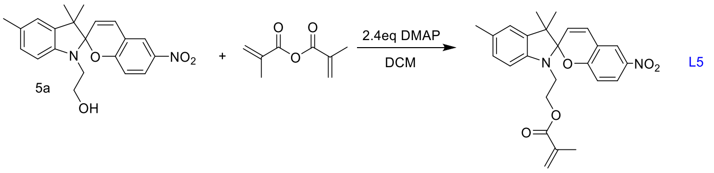

2-(3',3',5'-trimethyl-6-nitrospiro[chromene-2,2'-indolin]-1'-yl)ethyl methacrylate

Scheme S1. Schematic diagram of the synthesis of all the monomers involved in this paper.
The compounds of **1a**, **2a**, **3a**, **4a**, **5a**, **6a** were prepared according to literature procedure[1-3] or with some modification.

Compound (**1a**): 2,3,3,5-tetramethyl-3H-indole
The 4-Methyl-phenyl hydrazine hydrochloride (10 g, 63.3 mmol, 1 equiv) and methyl isopropyl ketone (13.6 mL, 126.6 mmol, 2 equiv) were dissolved in 100 mL absolute acetic acid and heated to reflux using a reflux condenser under N_2 pressure at 100 ℃. After refluxing for 5.5 h, the solution was added in 100 ml H_2O to dilute the mixture. Then slowly drop the NaOH solution (1 mol/L) into the solution until the pH is 7. After the temperature drops, the crude product is extracted with DCM and dried in vacuum to yield **1a** as a brown liquid (9.85 g, 56.8 mmol, 90%). The product does not need further purification and can proceed to the next step.

Compound (**2a**): 1-allyl-3,3,5-trimethyl-2-methyleneindoline
Compound **1a** (5g, 28.9 mmol, 1 equiv.) were dissolved in 3-bromopropene (5 g, 41.7 mmol, 1.44 equiv) and heated to reflux using a reflux condenser under N_2 pressure at 75 ℃. After heating for 24 h, the solution was added in 50 ml H_2O to dilute the mixture. Then slowly drop the NaOH solution (0.1 mol/L) into the solution until the pH is 10. The resulting precipitate was filtered and washed with absolute ether three times. The crude product was purified by column chromatography and dried in vacuum to yield **2a** as a light brown solid (4.44g, 20.8 mmol, 72%).

Compound (**3a**): 1-(2-hydroxyethyl)-2,3,3,5-tetramethyl-3H-indolium iodide
Compound **1a** (5 g, 28.9 mmol, 1 equiv) and 2-iodoethanol (11.3 mL, 144.5 mmol, 5 equiv) was dissolved in 50 ml acetonitrile, fitted with a reflux condenser, and placed in a preheated 120 °C oil-bath under N_2. After heating for 2.5 h, the resulting viscous dark fluid was added dropwise to rapidly stirring EtOAc. The resulting precipitate was filtered and washed with minimal EtOAc to yield **3a** as a light brown powder (6.07 g, 17.6 mmol, 69 %).

Compound (**4a**): 2,3-dihydroxy-5-nitro-benzaldehyde
Prepared according to literature procedure. [1]
Compound (**5a**): 2-(3',3',5'-trimethyl-6-nitrospiro[chromene-2,2'-indolin]-1'-yl) ethan-1-ol

A solution of **2a** (6.0g, 17 mmol, 1 equiv.), and 5-nitrosalicylaldehyde (2.8g, 17 mmol, 1 equiv.) and piperidine (1.7 mL, 17mmol, 1 equiv.) in a certain amount of ethanol was heated to reflux at 100 °C. After stirring under N_2 for 5 h, the reaction was cooled to room temperature and was kept in the refrigerator overnight. After filtration, the precipitate was washed with cold MeOH three times, and dried to yield **5a** as a purple solid (5.29 g, 14.4 mmol, 85%).

Compound (**6a**): 1'-(2-hydroxyethyl)-3',3',5'-trimethyl-6-nitrospiro[chromene-2,2'-indolin]-8-ol

A solution of **3a** (5.0 g, 14.5 mmol, 1 equiv.), and **4a** (2.65 g, 14.5 mmol, 1 equiv.) and piperidine (2.85 mL， 29.0 mmol, 2 equiv.) in a certain amount of ethanol was heated to reflux at 100 °C. After stirring under N_2 for 5 h, the reaction was cooled to room temperature and was kept in the refrigerator overnight. The resulting precipitate was filtered and washed with cold MeOH three times. The crude product was purified by column chromatography and dried in vacuum to yield **6a** as a dark-green solid (3.48 g, 9.1 mmol, 62.7%).

Compound (**L1**): 1'-allyl-3',3',5'-trimethyl-6-nitrospiro[chromene-2,2'-indoline]

A solution of **2a** (5.12g, 24 mmol, 1 equiv.), and 5-nitrosalicylaldehyde (4.5g, 27 mmol, 1.1 equiv.) and piperidine (2.4mL, 24.2 mmol, 1 equiv.) in a certain amount of ethanol was heated to reflux at 80 °C. After stirring under N_2 for 24 h, the reaction was cooled to room temperature and was kept in the refrigerator overnight. After filtration, the precipitate washed with cold MeOH, and dried to yield **L1** as a yellow-green solid (5.46 g, 15.2 mmol, 63.4%).

^{1}H NMR (400 MHz, Chloroform-d) δ 8.02 (d, J = 2.7 Hz, 1H), 8.00 (m, 1H), 6.99 – 6.86 (m, 3H), 6.74 (d, J = 8.9 Hz, 1H), 6.48 (d, J = 7.8 Hz, 2H), 5.86 (d, J = 10.4 Hz, 2H)，5.17 (dd, J = 17.2, 1.8 Hz, 1H), 5.07 (dd, J = 10.3, 1.8 Hz, 1H), 3.92 – 3.60 (m, 2H), 2.32 (s, 2H), 1.29 (s, 3H), 1.21 (s, 3H).

^{13}C NMR (101 MHz, Chloroform-d) δ 159.64, 144.70, 140.86, 136.10, 134.79, 129.02, 128.28, 127.94, 125.88, 122.69, 122.49, 121.80, 118.54, 115.74, 115.55, 107.47, 106.88, 52.70, 46.24, 26.19, 21.01, 19.85.

ESI-MS (m/z): $[M+H]^+$ Calcd for C22 H23 O3 N2 , 363.1703; Found: 363.1699.

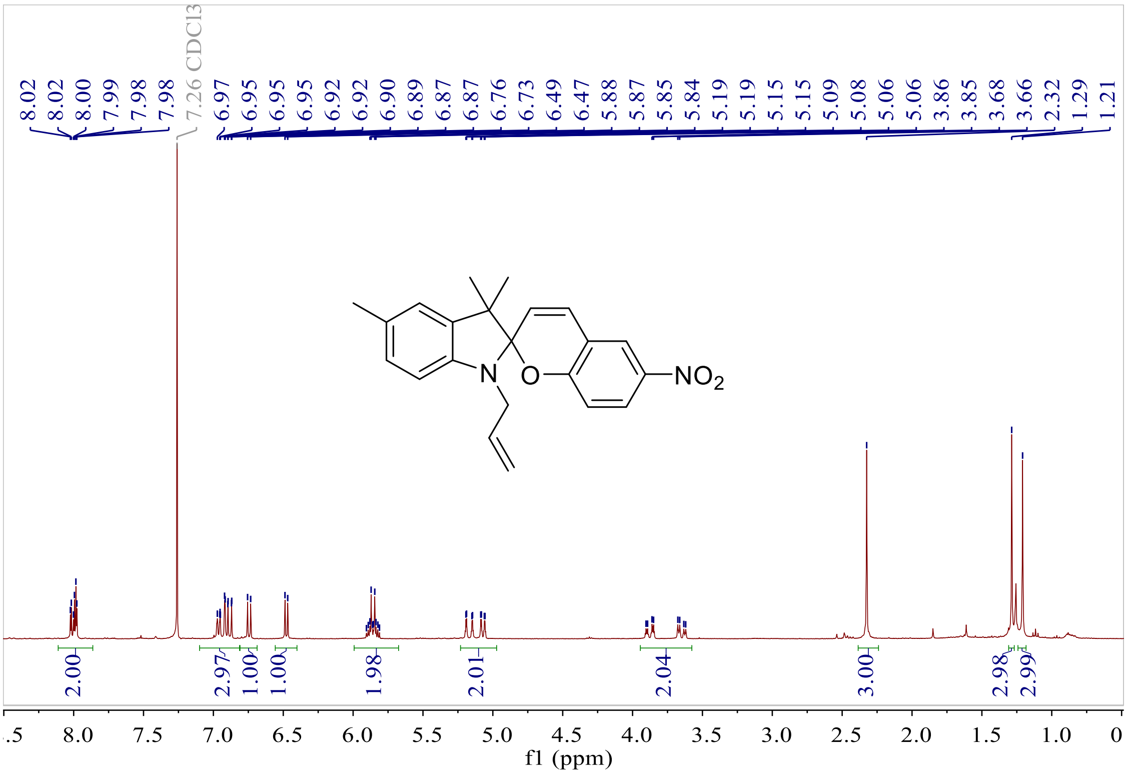

Figure S1. ^{1}H NMR spectrum of the comonomer L1.

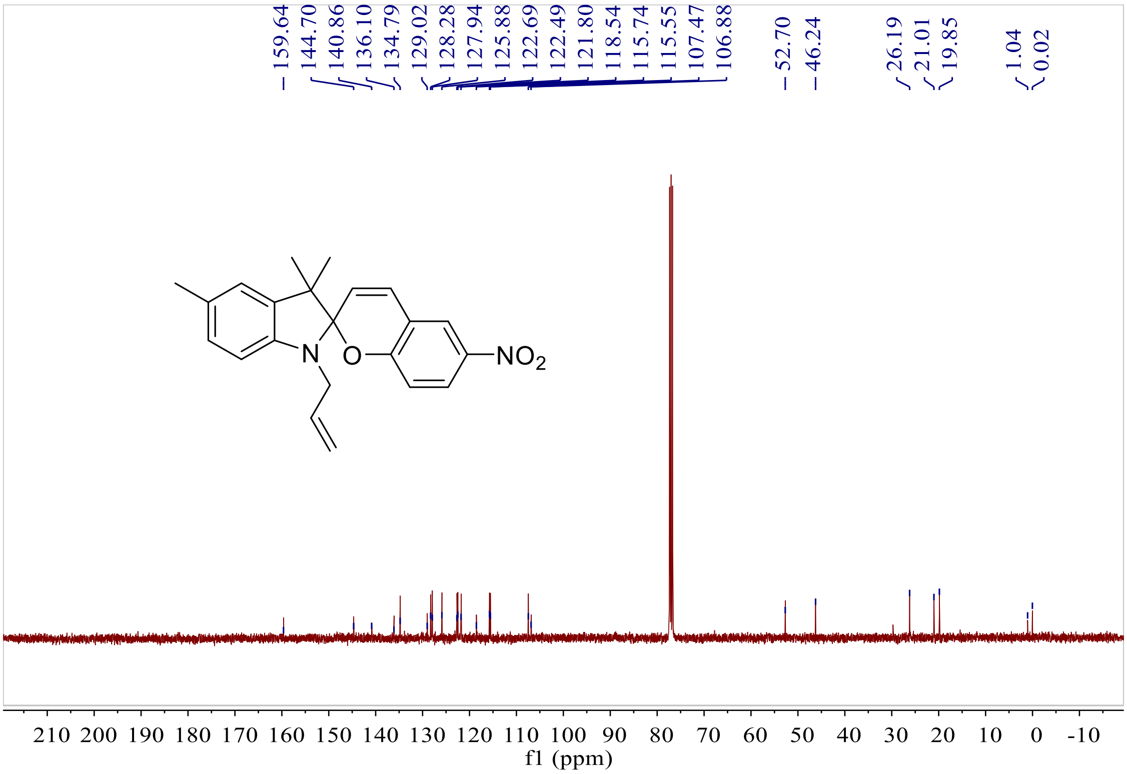

Figure S2. ^{13}C NMR spectrum of the comonomer **L1**.

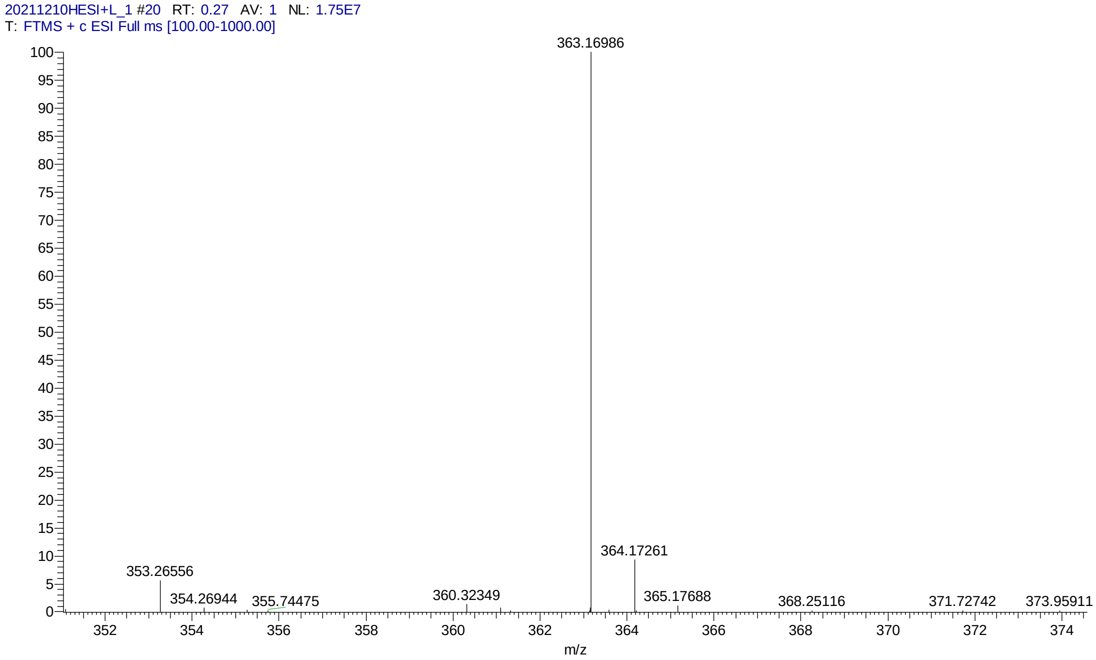

Figure S3. ESI-MS of the comonomer **L1**.

Compound (**L2**): 2-(3',3',5'-trimethyl-6-nitrospiro[chromene-2,2'-indolin]-1'-yl)ethyl undec-10-enoate

DCC (7.84 g，38 mmol, 1.4 equiv.) and 10-Undecenoic acid (6.54 g，35 mmol, 1.3 equiv.) were dissolved in dry DCM (100 mL) while stirring under N_2. DMAP (0.3 g, 2.5 mmol, 0.1 equiv.) in dry DCM (2 mL) and the compound **5a** (10.0 g, 27 mmol, 1 equiv.) in dry DCM (50 mL) were transferred to the reaction mixture. After stirring overnight, the crude product was isolated under reduced pressure using a rotary evaporator, which was recrystallized with MeOH, filtered, and dried to yield **L2** as a light gray-green solid (10.6 g, 20.4 mmol, 75.4%).

^{1}H NMR (400 MHz, Chloroform-d) δ 8.02 (d, J = 2.8 Hz, 1H), 8.00 (d, J = 1.5 Hz, 1H), 7.00 (d, J = 8.2 Hz, 1H), 6.93 (m, 2H), 6.75 (d, J = 8.7 Hz, 1H), 6.59 (d, J = 7.9 Hz, 1H), 5.88 (d, J = 10.4 Hz, 1H), 5.86 (m, 1H), 4.99 (dd, J = 17.1, 1.9 Hz,1H), 4.93 (dd, J = 10.3, 1.1 Hz, 1H), 4.20 (m, 2H), 3.46 (m, 1H), 3.37(m, 1H), 2.33 (s, 3H), 2.23 (t, J = 7.5 Hz, 2H), 2.03 (q, J = 7.1 Hz, 2H), 1.55 (m, 2H), 1.40 – 1.08 (m, 16H).

^{13}C NMR (100 MHz, Chloroform-d) δ 173.63, 159.52, 144.55, 140.98, 139.14, 135.78, 129.22, 128.19, 128.04, 125.92, 122.73, 122.66, 121.88, 118.45, 115.51, 114.17, 106.77, 106.56, 62.33, 52.84, 42.55, 34.16, 33.77, 29.24, 29.18, 29.07, 29.03, 28.86, 25.89, 24.82, 20.96, 19.81.

ESI-MS (m/z): $[M+H]^+$ Calcd for C32 H41 O5 N2 , 533.301; Found: 533.30042.

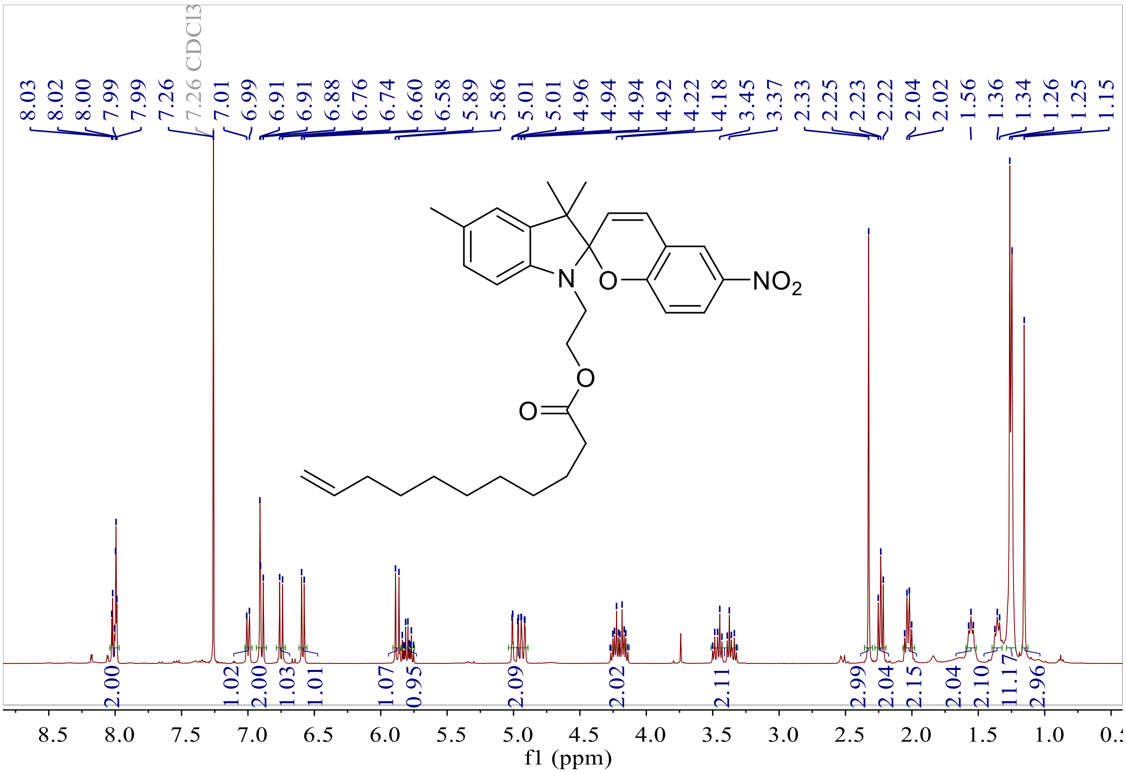

Figure S4. ^{1}H NMR spectrum of the comonomer **L2**.

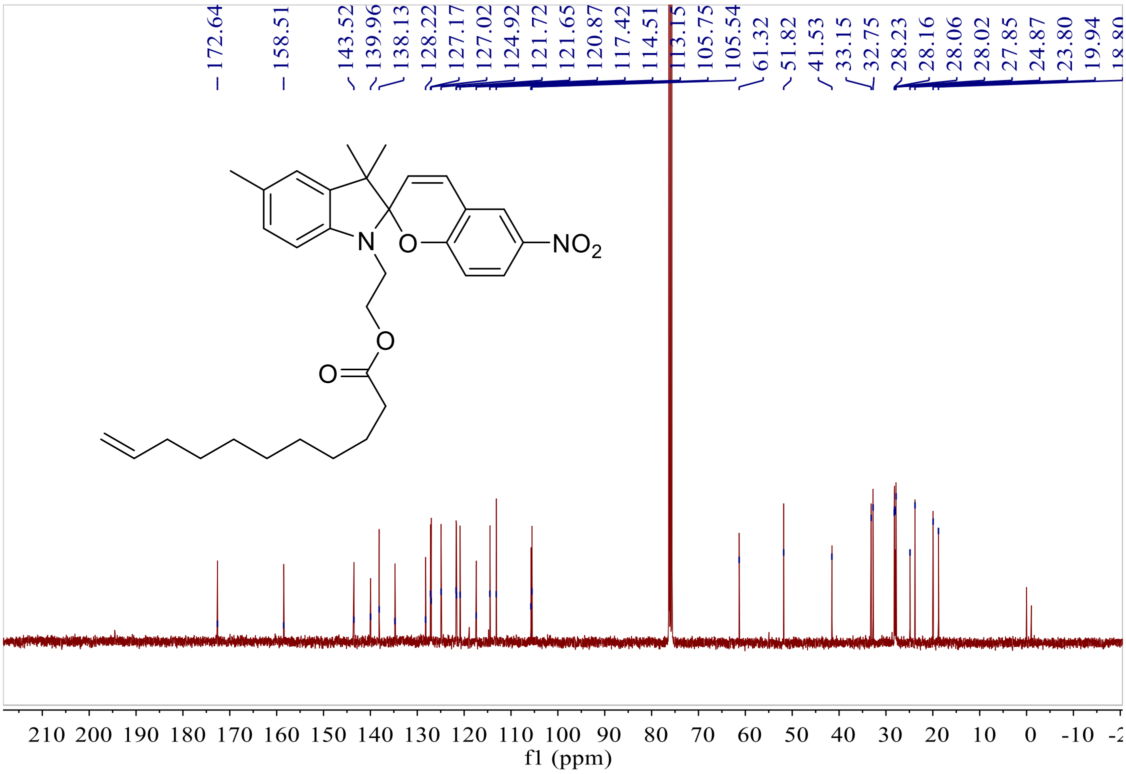

Figure S5. ^{13}C NMR spectrum of the comonomer **L2**.

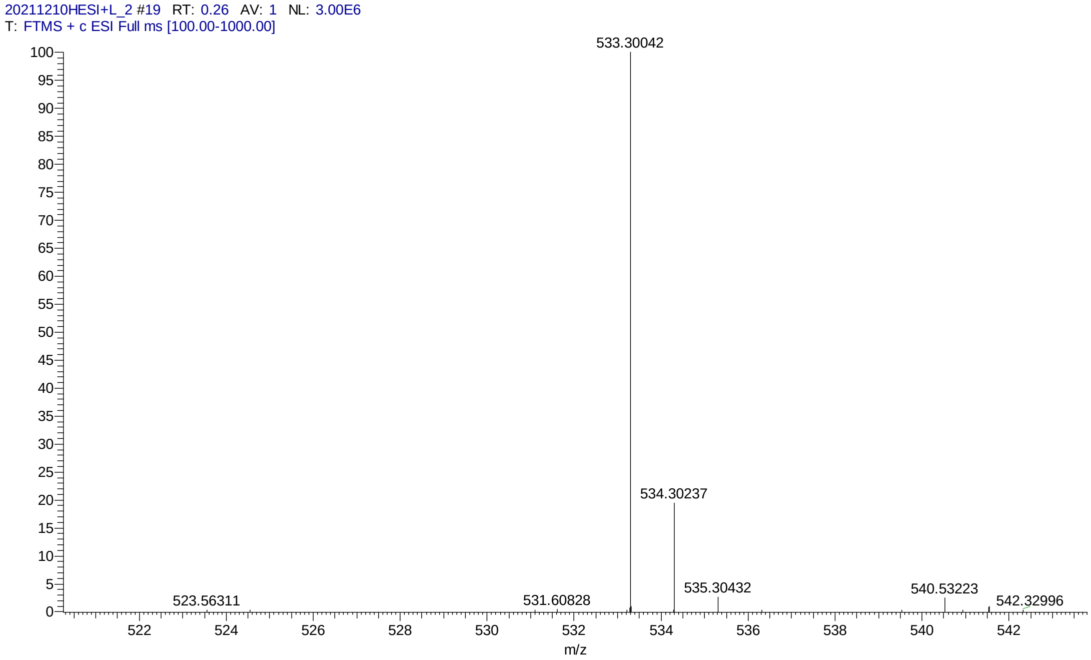

Figure S6. ESI-MS of the comonomer **L2**.

Compound(**L3**) : 3',3',5'-trimethyl-6-nitro-1'-(2-(undec-10-enoyloxy)ethyl)spiro[chromene-2,2'-indolin]-8-yl undec-10-enoate

DCC (15.1 g, 73.2 mmol, 2.8 equiv.) and 10-Undecenoic acid (12.5 g，70 mmol, 2.68 equiv.) were dissolved in dry DCM (100 mL) while stirring under N_2. DMAP (0.60 g，5.0 mmol, 0.19 equiv) in dry DCM (2 mL) and the compound **6a** (10.0 g，26.1 mmol, 1 equiv) in dry DCM (50 mL) were transferred to the reaction mixture. After stirring overnight, the crude product was isolated under reduced pressure using a rotary evaporator, which was purified by silica gel flash chromatography to yield **L3** as a blue-violet viscous liquid (10.6 g, 13.2 mmol, 50.6%).

^{1}H NMR (400 MHz, Chloroform-d) δ 7.92 (d, J = 2.6 Hz, 1H), 7.80 (d, J = 2.7 Hz, 1H), 6.92 (dd, J = 9.2, 4.0 Hz, 2H), 6.86 (d, J = 1.7 Hz, 1H), 6.52 (d, J = 7.9 Hz, 1H), 5.93 (d, J = 10.4 Hz, 1H), 5.80 (m, 2H), 5.00 (m, 2H), 4.93 (m, 2H), 4.20 (dt, J = 11.1, 6.2 Hz, 1H), 4.11 (dt, J = 11.1, 6.2 Hz, 1H), 3.27 (t, J = 6.2 Hz, 2H), 2.28 (s, 3H), 2.23 (t, J = 7.6 Hz, 2H), 2.04 (m, 6H), 1.54 (m, 2H), 1.34 (m,6H), 1.24 (m, 13H), 1.13 (m, 9H).

^{13}C NMR (101 MHz, Chloroform-d) δ 172.58, 170.04, 149.88, 143.40, 139.06, 138.11, 138.09, 136.71, 134.83, 128.11, 127.14, 126.82, 121.30, 120.50, 118.94, 118.15, 118.07, 113.21, 113.15, 106.56, 105.74, 61.39, 51.13, 41.51, 33.16, 32.79, 32.75, 32.50, 28.68, 28.34, 28.22, 28.17, 28.08, 28.06, 28.02, 27.91, 27.85, 27.84, 24.90, 23.80, 23.42, 19.95, 18.33.

ESI-MS (m/z): $[M+H]^+$ Calcd for C43 H59 O7 N2 , 715.43168; Found: 715.43158.

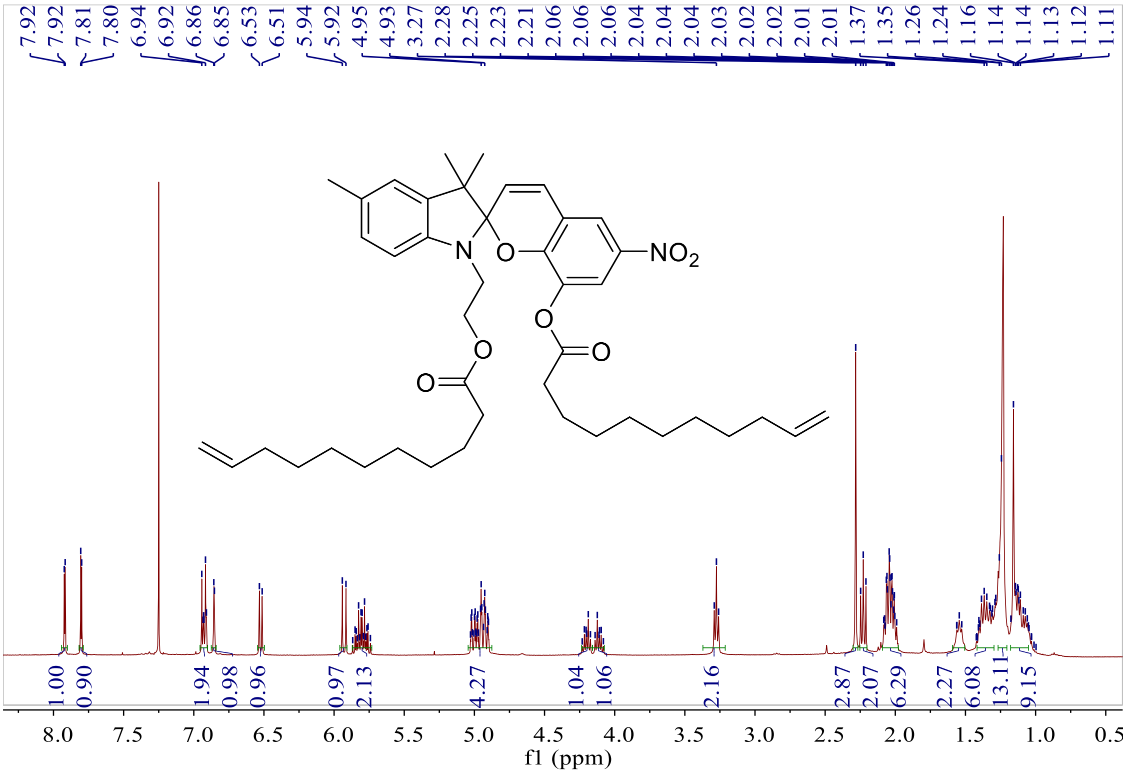

Figure S7. ^{1}H NMR spectrum of the comonomer **L3**.

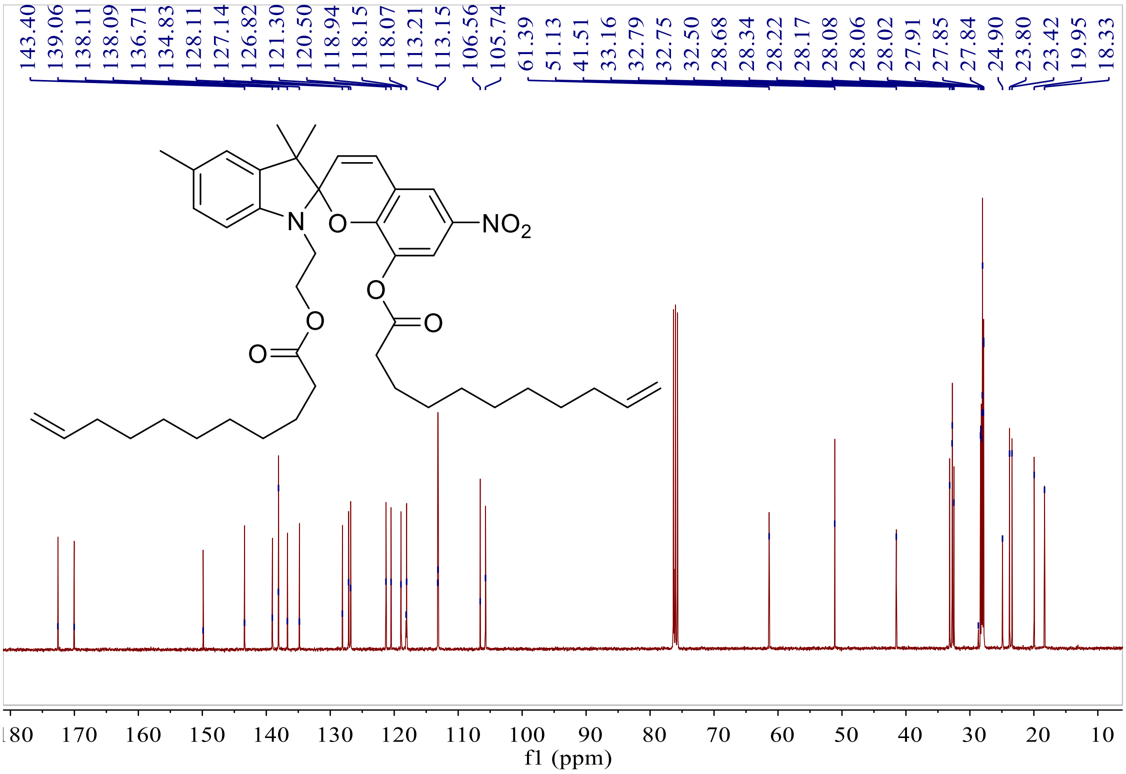

Figure S8. ^{13}C NMR spectrum of the comonomer **L3**.

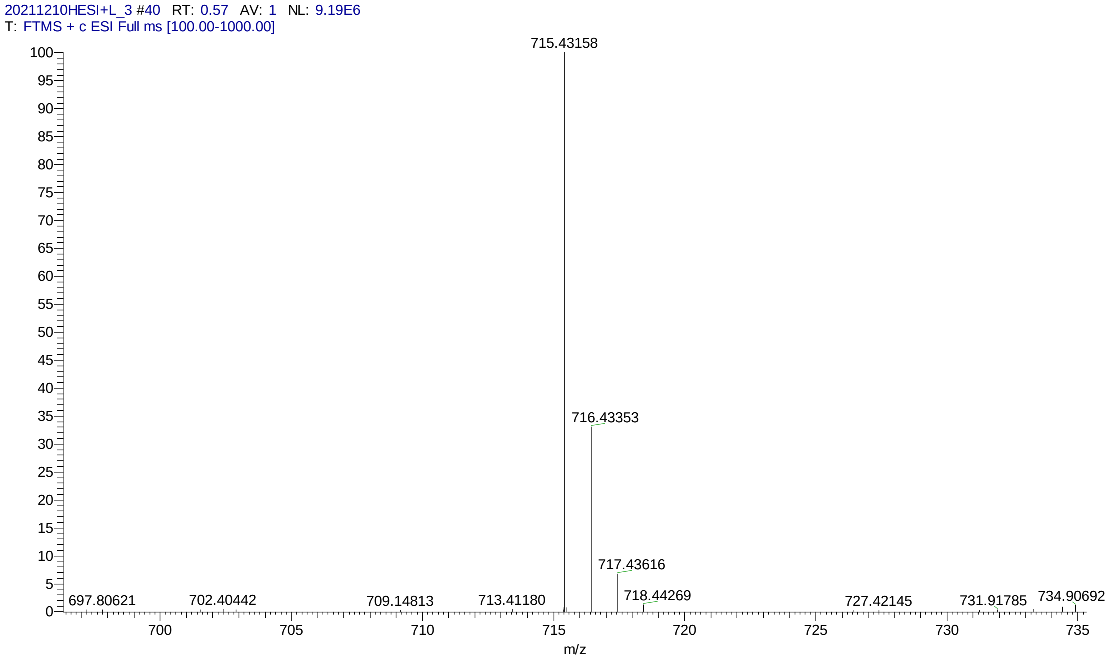

Figure S9. ESI-MS of the comonomer **L3**.

Compound (**L4**): 2-(3',3',5'-trimethyl-6-nitrospiro[chromene-2,2'-indolin]-1'-yl)ethyl (1S,2R,4S)-bicyclo[2.2.1]hept-5-ene-2-carboxylate

DCC (3.16 g，15.28 mmol, 1.4 equiv.) and 5-Norbornene-2-carboxylic acid (1.96 g，14.18 mmol, 1.3 equiv.) were dissolved in dry DCM (50 mL) while stirring under N_2. DMAP (0.12 g，21.0 mmol, 1.9 equiv.) in dry DCM (2 mL) and the compound **5a** (4.0 g，10.92 mmol, 1 equiv.) in dry DCM (20 mL) were transferred to the reaction mixture. After stirring overnight, the crude product was isolated under reduced pressure using a rotary evaporator, which was recrystallized with MeOH, filtered, and dried to yield **L4** as a yellow-green solid (4.0 g, 8.22 mmol, 75.3%).

^{1}H NMR (400 MHz, Chloroform-d) δ 8.01 (d, J = 8.5 Hz, 2H), 7.01 (d, J = 7.8 Hz, 1H), 6.92(m, 2H), 6.75 (d, J = 10.9 Hz, 1H), 6.61 (d, J = 7.9 Hz, 1H), 6.12 (m, 1H), 5.85 (m, 2H), 4.17 (m, 2H), 3.41 (m, 2H), 3.15 (d, J = 9.5 Hz, 1H), 2.87 (m, 2H), 2.33 (s, 3H), 1.88 (m, 1H), 1.39 (m, 2H), 1.27 (s, 4H), 1.17 (s, 3H).

^{13}C NMR (101 MHz, Chloroform-d) δ 174.67, 159.57 (d, J = 2.1 Hz), 144.60 (d, J = 3.8 Hz), 141.02, 137.88 (d, J = 9.2 Hz), 135.81 (d, J = 3.7 Hz), 132.27 (d, J = 11.8 Hz), 129.27, 128.47 – 127.97 (m), 125.96, 122.73 (d, J = 8.5 Hz), 121.98 (d, J = 3.4 Hz), 118.51, 115.56, 107.00 – 106.39 (m), 62.42 (d, J = 2.9 Hz), 52.88, 49.67, 46.37, 45.67 (d, J = 1.8 Hz), 43.30 (d, J = 1.6 Hz), 42.97 – 42.42 (m), 41.61, 29.31 (d, J = 7.2 Hz), 25.91, 21.00, 19.85.

ESI-MS (m/z): $[M+H]^+$ Calcd for C29 H31 O5 N2 , 487.2228; Found: 487.2228.

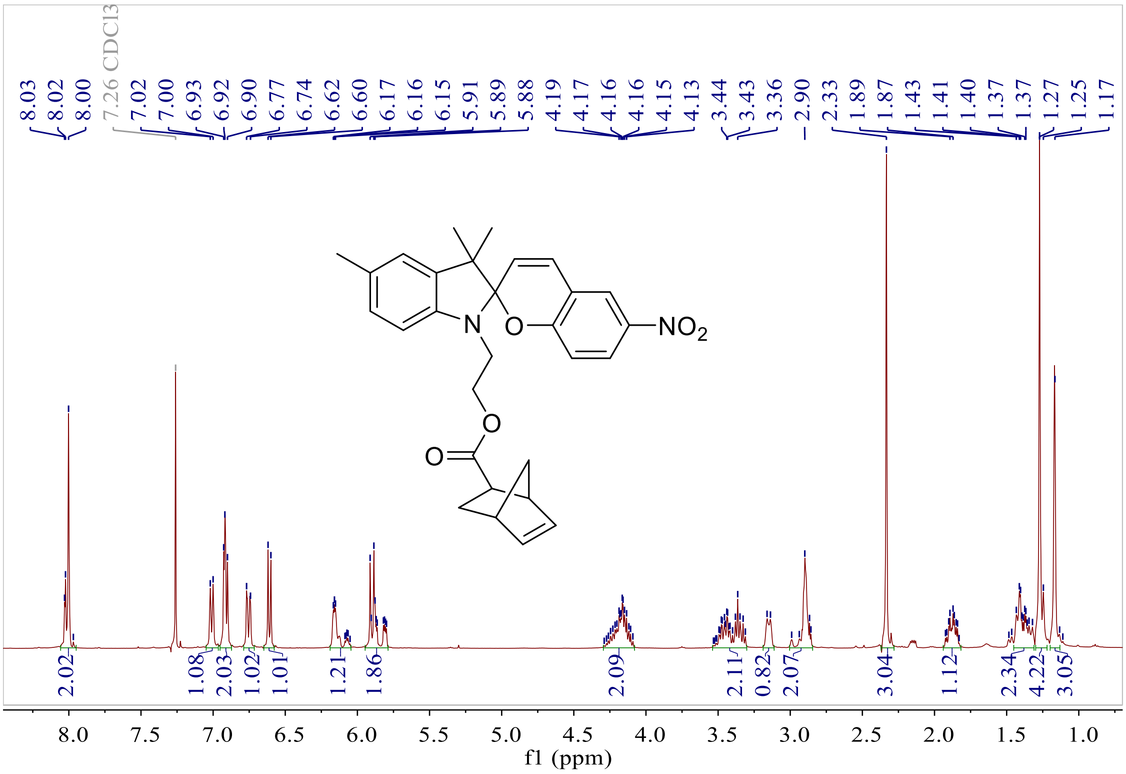

Figure S10. ^{1}H NMR spectrum of the comonomer **L4**.

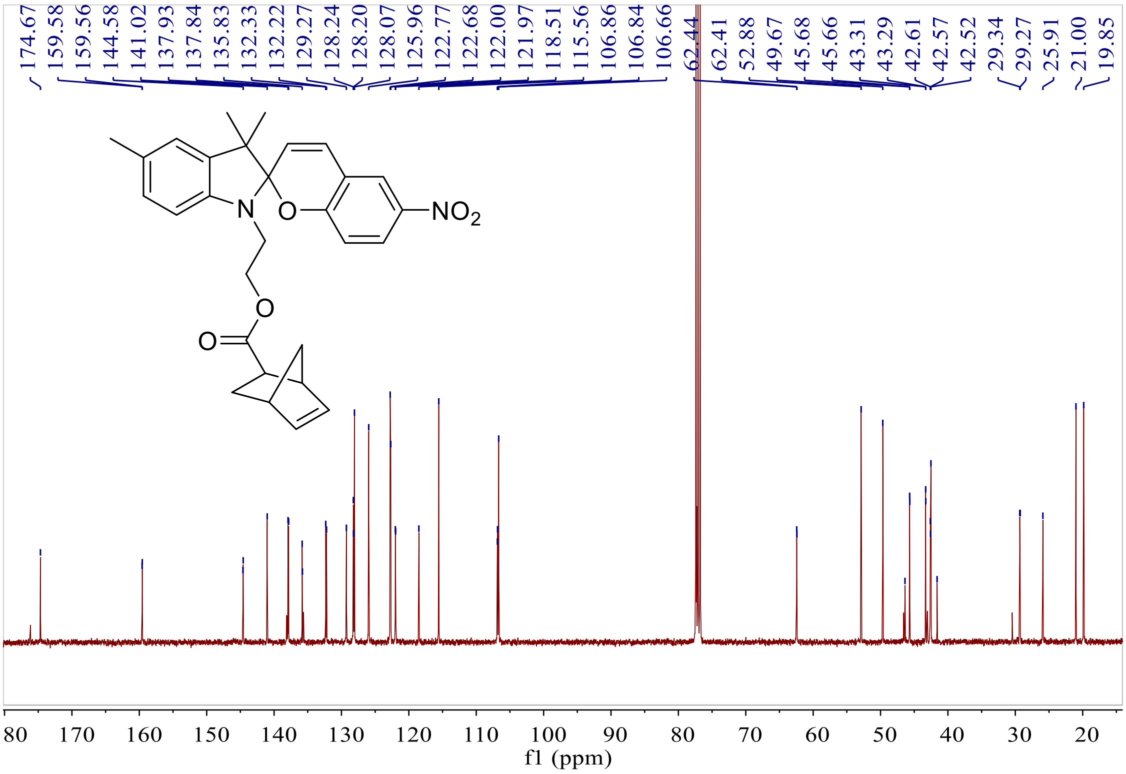

Figure S11. ^{13}C NMR spectrum of the comonomer **L4**.

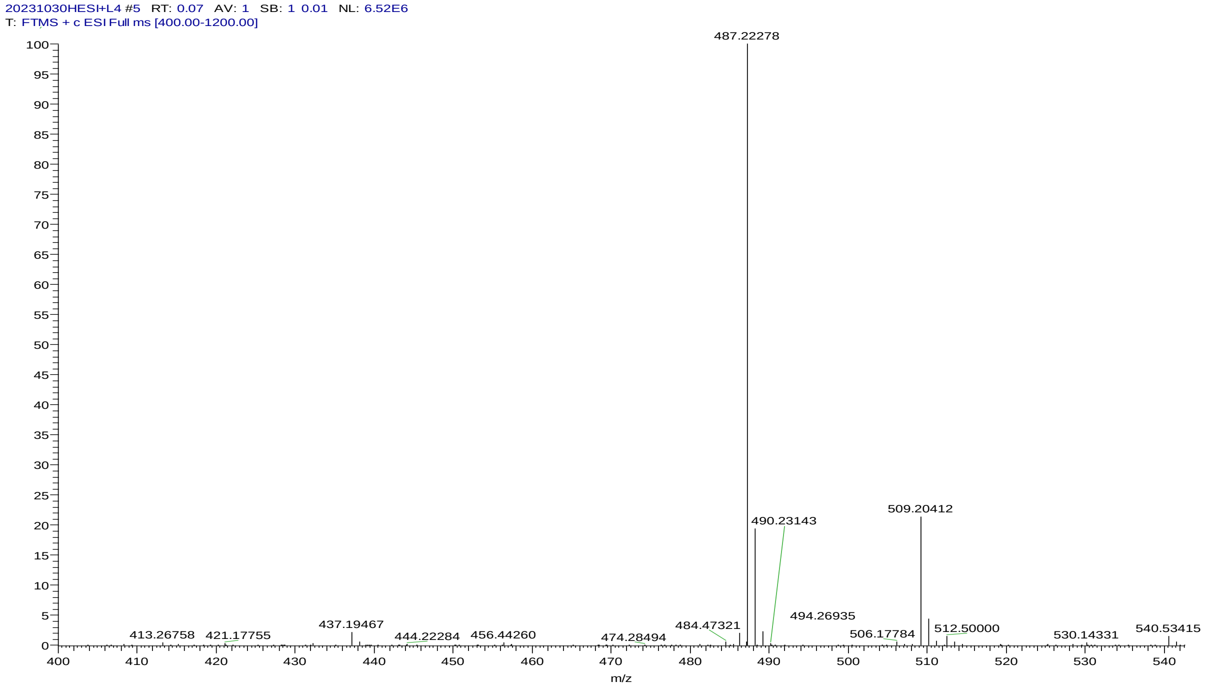

Figure S12. ESI-MS of the comonomer **L4**.

Compound (**L5**): 2-(3',3',5'-trimethyl-6-nitrospiro[chromene-2,2'-indolin]-1'-yl)ethyl methacrylate

Compound **5a** (6 g, 28.9 mmol, 1 equiv.) and DMAP (0.12 g，21.0 mmol, 2.4 equiv.) were dissolved in 200 ml THF, while stirring under N_2. Methacrylic anhydride (5 g, 41.7 mmol, 1.8 equiv.) was added to the mixture. After stirring overnight, the crude product was isolated under reduced pressure using a rotary evaporator, which was washed with HCl (1 mol/L). The mixture was recrystallized with MeOH, filtered, dried in vacuum to yield **L5** as a light brown solid (4.44 g, 20.8 mmol, 72%).

^{1}H NMR (400 MHz, Chloroform-d) δ 8.02 (d, J = 2.7 Hz, 1H), 7.99 (q, J = 2.6 Hz, 3H), 7.01 (d, J = 7.7 Hz, 0H), 6.92 – 6.86 (m, 2H), 6.75 (d, J = 8.7 Hz, 2H), 6.60 (d, J = 7.9 Hz, 2H), 6.07 (s, 0H), 5.86 (d, J = 10.3 Hz, 2H), 5.56 (t, J = 1.6 Hz, 2H), 4.29 (t, J = 6.3 Hz, 3H), 3.52 (dt, J = 15.1, 6.7 Hz, 2H), 3.39 (dt, J = 15.0, 5.9 Hz, 2H), 2.33 (s, 6H), 1.92 (s, 1H), 1.27 (s, 5H), 1.16 (s, 6H).

^{13}C NMR (101 MHz, Chloroform-d) δ 167.23, 159.52, 144.52, 141.02, 136.07, 135.85, 129.32, 128.23, 128.07, 125.97, 125.92, 122.78, 122.71, 121.88, 118.46, 115.56, 106.82, 106.59, 62.70, 52.83, 42.59, 25.89, 20.99, 19.83, 18.40.

ESI-MS (m/z): $[M+H]^+$ Calcd for C25 H27 O5 N2 , 435.19145; Found: 435.19147.

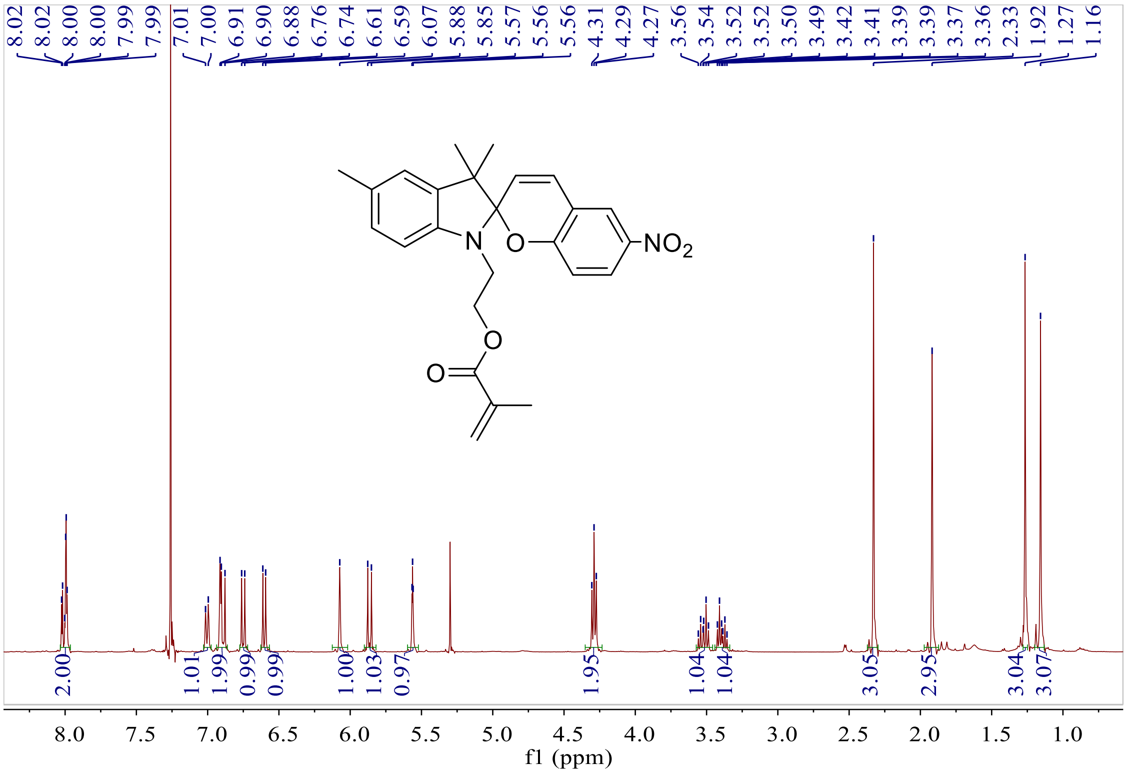

Figure S13. ^{1}H NMR spectrum of the comonomer **L5**.

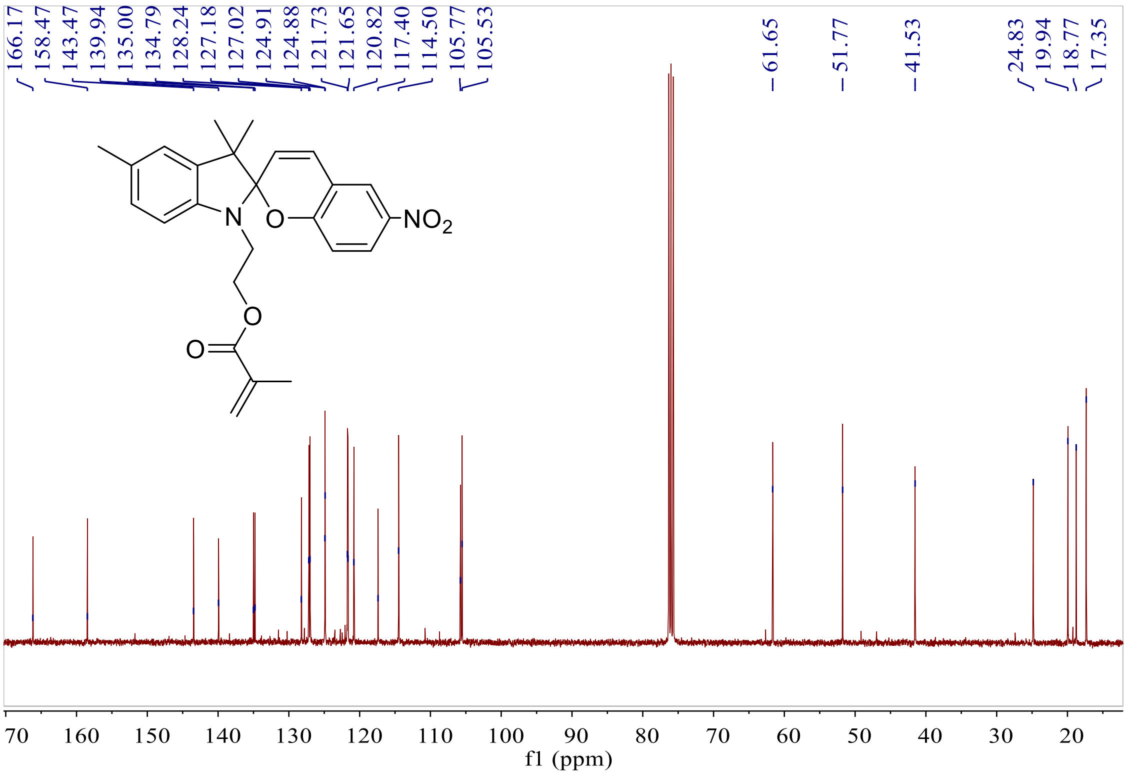

Figure S14. ^{13}C NMR spectrum of the comonomer **L5**.

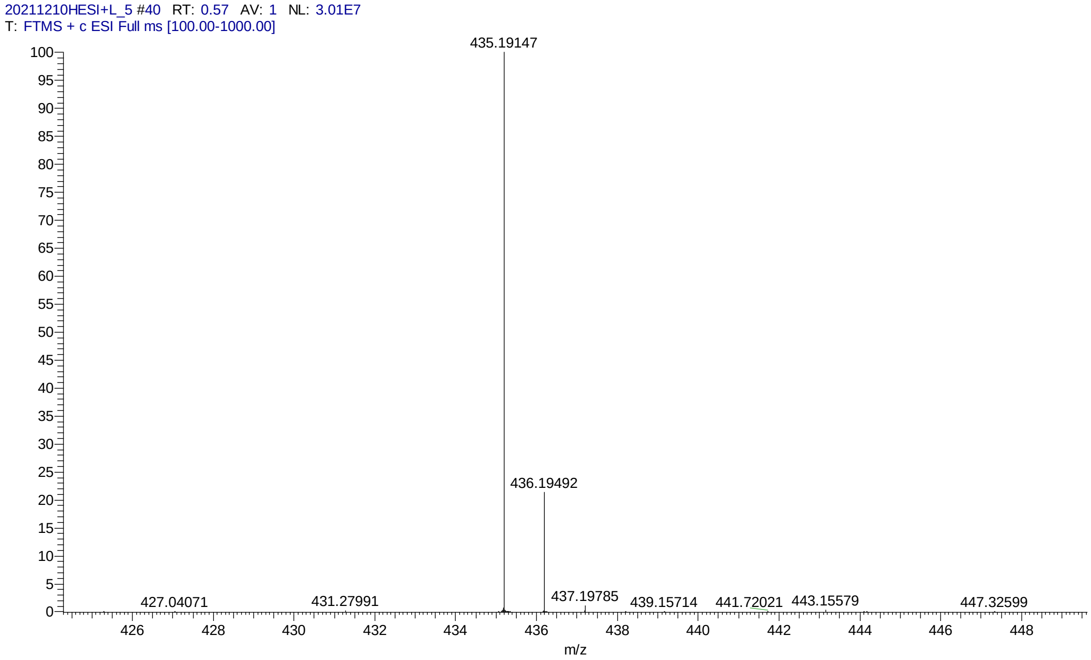

Figure S15. ESI-MS of the comonomer **L5**.

2.2. Procedure for polymerization

In a typical experiment, a 350 mL glass thick-walled pressure vessel was charged with toluene, a desired amount of comonomer, and a magnetic stir bar in the glovebox. The pressure vessel was connected to a high-pressure line, and the solution was degassed. The vessel was warmed to the desired temperature using an oil bath and allowed to equilibrate for 5 min. The metal catalyst **PO-Pd** in CH_2Cl_2 was injected into the polymerization system via syringe. With rapid stirring, the reactor was pressurized, maintained at a desired ethylene pressure, and stirred continuously for a desired period. The pressure vessel was vented, the polymerization was quenched via the addition of MeOH (5 mL), and the polymer was precipitated using excess MeOH. After filtration, the copolymer was washed with copious amounts of acetone until the color of the solution was clear. In the last, the copolymer was obtained and dried at 40 °C for 24 h under vacuum. The polar monomer incorporation (mol%) was calculated from NMR analysis.

2.3. Blending modification method

In order to obtain photochromic polymers by blending spiropyran monomer with bulk plastics (such as HDPE, PP, EPDM, LLDPE, etc), monomers and polymer matrix were processed in a twin-screw extruder (WLG-10, L/ D = 20). The polymer matrix was dried at 40 ℃ for 12 h before usage. The process conditions used in the extruder are as follows: feeding rate of 1 kg/h, screw speed of 70 rpm, processing time of 12 min, screw temperature

of 150 ℃ (for HDPE, EPDM and LLDPE) or 180 ℃ (for PP) and keep N_2 flowing. The blending experiments of each polymer matrix (100 phr) were carried out in two groups, one with 0.3 phr of DCP as additive, abbreviated to such as **HDPE-L5**, The addition amount of spiropyran monomer in each group was 5 phr.

2.4. The synthesis and NMR spectrum of PO-Pd catalyst

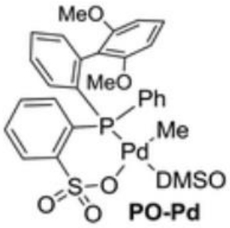

Preparation of **PO-Pd**. The phosphine sulfonate ligand[4](1.0 g, 2.0 mmol) was suspended in THF (16 mL). Pd(tmeda)Me_2 (550 mg, 2.1 mmol) was added at -5 ^{o}C. After stirring for 5 min, the evolution of gas stopped and the suspension turned clear. The solution was stirred overnight. The resulting white precipitate was filtered, washed with Et_2O and dried under reduced pressure to yield the tmeda-bridged dimer. The dimer was dispersed in 20 mL DMSO at room temperature. The solvent was removed under reduced pressure at 80 ^{o}C. The dimer complex is only slightly soluble in DMSO, therefore complete dissolution of the solid indicate complete conversion of the starting material. After removal of DMSO under reduced pressure, the resulting solid was dispersed in Et_2O, and isolated by filtration to yield a white solid (800 mg, 60%). ^{1}H NMR (CDCl3, 400 MHz): δ 8.31 (s, 1H), 7.60-7.55 (m, 3H), 7.43-7.29 (m, 8H), 7.24-7.22 (m, 2H), 3.73 (s, 3H, OMe), 2.85 (s, 6H, DMSO), 2.70 (s, 3H, OMe), 0.56 (s, 3H, Pd-Me). ^{13}C NMR (100 MHz, CDCl3): δ 158.3 (s), 157.9 (s), 149.1 (d, J_{PC} = 14 Hz), 142.5 (d, J_{PC} = 18 Hz), 136.3 (s), 135.1 (d, J_{PC} = 9 Hz), 134.7(s), 133.7 (d, J_{PC} = 10 Hz), 131.8 (s), 131.1 (s), 130.5 (d, J_{PC} = 7Hz), 129.0 (br), 128.9 (br), 128.8 (br), 128.7(br), 128.5 (br), 127.1 (d, J_{PC} = 8 Hz), 118.0 (s), 104.2 (s), 103.8 (s), 56.0 (s, OMe), 54.6 (s, OMe), 41.6 (br, DMSO), 4.1 (s, Pd-Me). ^{31}P NMR (DMSO-d6): δ 15.4. Anal. Calcd. for $C_{29}H_{31}O_6PPdS_2$: C, 51.44; H, 4.62. Found: C, 51.49; H, 4.60.

3. Results on multiple specimens are shown for reproducibility

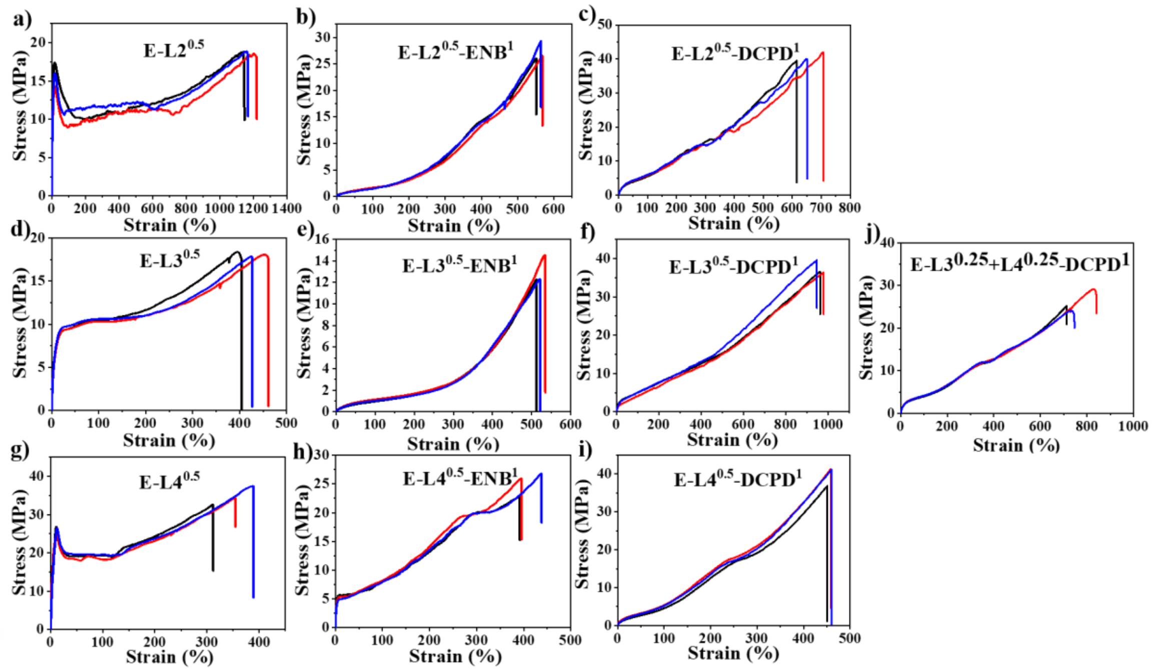

Figure S16. By stretching more than three times for different entries of samples, the repeatability is shown. The stretch splines are from a) Table 1, Entry 2; b) Table 1, Entry 4; c) Table 1, Entry 5; d) Table 1, Entry 6; e) Table 1, Entry 7; f) Table 1, Entry 8; g) Table 1, Entry 9; h) Table 1, Entry 10; i) Table 1, Entry 11. j) Table 1, Entry 12.

4. The cyclic tensile tests

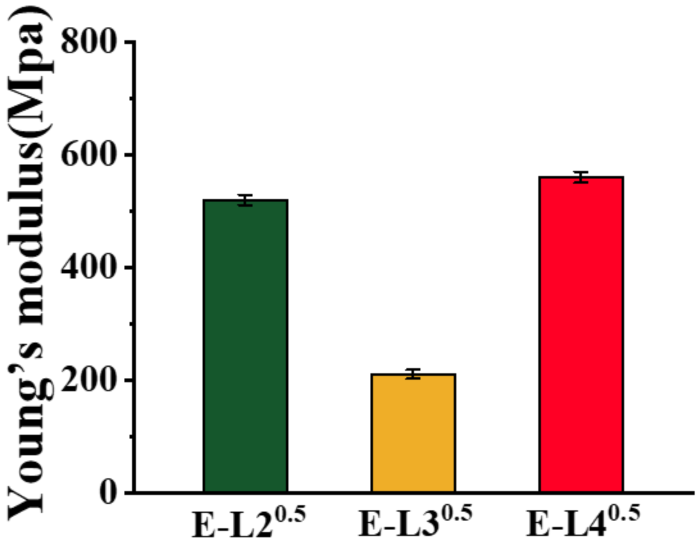

Figure S17. Young's modulus histogram of binary copolymers.

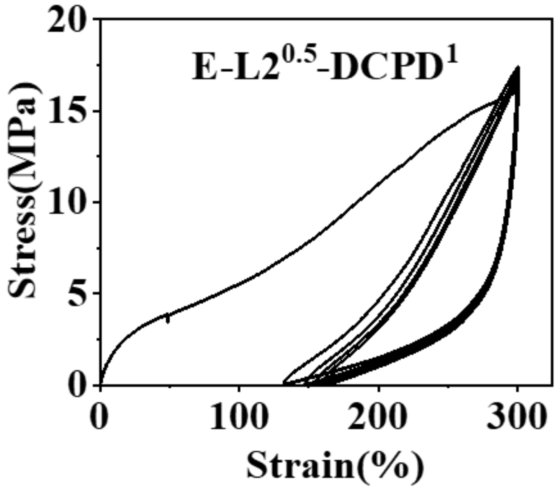

Figure S18. Plots of hysteresis experiments with ten cycles at a strain of 300 % for **E-L2$^{0.5}$-DCPD^{1}**.

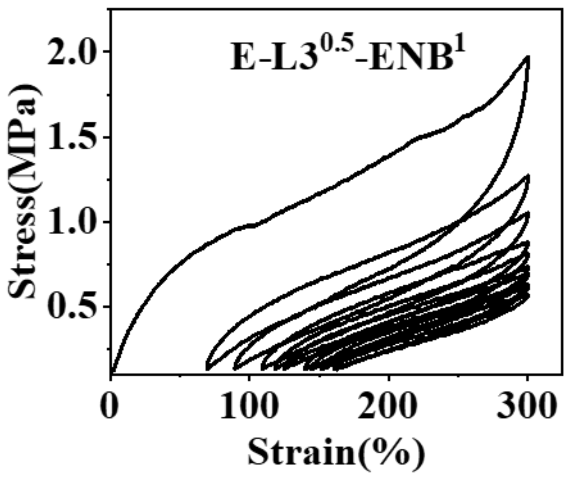

Figure S19. Plots of hysteresis experiments with ten cycles at a strain of 300 % for **E-L3$^{0.5}$-ENB^{1}**.

5. Water contact angle of the polymer samples

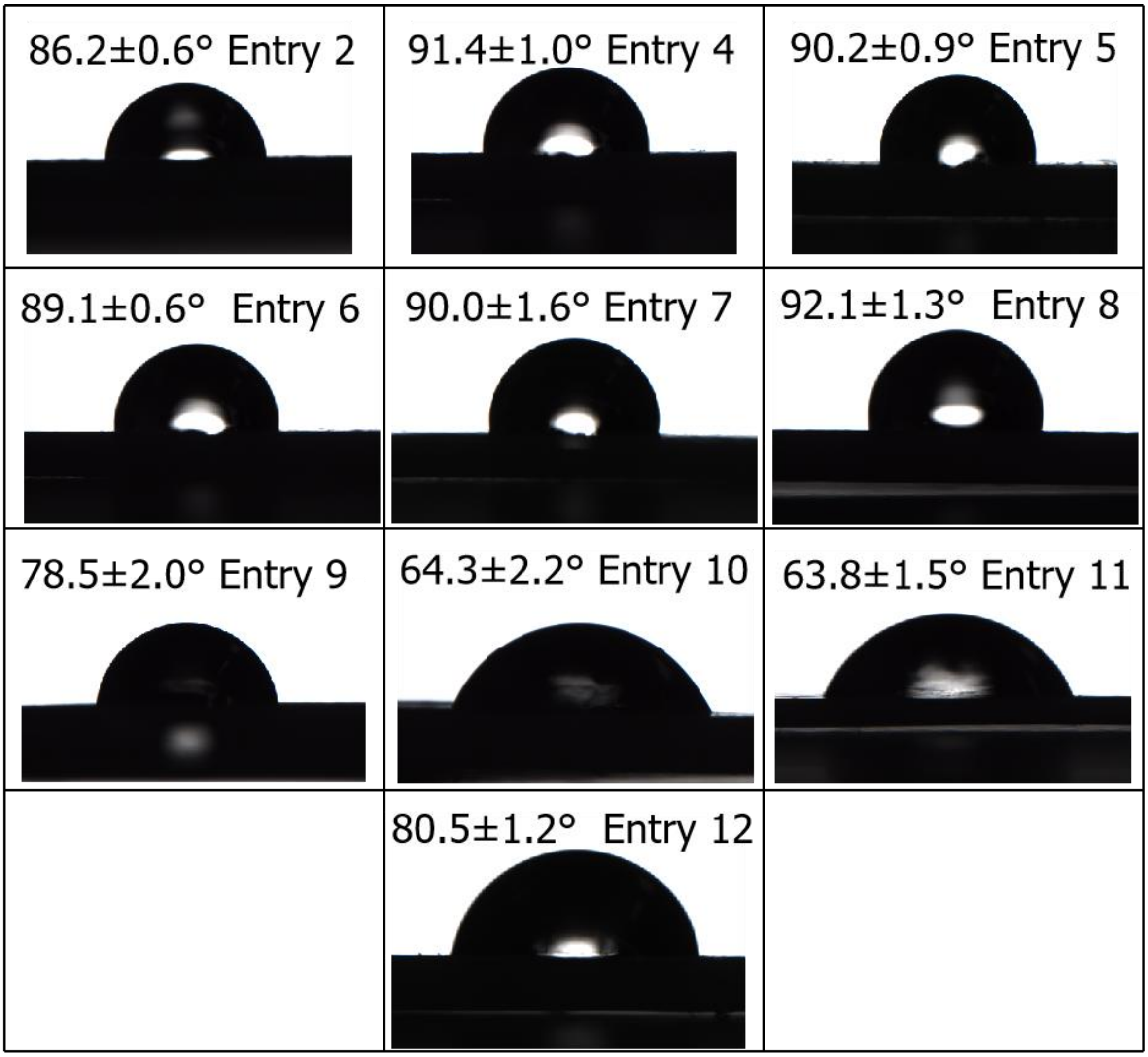

Figure S20. Water contact angle of the polymer samples from Table 1, Entries 2, 4-12.

6. Supplementary illustration

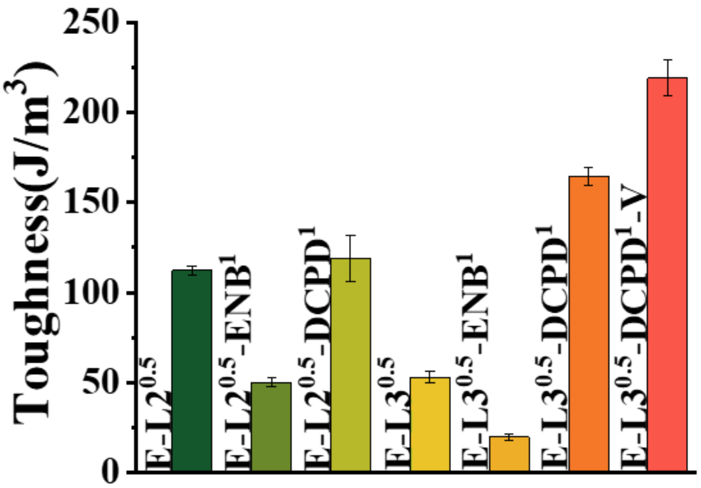

Figure S21. Toughness histogram of ternary copolymers. **E-L3$^{0.5}$-DCPD^{1}-V** represents the vulcanized **E-L3$^{0.5}$-DCPD^{1}**.

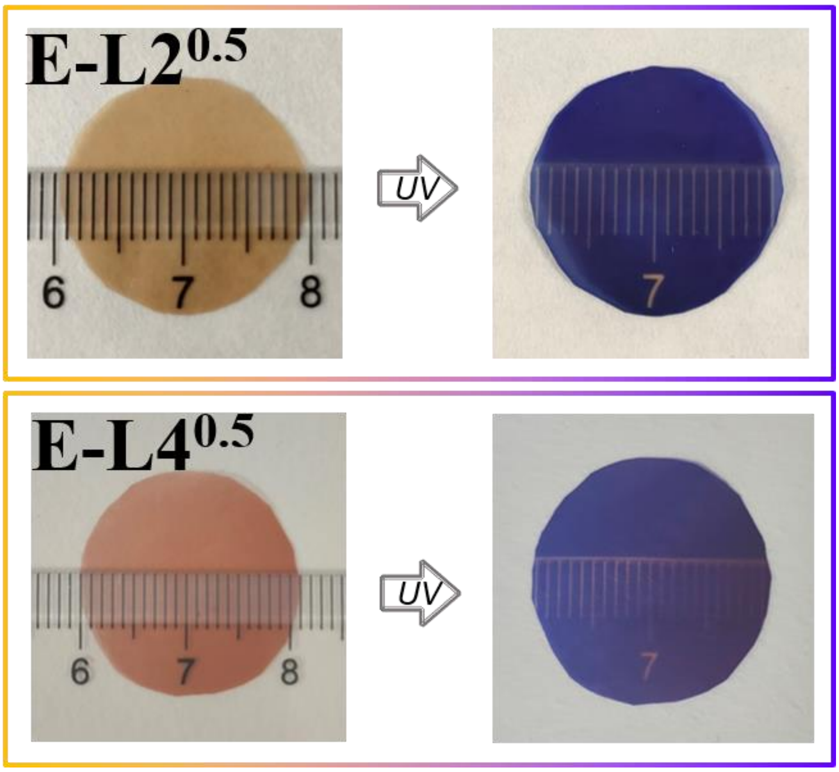

Figure S22. Photochromism of the binary copolymers of **E-L2$^{0.5}$** and **E-L4$^{0.5}$**.

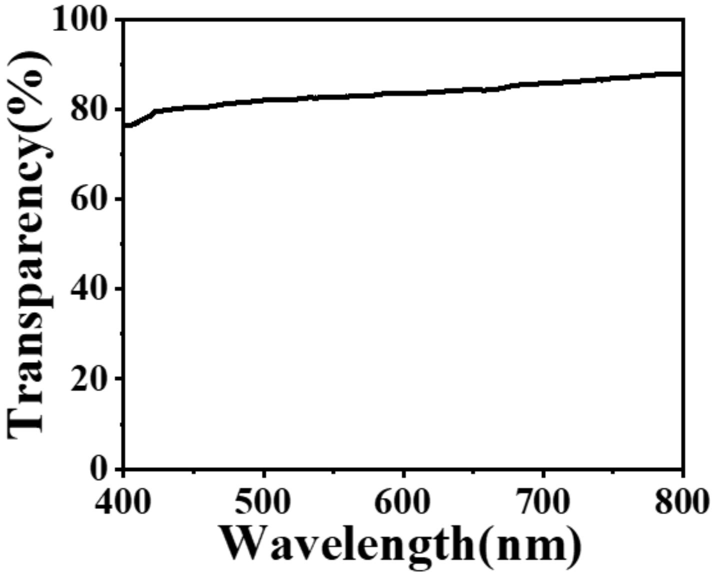

Figure S23. Transparency properties of **E-L2$^{0.5}$-DCPD^{1}**.

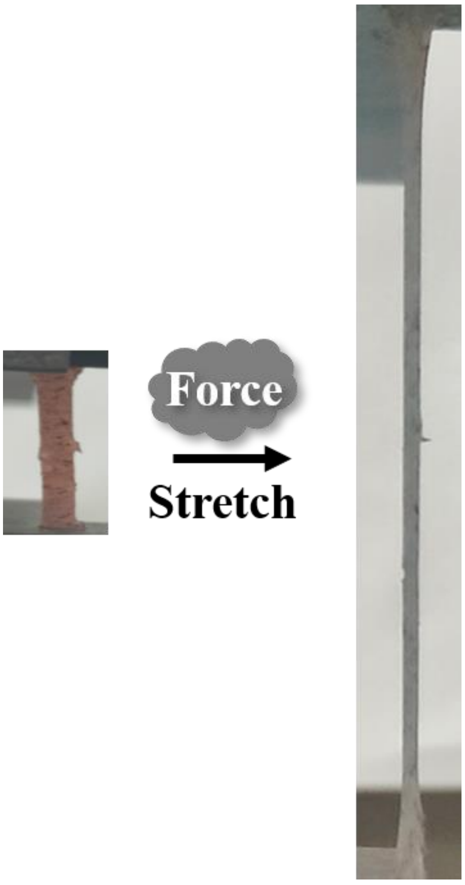

Figure S24. Color change of **E-L3$^{0.5}$-DCPD^{1}** after vulcanization (**E-L3$^{0.5}$-DCPD^{1}-V**) during stretching.

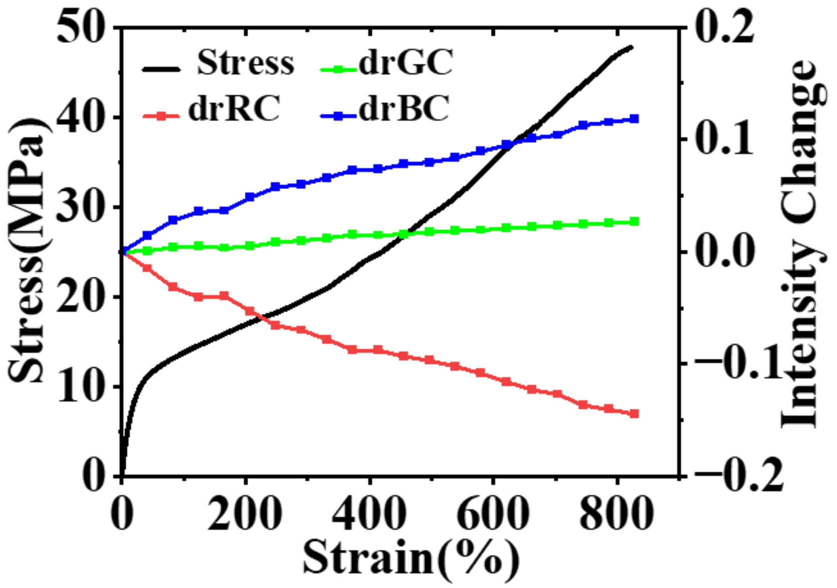

Figure S25. Stress, change of intensity in drRC, drGC and drBC as a function of strain while the stretching process of **E-L3$^{0.5}$-DCPD^{1}** after vulcanization (**E-L3$^{0.5}$-DCPD^{1}-V**).

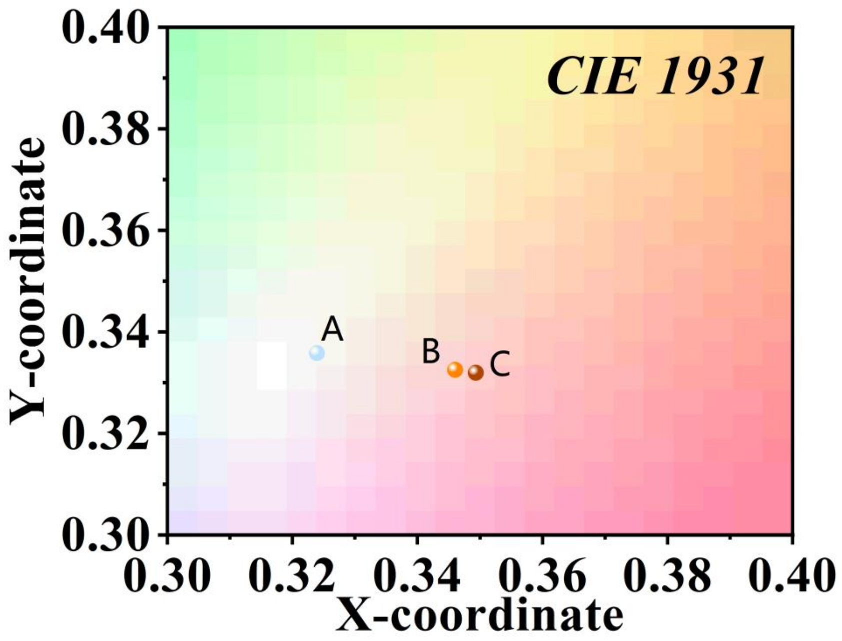

Figure S26. This picture is a local magnification of Figure 4B, in which point C is the initial chromaticity coordinate of the sample, point A is the chromaticity coordinate after stretching, and point B is the chromaticity coordinate of the sample after mechanical, optical and thermal stimulation.

7. Shape memory properties of functionalized polyolefins

Strain recovery ratio (R_r) is critical parameter to describe the shape memory effect of the SMP samples

$$R_r = \frac{\theta_{fix} - \theta_{rec}}{\theta_{fix}} \times 100\%$$

Where θ_{fix} represents the angle of the spline after the temporary shape is fixed, and θ_{rec} represents the angle of the spline after the shape is restored. Taking E-L$2^{0.5}$-DCPD^{1} as an example, the schematic diagram of the whole process is shown in Figure S27.

Thermo-induced shape memory: E-L$2^{0.5}$-DCPD^{1} was shaped into round films with a diameter of 2 cm and a thickness of 0.2 mm at a temperature above T_m (90 °C), were folded in half at 50 °C, record θ_{fix} at this time. Then cooled to room temperature. Its original shape was restored by thermal stimulation (60 °C), record the θ_{rec} at regular intervals. Calculate the recovery rate corresponding to different recovery time, and the result is shown in Figure 10 (blue Pentagon).

Photoinduced shape memory: E-L$3^{0.25}$+L$4^{0.25}$-DCPD^{1} was shaped into round films with a diameter of 2 cm and a thickness of 0.2 mm at a temperature above T_m (90 °C), were folded in half at 50 °C, record θ_{fix} at this time. Then cooled to room temperature. Its original shape was restored by UV light (25 °C), record the θ_{rec} at regular intervals. Calculate the recovery rate corresponding to different recovery time, and the result is shown in Figure 10 (red triangle).

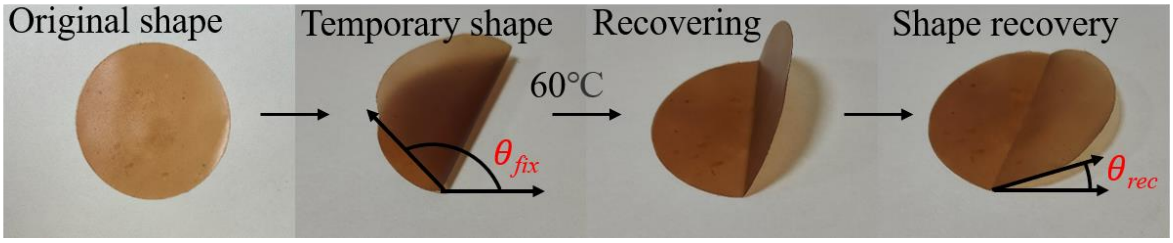

Figure S27. Schematic diagram of strain recovery ratio test.

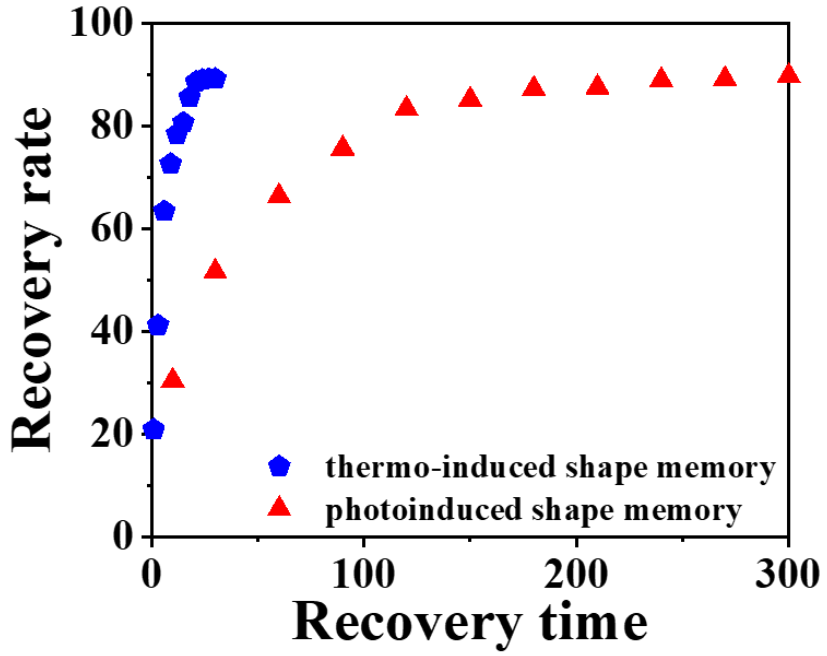

Figure S28. The curve between deformation recovery rate and recovery time of thermo-induced shape memory and photoinduced shape memory.

8. Photochromic ability of blended samples

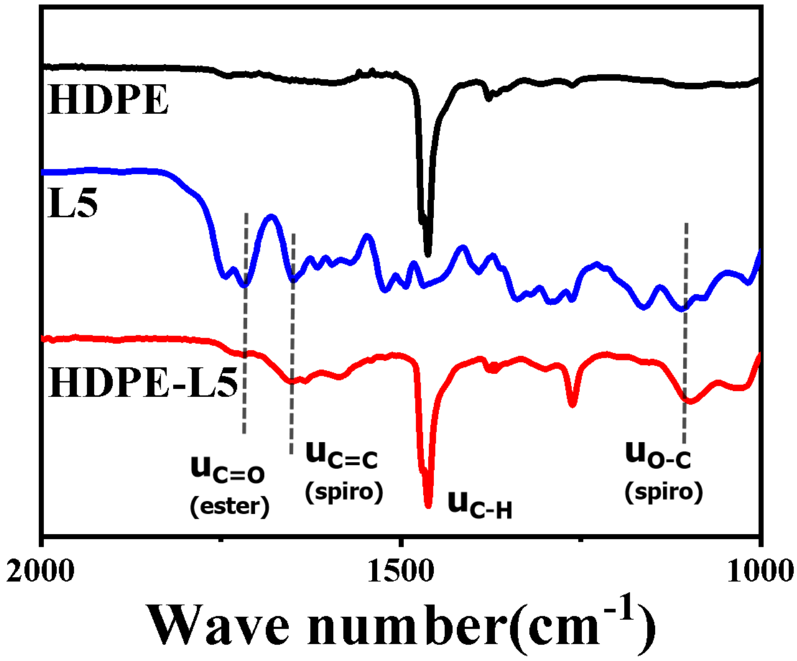

Figure S29. FTIR characterization of monomers and blended polymers. Black, blue and red lines show the infrared spectrum of HDPE, L5 and blend samples respectively at the selected wavelength.

9. The relationship between the introduction of spiropyran-based monomers and the variation of UV absorption

Table S1. Ethylene copolymerization studies with PO-Pd.

**Ent.**[a)]	**Comonomer 1**	**Comonomer 2**	**Yield/ g**	**Act.**[b)]**/ 10^5**	**X_{mol}%**[c)]	**Absorption variation at 572 nm**[d)]
1	**L2**(0.1M)	-	2.19	2.2	0.03	0.119
2	**L2**(0.3M)	-	2.03	2.0	0.1	0.131
3	**L2**(0.5M)	-	1.80	1.8	0.6	0.197
4	**L2**(0.7M)	-	1.6	1.6	0.8	0.215
5	**L2**(0.9M)	-	1.58	1.6	1.4	0.223

[a)]Conditions: PO-Pd 10 μmol; 20 mL Tol; *P* = 8 atm; *t* = 1 h; *T* = 80 °C; Monomer concentration, mol L^{-1}; [b)] Activity = 10^5 $g \cdot mol^{-1} \cdot h^{-1}$; [c)] Monomer incorporation ratio, determined by ^{1}H NMR; [d)]The variation of ultraviolet absorption Spectrum before and after ultraviolet irradiation for 30 s at 572 nm.

With the increase of spiropyran incorporations, the discoloration response strength of polymer increases. The relevant results can be seen in Figure S30.

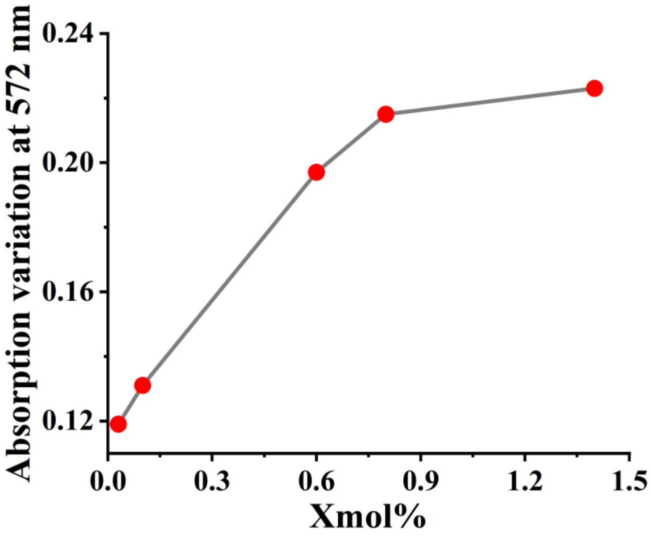

Figure S30. Schematic diagram of the relationship between monomer insertion ratio and absorption variation at 572 nm.

Table S2. Reactive extrusion of LDPE by adding L5 monomer in a twin-screw extruder.

Ent.	Monomer	Additive.	Time/min	T/°C	Absorption variation at 572nm[a)]
1	**L5**(1phr)	DCP(0.3phr)	12	150	0.106
2	**L5**(0.5phr)	DCP(0.3phr)	12	150	0.082
3	**L5**(0.1phr)	DCP(0.3phr)	12	150	~0

[a)]The variation of ultraviolet absorption Spectrum before and after ultraviolet irradiation for 30 s at 572 nm.

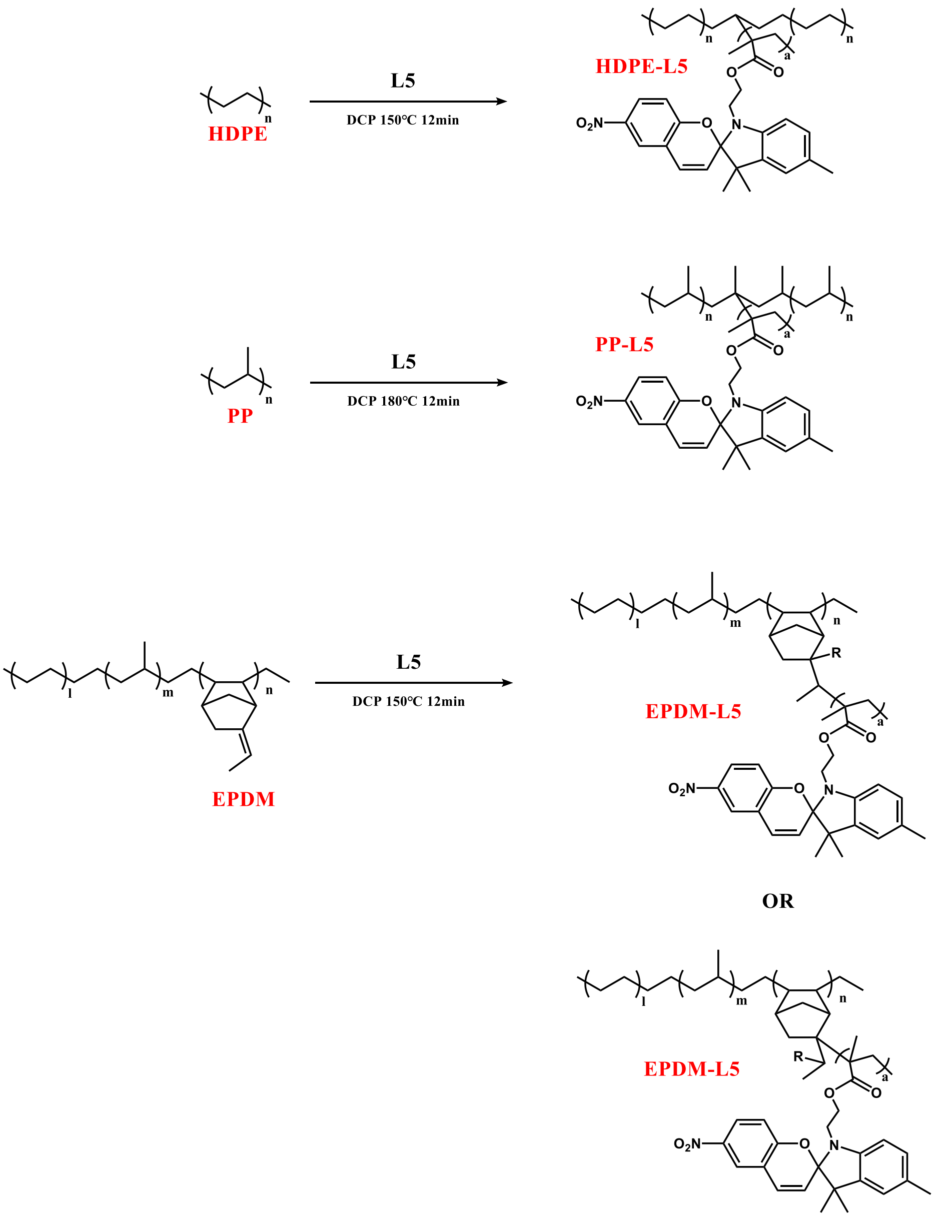

Figure S31. Chemical composition of polymer achieved by reactive extrusion.

With LDPE as the bulk material, by continuously reducing the content of L5 in the blend system, we found that the minimum comonomer addition amount for achieving the photo-response is between 0.1phr and 0.5phr, and the discoloration progress is shown in Table S2 and Figure S32.

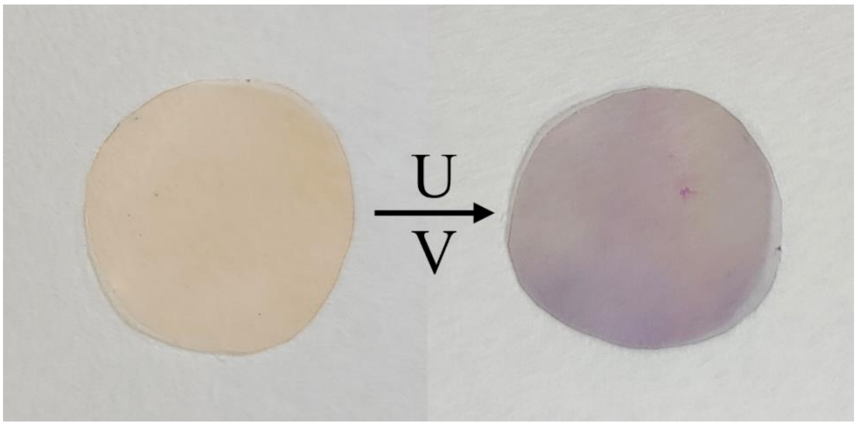

Figure S32. Color change diagram of blended samples before and after ultraviolet irradiation for 30 seconds. (The blend system contains 99.5phr LDPE and 0.5phr L5).

10. ¹H NMR and ¹³C NMR of copolymer

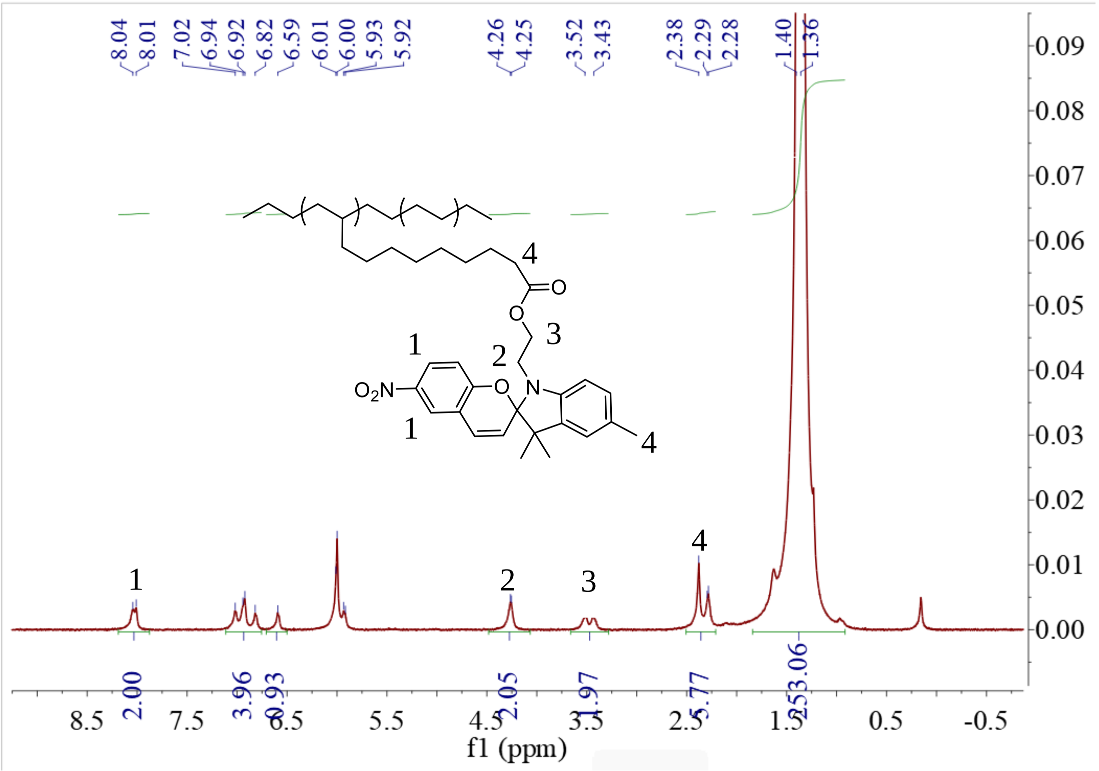

Figure S33. ¹H NMR spectrum of the copolymer from Table 1, Entry 3. ($C_2D_2Cl_4$, 120 ^oC).

$$\mathrm{Incorp(L2)\%} = \frac{\frac{I_2}{2}}{\frac{I_2}{2} + \frac{I_{CH_2+CH_3} - \frac{I_2}{2} \times 23}{4}} \times 100\%$$

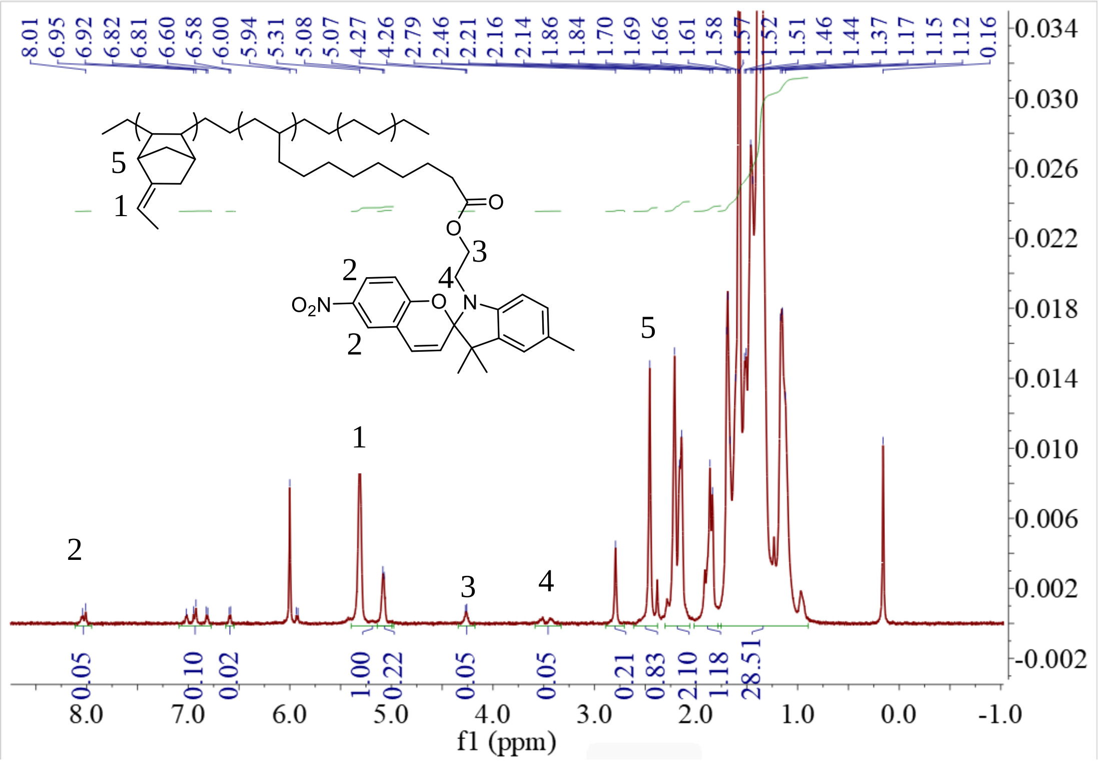

Figure S34. ^{1}H NMR spectrum of the copolymer from Table 1, Entry 4. ($C_2D_2Cl_4$, 120 °C).

$$\mathrm{Incorp(ENB)\%} = \frac{I_1}{I_1 + \frac{I_{CH_2+CH_3} - 6 \times I_1 - \frac{I_3 \times 23}{2I_1}}{4} + \frac{I_3}{2I_1}} \times 100\%$$

$$\mathrm{Incorp(L2)\%} = \frac{\frac{I_3}{2I_1}}{I_1 + \frac{I_{CH_2+CH_3} - 6 \times I_1 - \frac{I_3 \times 23}{2I_1}}{4} + \frac{I_3}{2I_1}} \times 100\%$$

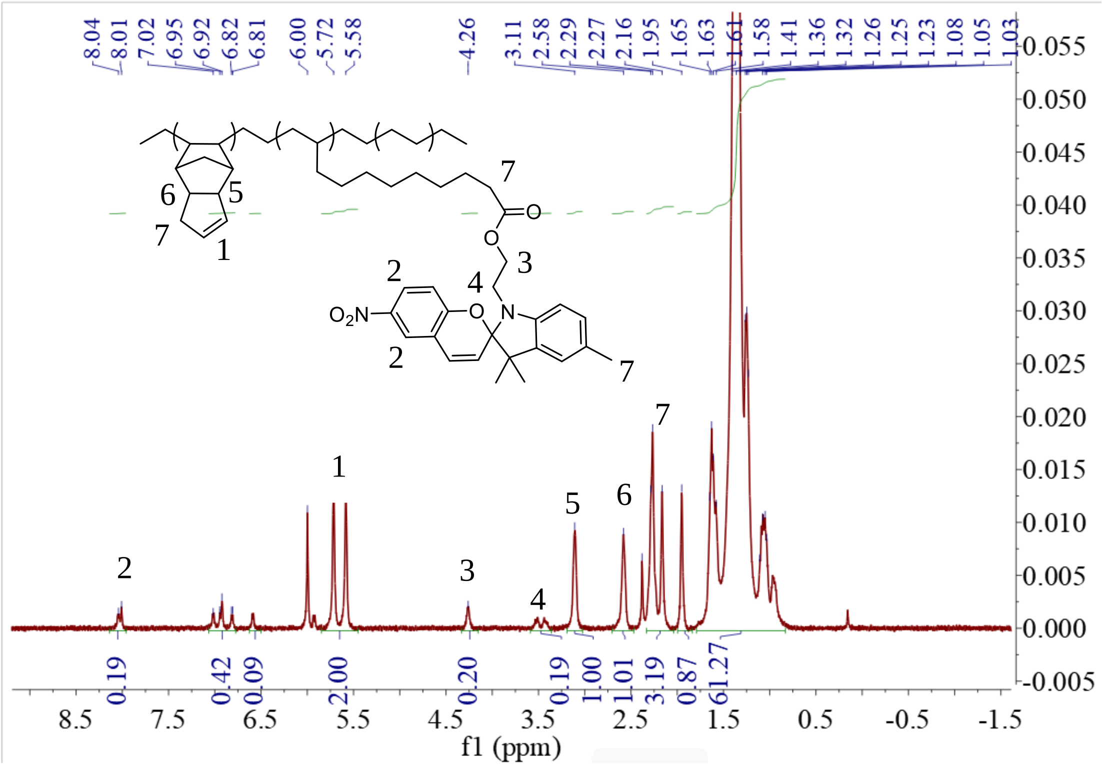

Figure S35. ^{1}H NMR spectrum of the copolymer from Table 1, Entry 5. ($C_2D_2Cl_4$, 120 °C).

$$\mathrm{Incorp(DCPD)\%} = \frac{\frac{I_1}{2}}{\frac{I_1}{2} + \frac{I_{CH_2+CH_3} - 4 \times \frac{I_1}{2} - \frac{I_3 \times 23}{I_1}}{4} + \frac{I_3}{I_1}} \times 100\%$$

$$\mathrm{Incorp(L2)\%} = \frac{\frac{I_3}{I_1}}{\frac{I_1}{2} + \frac{I_{CH_2+CH_3} - 4 \times \frac{I_1}{2} - \frac{I_3 \times 23}{I_1}}{4} + \frac{I_3}{I_1}} \times 100\%$$

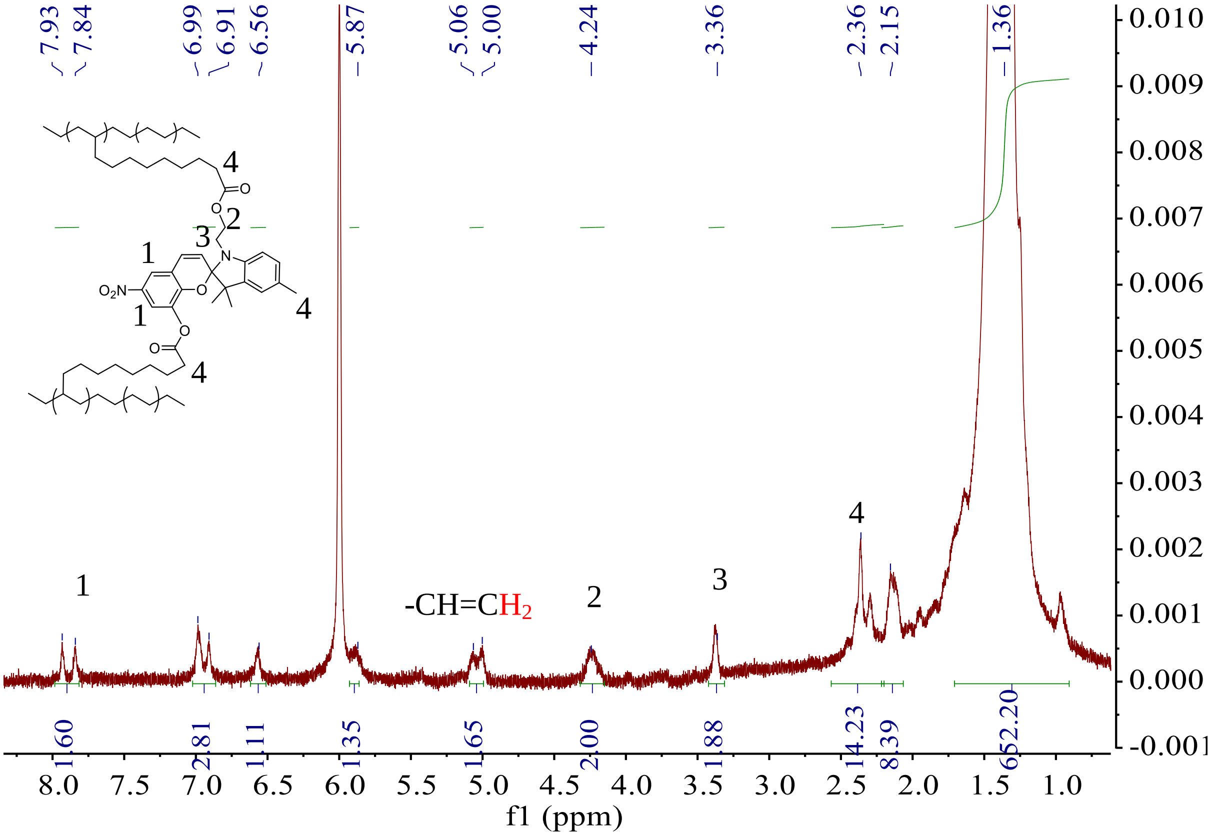

Figure S36. ^{1}H NMR spectrum of the copolymer from Table 1, Entry 6. ($C_2D_2Cl_4$, 120 °C).

$$\text{Both side(L3)\%} = \frac{I_3 - I_{-CH=CH2}}{I_3} \times 100\% = 17.5\%$$

$$\text{Incorp(L3)\%} = \frac{\frac{I_2}{2}}{\frac{I_2}{2} + \frac{I_{CH_2+CH_3} - \frac{I_2}{2} \times 40 \times 0.175 - \frac{I_2}{2} \times 35 \times 0.825}{4}} \times 100\%$$

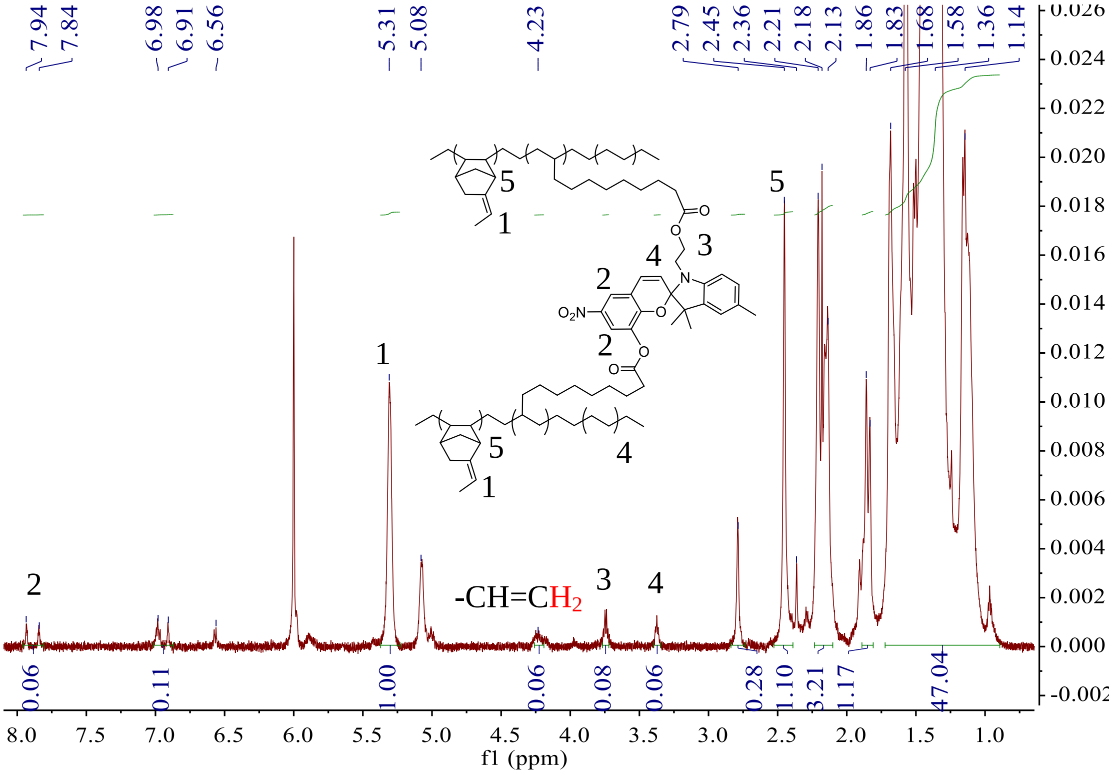

Figure S37. ^{1}H NMR spectrum of the copolymer from Table 1, Entry 7. ($C_2D_2Cl_4$, 120 ^{o}C).

$$\text{Both side(L3)\%} = \frac{I_3 - I_{-CH=CH2}}{I_3} \times 100\% = 25\%$$

$$\text{Incorp(ENB)\%} = \frac{I_1}{I_1 + \frac{I_{CH_2+CH_3} - 6 \times I_1 - \frac{I_3 \times 40}{2I_1} \times 0.25 - \frac{I_3 \times 35}{2I_1} \times 0.75}{4} + \frac{I_3}{2I_1}} \times 100\%$$

$$\text{Incorp(L3)\%} = \frac{\frac{I_3}{2I_1}}{I_1 + \frac{I_{CH_2+CH_3} - 6 \times I_1 - \frac{I_3 \times 40}{2I_1} \times 0.25 - \frac{I_3 \times 35}{2I_1} \times 0.75}{4} + \frac{I_3}{2I_1}} \times 100\%$$

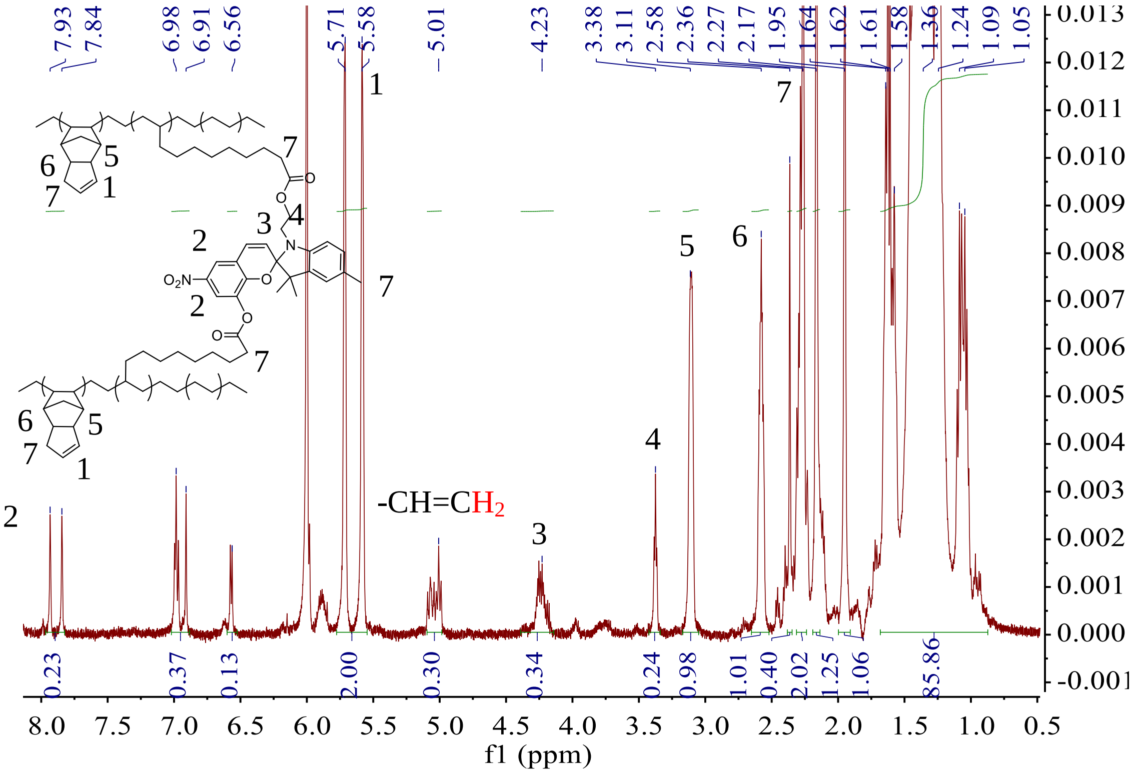

Figure S38. ^{1}H NMR spectrum of the copolymer from Table 1, Entry 8. ($C_2D_2Cl_4$, 120 ^{o}C).

$$\text{Both side(L3)}\% = \frac{I_3 - I_{-CH=CH2}}{I_3} \times 100\% = 11.8\%$$

$$\text{Incorp(DCPD)}\% = \frac{\frac{I_1}{2}}{\frac{I_1}{2} + \frac{I_{CH_2+CH_3} - 4\times\frac{I_1}{2} - \frac{I_3\times 40}{2I_1}\times 0.118 - \frac{I_3\times 35}{2I_1}\times 0.882}{4} + \frac{I_3}{I_1}} \times 100\%$$

$$\text{Incorp(L3)}\% = \frac{\frac{I_3}{I_1}}{\frac{I_1}{2} + \frac{I_{CH_2+CH_3} - 4\times\frac{I_1}{2} - \frac{I_3\times 40}{2I_1}\times 0.118 - \frac{I_3\times 35}{2I_1}\times 0.882}{4} + \frac{I_3}{I_1}} \times 100\%$$

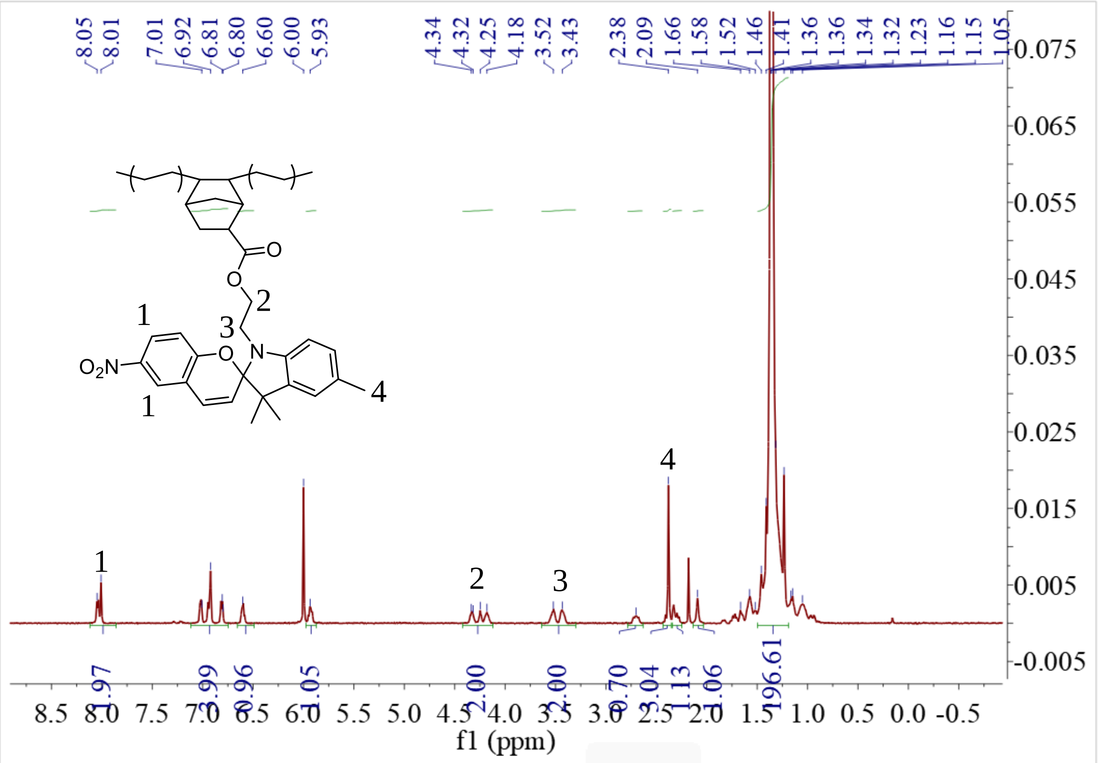

Figure S39. ^{1}H NMR spectrum of the copolymer from Table 1, Entry 9. ($C_2D_2Cl_4$, 120 ^{o}C).

$$\text{Incorp(L4)\%} = \frac{\frac{I_2}{2}}{\frac{I_2}{2} + \frac{I_{CH_2+CH_3} - \frac{I_2 \times 8}{2}}{4}} \times 100\%$$

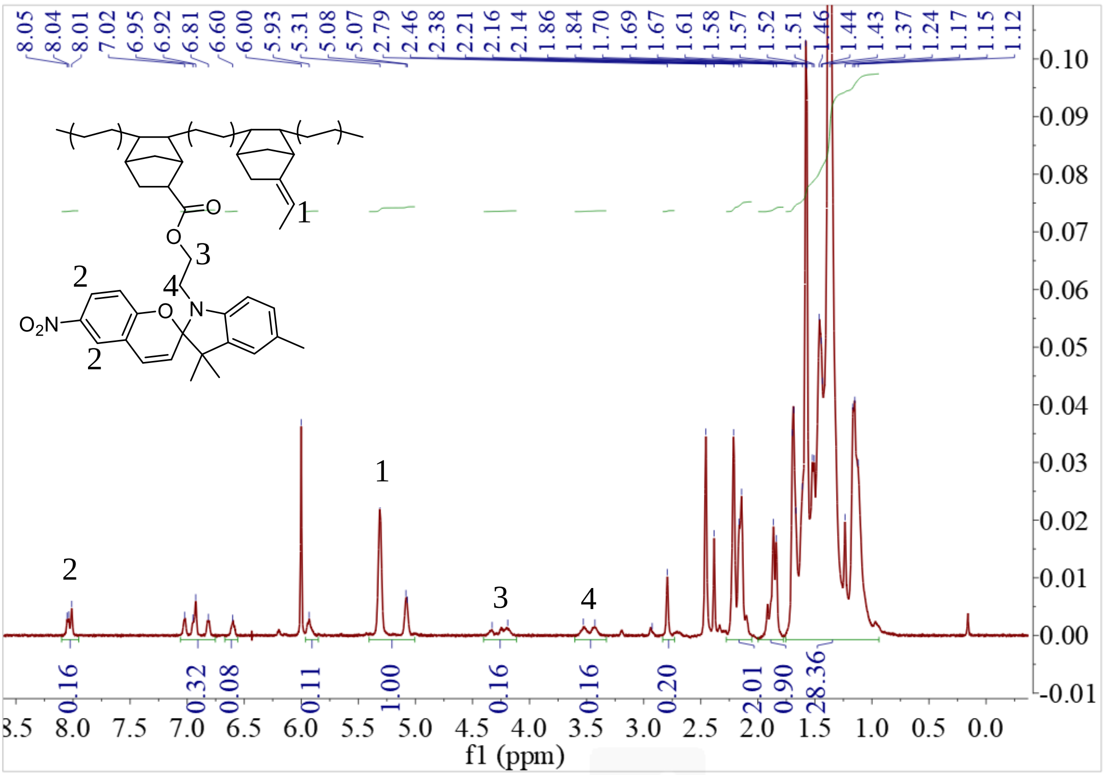

Figure S40. ^{1}H NMR spectrum of the copolymer from Table 1, Entry 10. ($C_2D_2Cl_4$, 120 ^oC).

$$\text{Incorp(ENB)\%} = \frac{I_1}{I_1 + \frac{I_{CH_2+CH_3} - 6 \times I_1 - \frac{I_3 \times 8}{2I_1}}{4} + \frac{I_3}{2I_1}} \times 100\%$$

$$\text{Incorp(L4)\%} = \frac{\frac{I_3}{2I_1}}{I_1 + \frac{I_{CH_2+CH_3} - 6 \times I_1 - \frac{I_3 \times 8}{2I_1}}{4} + \frac{I_3}{2I_1}} \times 100\%$$

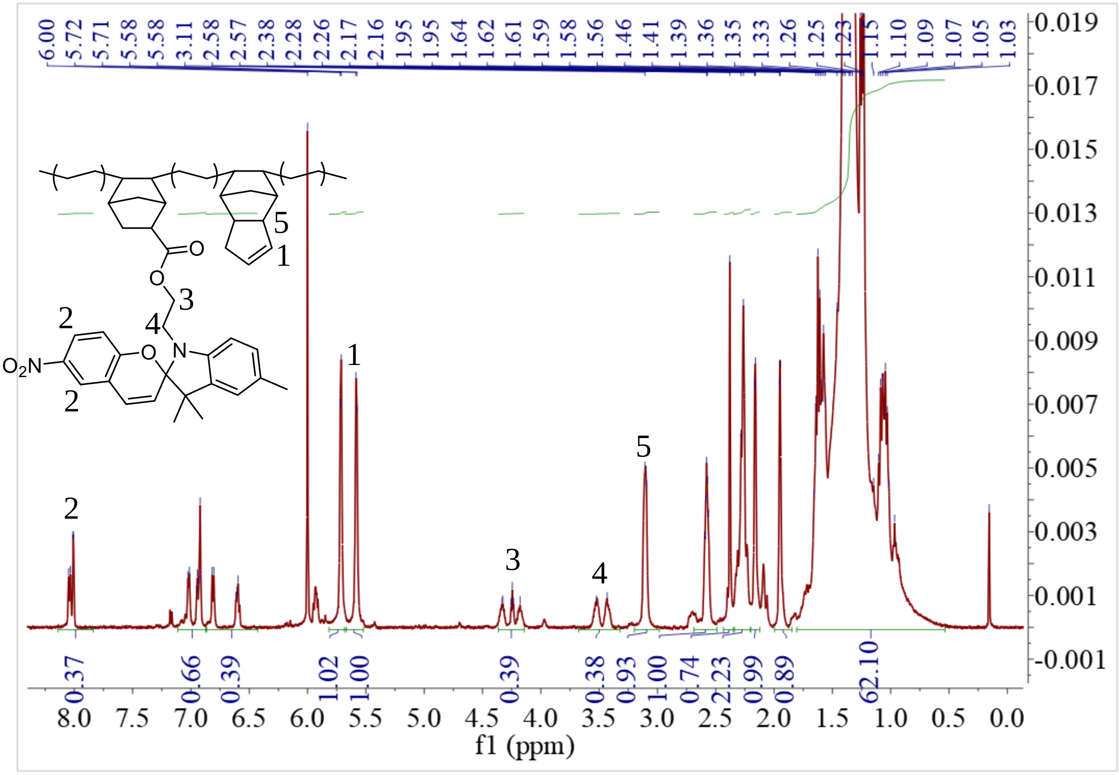

Figure S41. ^{1}H NMR spectrum of the copolymer from Table 1, Entry 11. ($C_2D_2Cl_4$, 120 °C).

$$\text{Incorp(DCPD)\%} = \frac{I_1}{I_1 + \dfrac{I_{CH_2+CH_3} - 4 \times I_1 - \dfrac{I_3 \times 8}{2I_1}}{4} + \dfrac{I_3}{2I_1}} \times 100\%$$

$$\text{Incorp(L4)\%} = \frac{\dfrac{I_3}{2I_1}}{I_1 + \dfrac{I_{CH_2+CH_3} - 4 \times I_1 - \dfrac{I_3 \times 8}{2I_1}}{4} + \dfrac{I_3}{2I_1}} \times 100\%$$

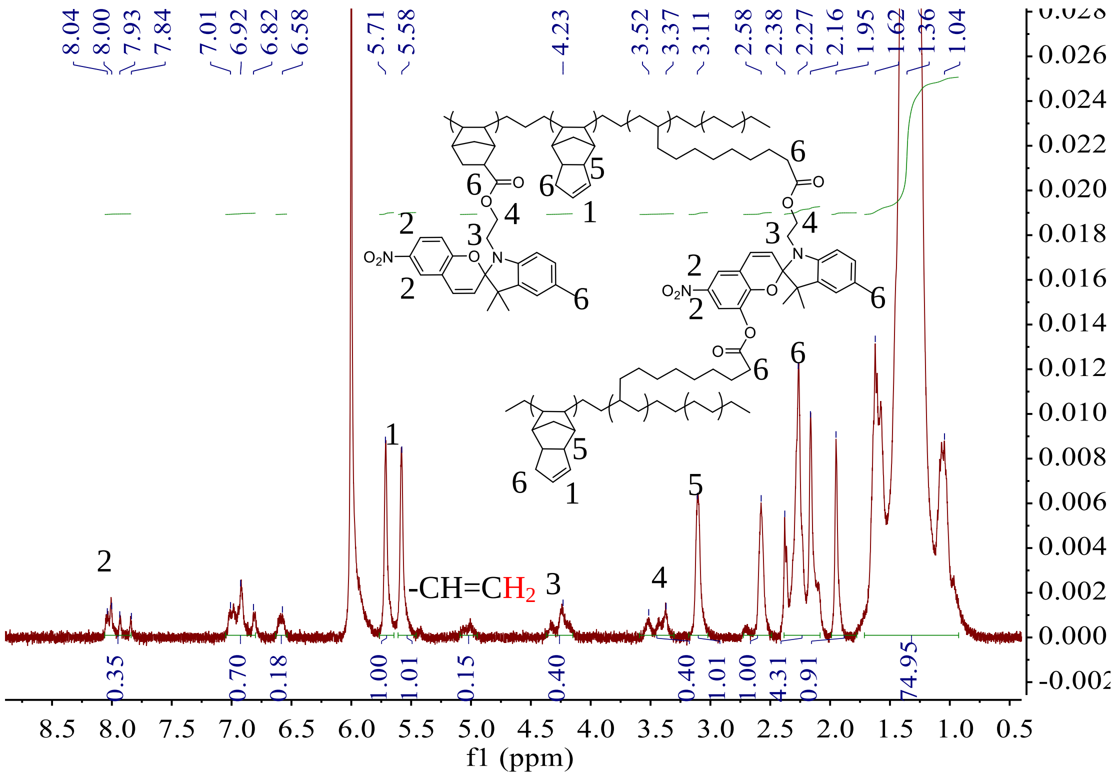

Figure S42. ^{1}H NMR spectrum of the copolymer from Table 1, Entry 12. ($C_2D_2Cl_4$, 120 °C).

The multi-peak signal corresponding to I_5 contains multiple hydrogen atoms on dicyclopentadiene and L3, so:

$$\text{One side(L3)} = \frac{I_{-CH=CH2}}{2}$$

$$\text{One side(L3)} + \text{Both side(L3)} + \text{L4} = \frac{I_3}{2}$$

$$\text{One side(L3)} \times 9 + \text{Both side(L3)} \times 7 + \text{L4} \times 4 = I_6 - I_1 \times 3$$

$$\text{Both side(L3)\%} = \frac{\text{Both side(L3)}}{\text{Both side(L3)} + \text{One side(L3)}} \times 100\% = 26.5\%$$

$$\frac{\text{L3}}{\text{L3} + \text{L4}} = \frac{\text{One side(L3)} + \text{Both side(L3)}}{\text{One side(L3)} + \text{Both side(L3)} + \text{L4}} = 0.51$$

$$\text{Incorp(DCPD)\%} = \frac{I_1}{I_1 + \frac{I_{CH2+CH3} - 4\times I_1 - \frac{I_3\times 8\times 0.49}{2I_1} - \frac{I_3\times 40\times 0.51\times 0.265}{2I_1} - \frac{I_3\times 35\times 0.51\times 0.735}{2I_1}}{4} + \frac{I_3}{2I_1}} \times 100\%$$

$$\text{Incorp(L3 + L4)\%} = \frac{\frac{I_3}{2I_1}}{I_1 + \frac{I_{CH2+CH3} - 4\times I_1 - \frac{I_3\times 8\times 0.49}{2I_1} - \frac{I_3\times 40\times 0.51\times 0.265}{2I_1} - \frac{I_3\times 35\times 0.51\times 0.735}{2I_1}}{4} + \frac{I_3}{2I_1}} \times 100\%$$

Incorp(L3)%

$$= \frac{\frac{I_3}{2I_1}\times 0.51}{I_1 + \frac{I_{CH_2+CH_3} - 4\times I_1 - \frac{I_3\times 8\times 0.49}{2I_1} - \frac{I_3\times 40\times 0.51\times 0.265}{2I_1} - \frac{I_3\times 35\times 0.51\times 0.735}{2I_1}}{4} + \frac{I_3}{2I_1}} \times 100\%$$

Incorp(L4)%

$$= \frac{\frac{I_3}{2I_1}\times 0.49}{I_1 + \frac{I_{CH_2+CH_3} - 4\times I_1 - \frac{I_3\times 8\times 0.49}{2I_1} - \frac{I_3\times 40\times 0.51\times 0.265}{2I_1} - \frac{I_3\times 35\times 0.51\times 0.735}{2I_1}}{4} + \frac{I_3}{2I_1}} \times 100\%$$

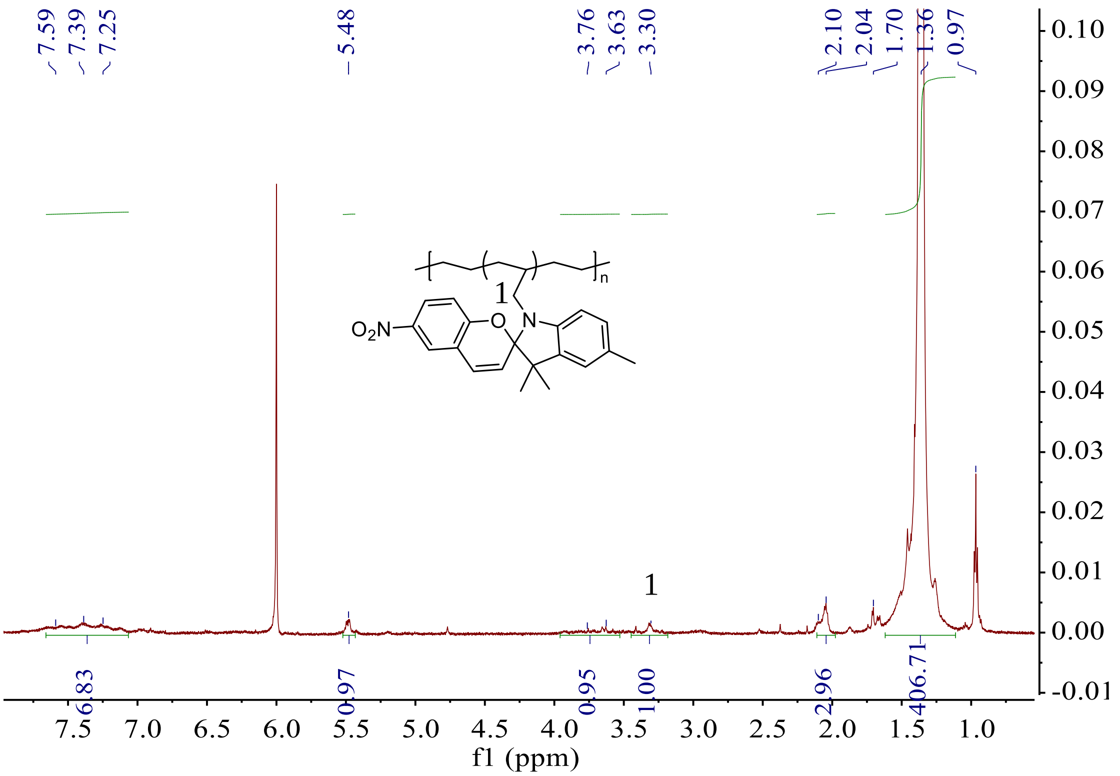

Figure S43. ^{1}H NMR spectrum of the copolymer from Table 1, Entry 1. ($C_2D_2Cl_4$, 120 ^oC).

$$\mathrm{Incorp(L1)\%} = \frac{I_1}{I_1 + \frac{I_{CH_2+CH_3} - I_1 \times 9}{4}} \times 100\%$$

11. DSC of copolymer

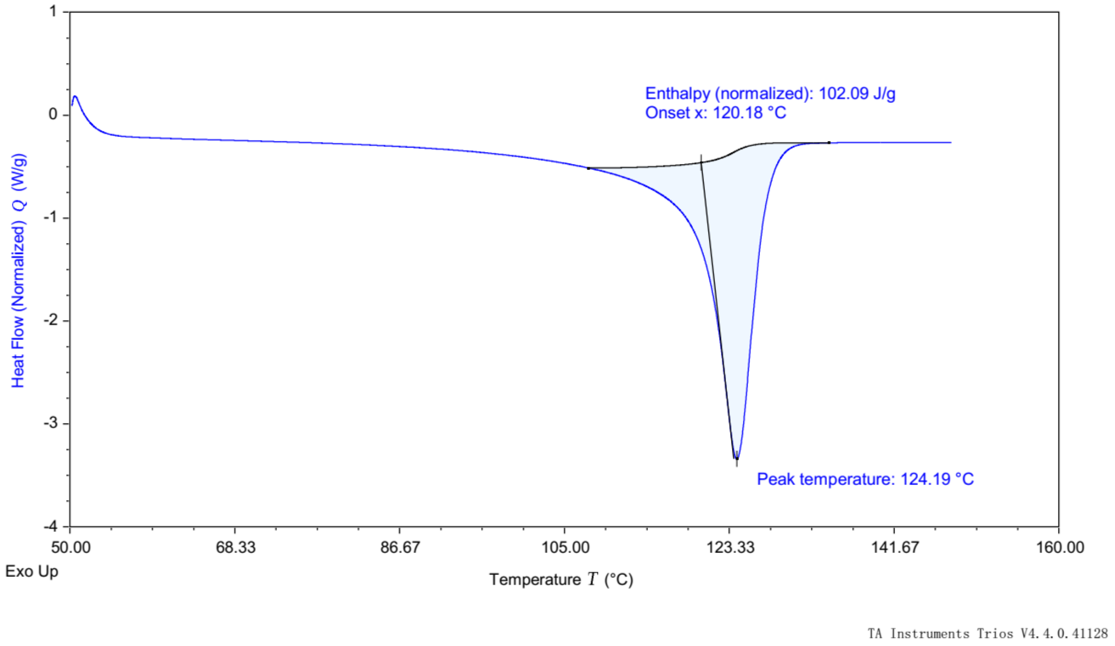

Figure S44. DSC of the copolymer from Table 1, Entry 1.

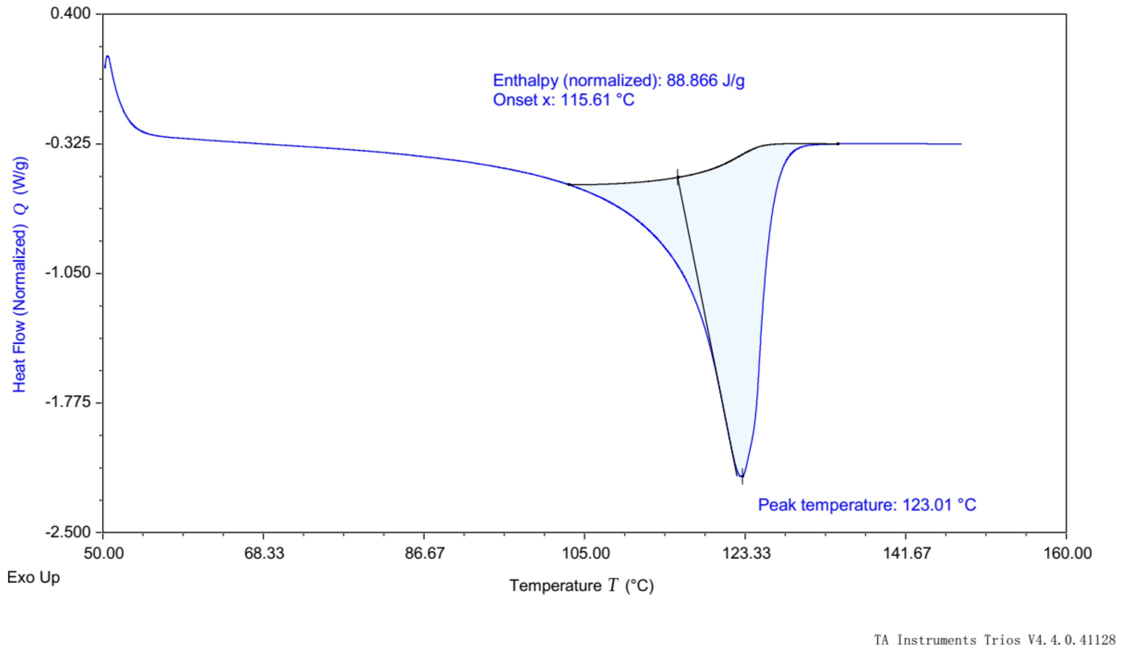

Figure S45. DSC of the copolymer from Table 1, Entry 2.

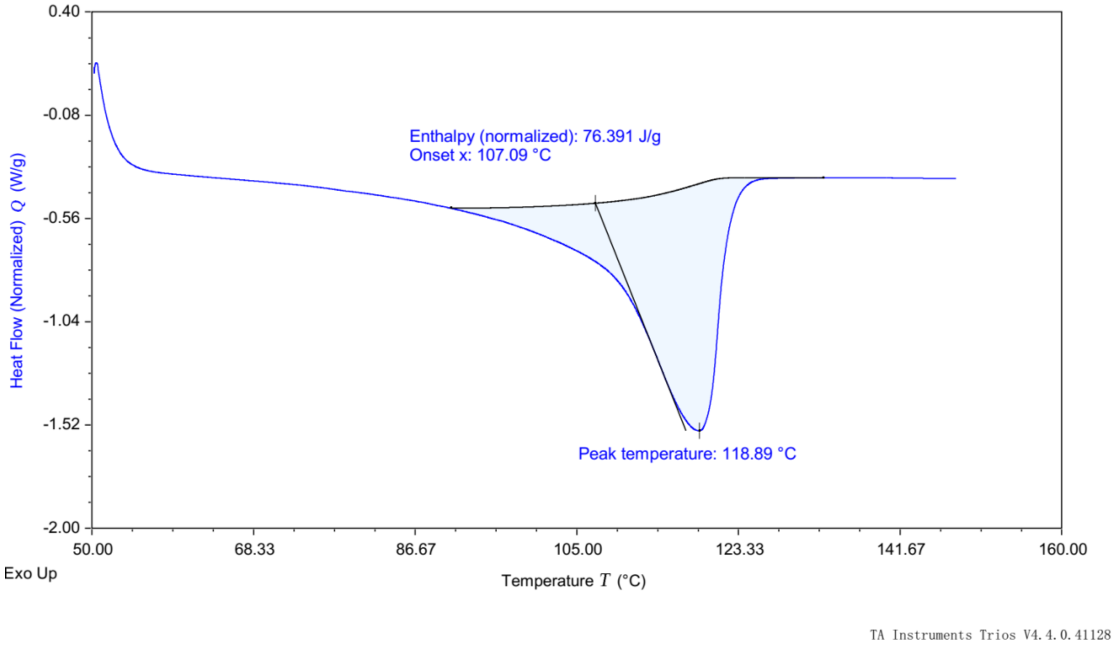

Figure S46. DSC of the copolymer from Table 1, Entry 3.

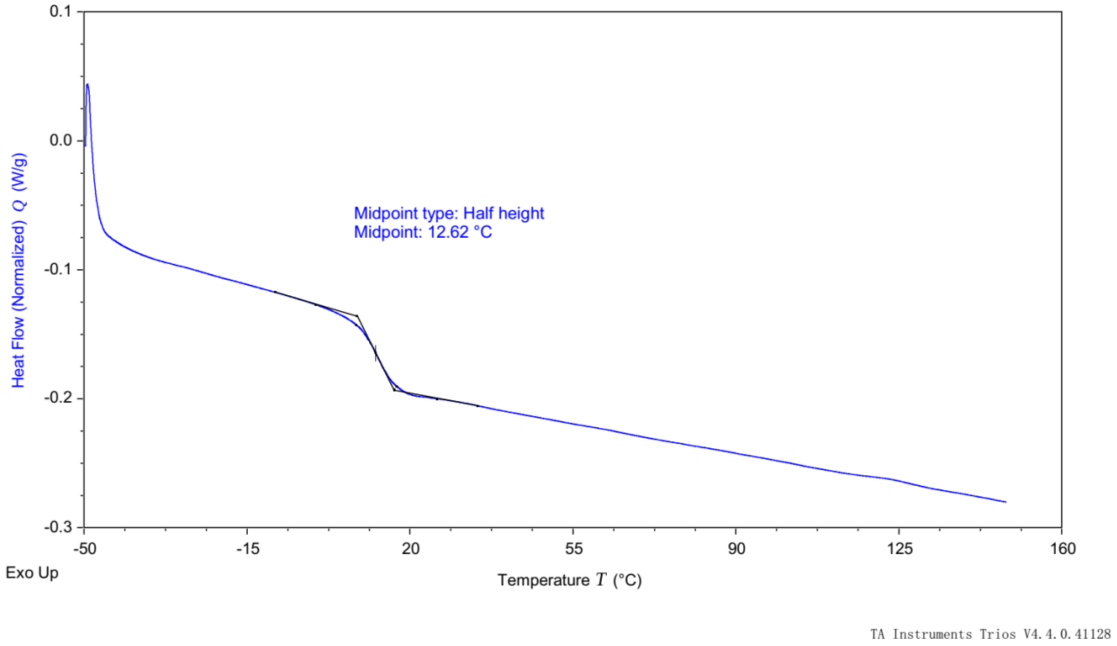

Figure S47. DSC of the copolymer from Table 1, Entry 4.

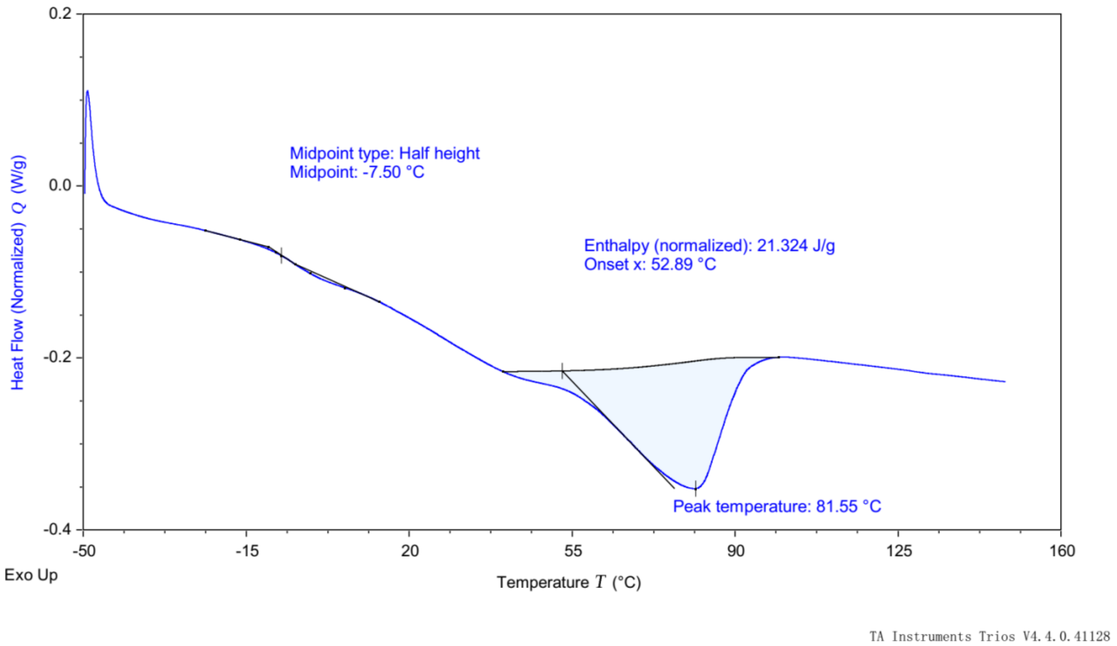

Figure S48. DSC of the copolymer from Table 1, Entry 5.

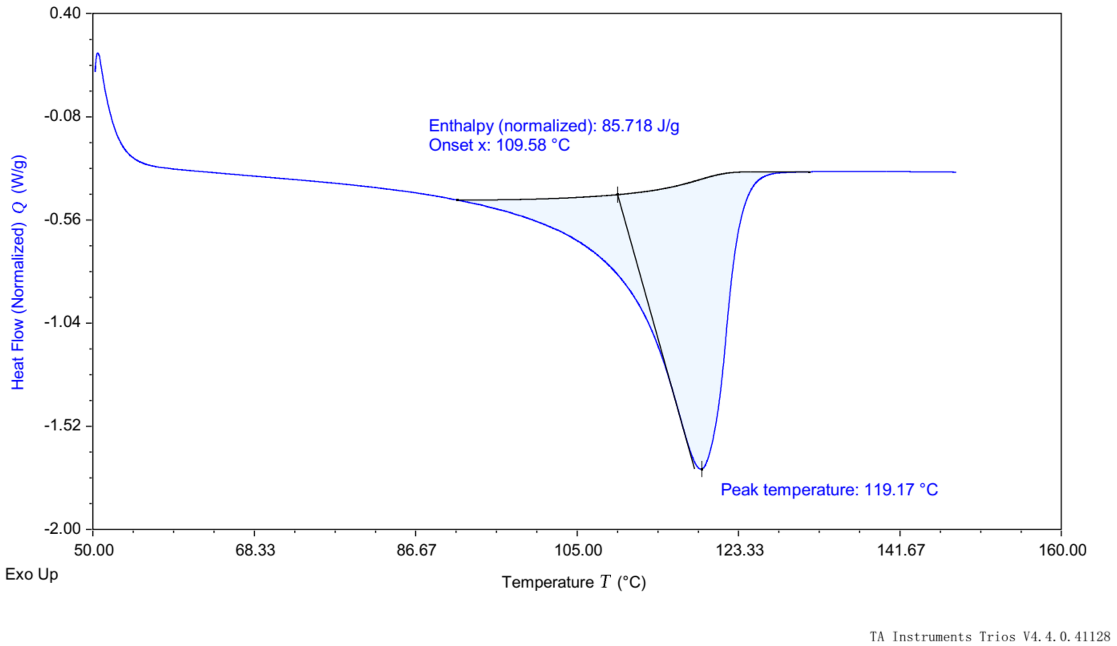

Figure S49. DSC of the copolymer from Table 1, Entry 6.

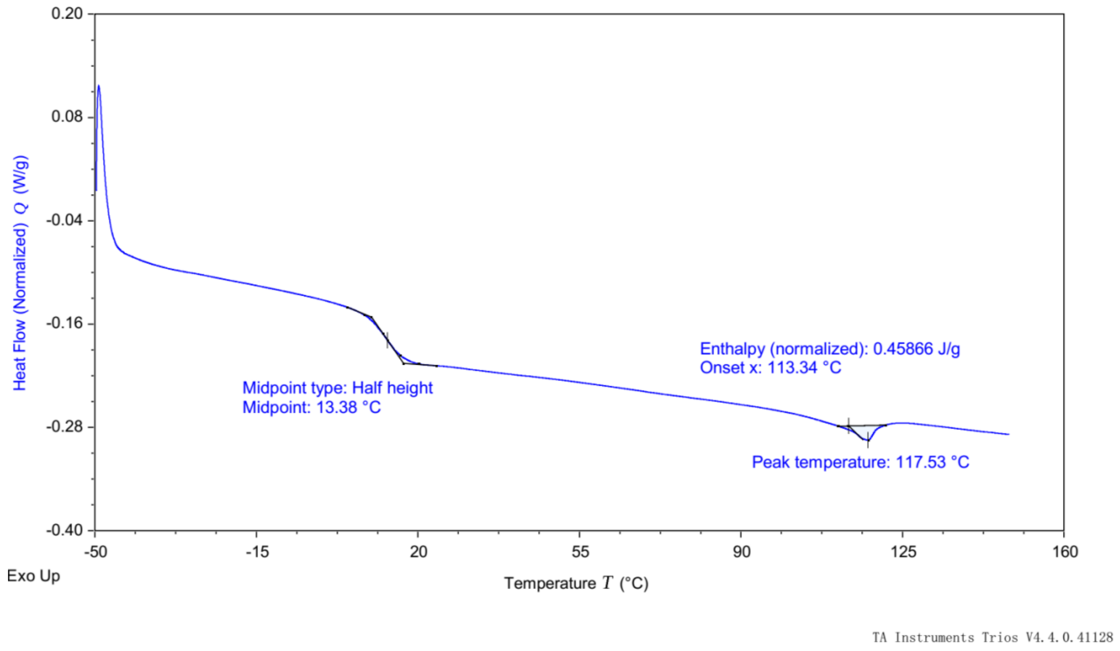

Figure S50. DSC of the copolymer from Table 1, Entry 7.

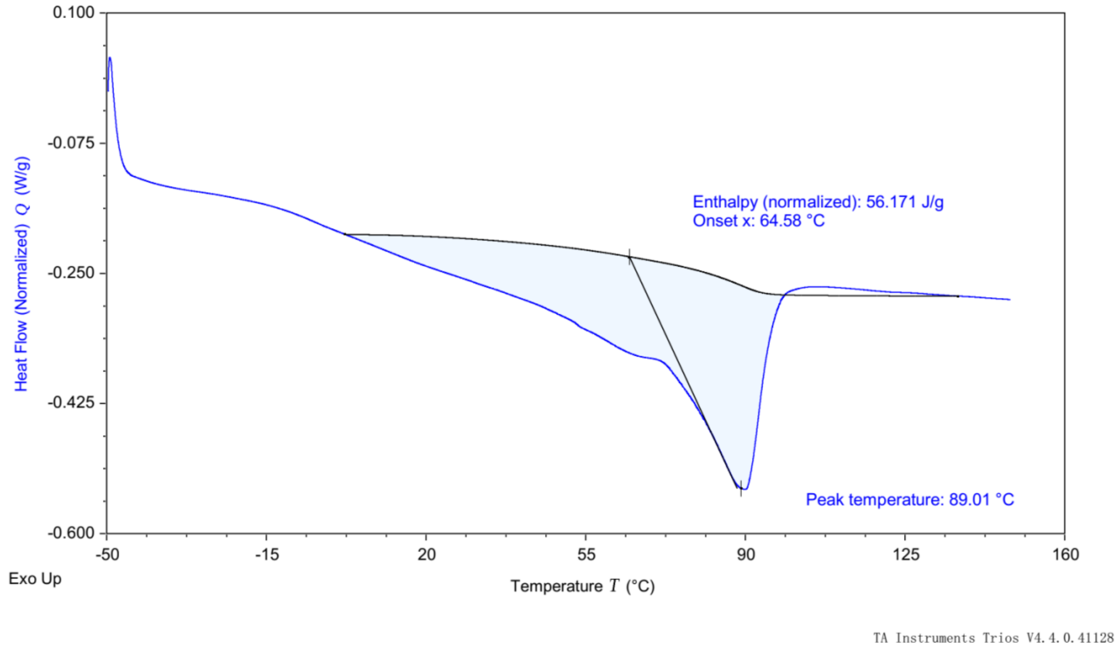

Figure S51. DSC of the copolymer from Table 1, Entry 8.

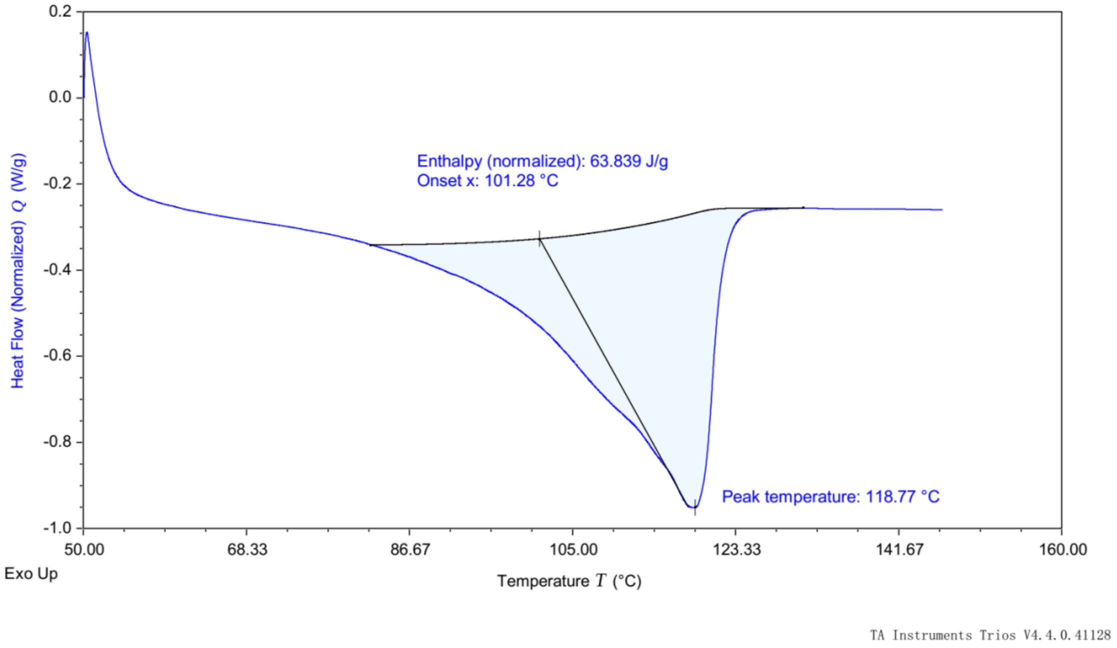

Figure S52. DSC of the copolymer from Table 1, Entry 9.

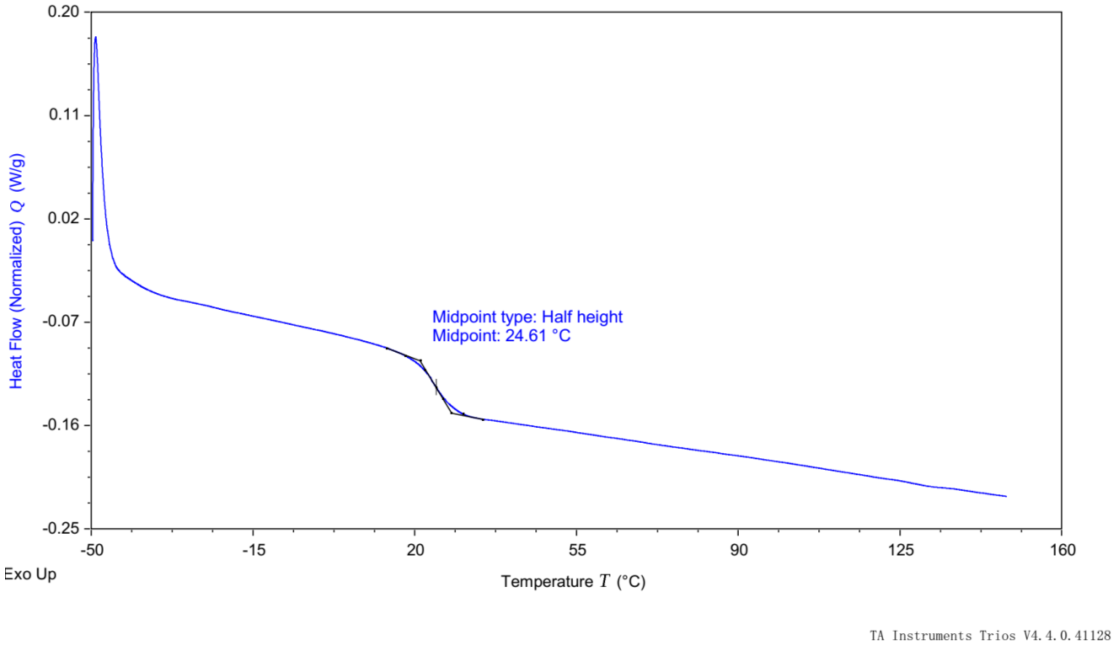

Figure S53. DSC of the copolymer from Table 1, Entry 10.

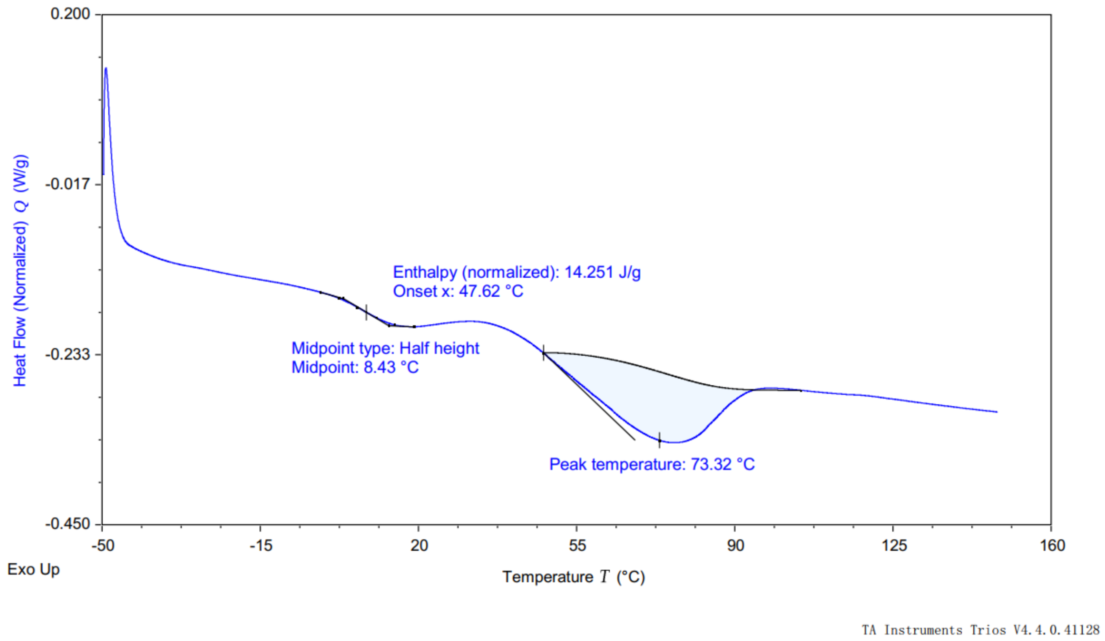

Figure S54. DSC of the copolymer from Table 1, Entry 11.

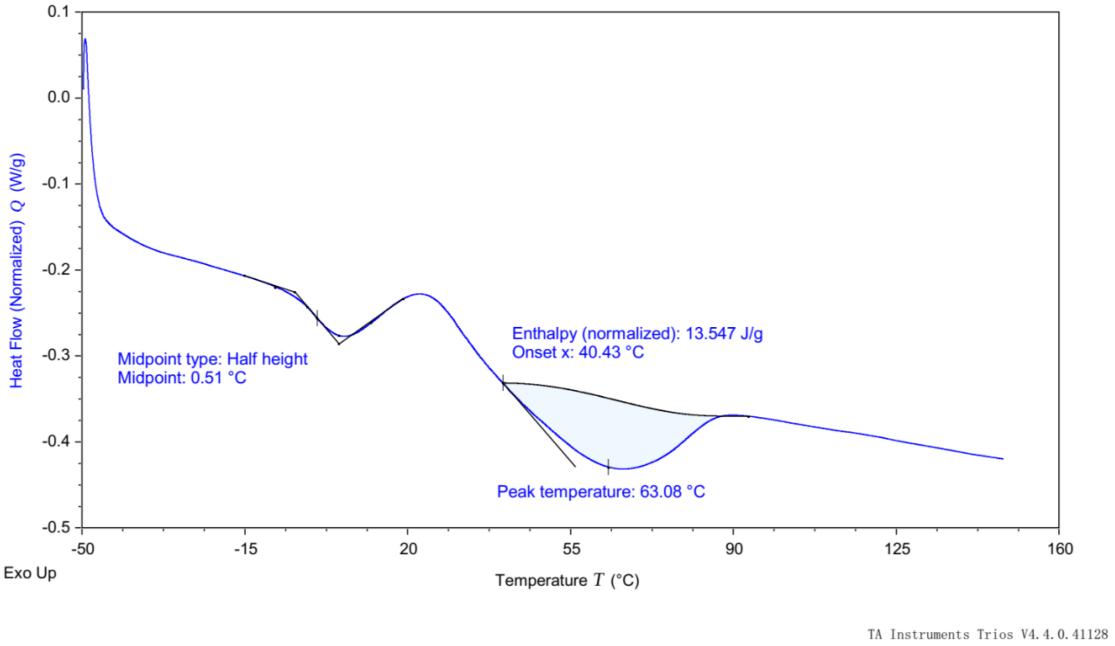

Figure S55. DSC of the copolymer from Table 1, Entry 12.

12. GPC of copolymer

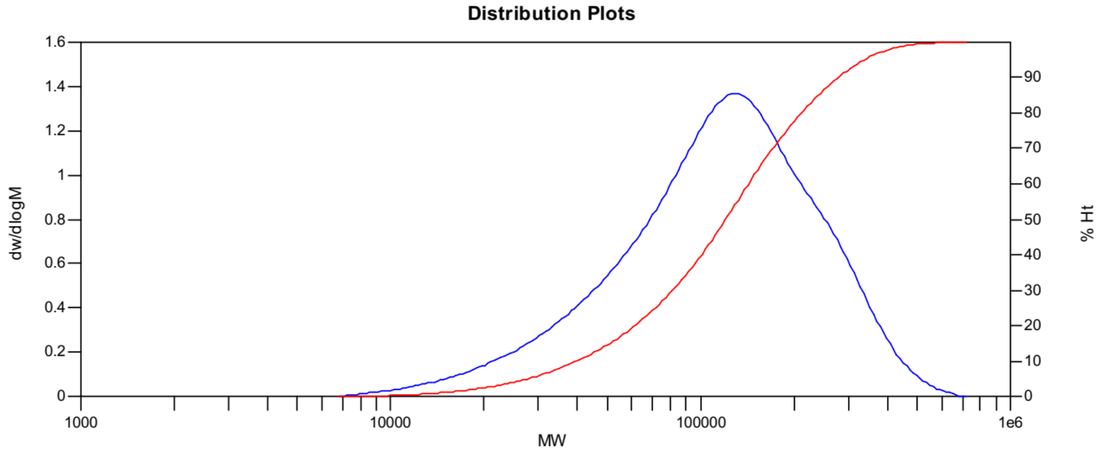

MW Averages

Peak No	Mp	Mn	Mw	Mz	Mz+1	Mv	PD
1	128913	79296	140219	206332	270500	130726	1.7683

Processed Peaks

Peak No	Name	Start RT (mins)	Max RT (mins)	End RT (mins)	Pk Height (mV)	% Height	Area (mV.secs)	% Area
1		12.63	13.80	15.87	-20.1083	0	1382.8	100

Figure S56. GPC of the copolymer from Table 1, Entry 1.

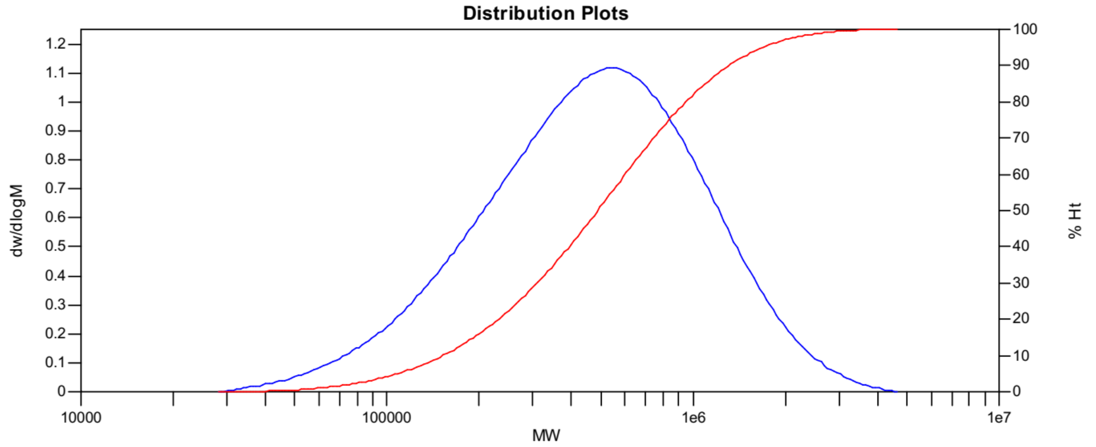

MW Averages

Peak No	Mp	Mn	Mw	Mz	Mz+1	Mv	PD
1	535923	318783	630262	1067030	1567132	574646	1.97709

Processed Peaks

Peak No	Name	Start RT (mins)	Max RT (mins)	End RT (mins)	Pk Height (mV)	% Height	Area (mV.secs)	% Area
1		11.35	12.83	14.85	-17.1103	0	1435.18	100

Figure S57. GPC of the copolymer from Table 1, Entry 2.

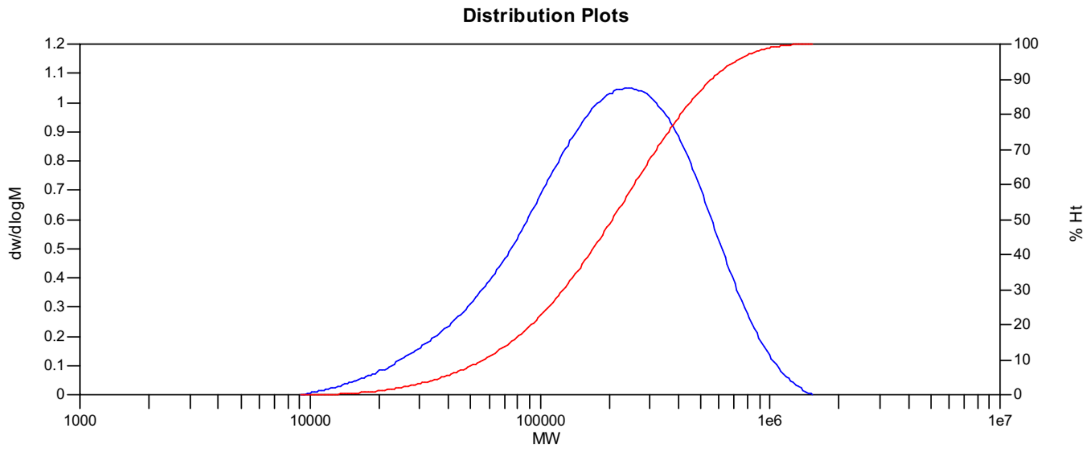

MW Averages

Peak No	Mp	Mn	Mw	Mz	Mz+1	Mv	PD
1	243956	120790	261614	433979	600972	237988	2.16586

Processed Peaks

Peak No	Name	Start RT (mins)	Max RT (mins)	End RT (mins)	Pk Height (mV)	% Height	Area (mV.secs)	% Area
1		12.12	13.37	15.65	-14.1339	0	1260.53	100

Figure S58. GPC of the copolymer from Table 1, Entry 3.

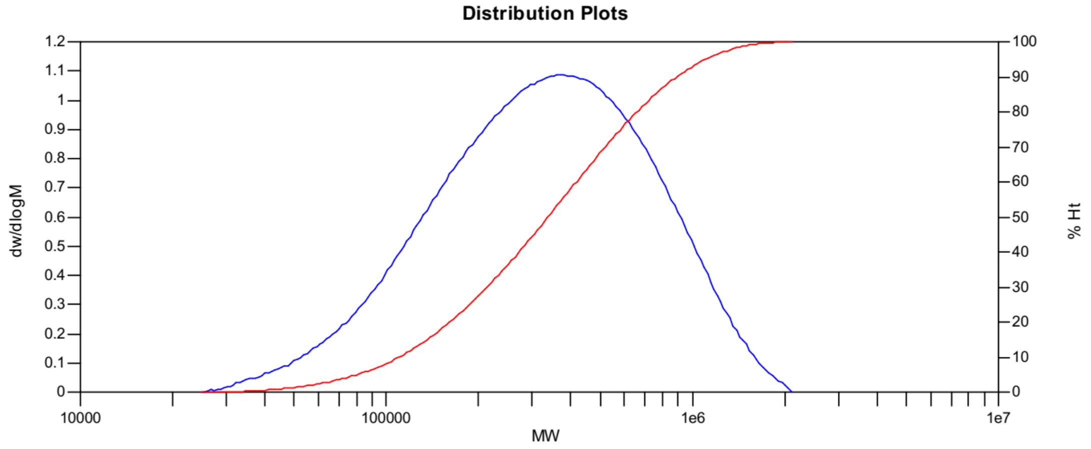

MW Averages

Peak No	Mp	Mn	Mw	Mz	Mz+1	Mv	PD
1	361573	227944	425281	673806	911676	391134	1.86573

Processed Peaks

Peak No	Name	Start RT (mins)	Max RT (mins)	End RT (mins)	Pk Height (mV)	% Height	Area (mV.secs)	% Area
1		11.90	13.10	14.93	-8.02273	0	690.75	100

Figure S59. GPC of the copolymer from Table 1, Entry 4.

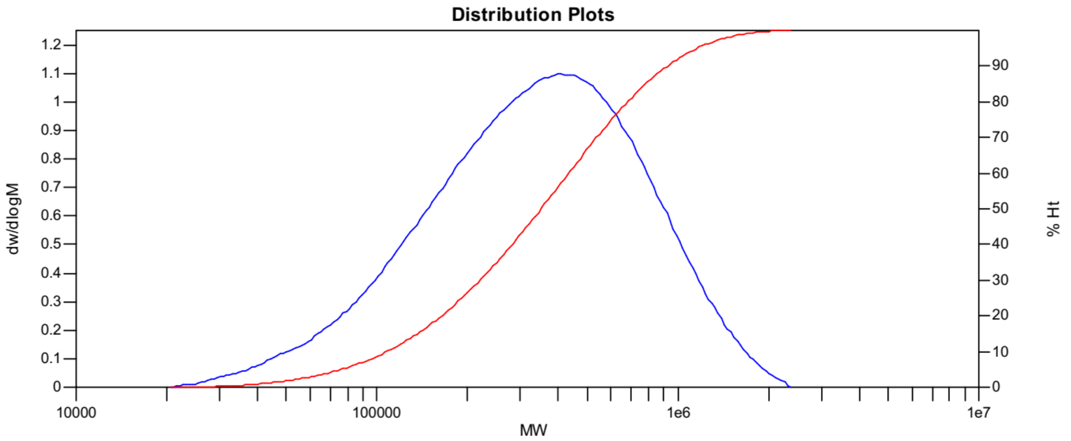

MW Averages

Peak No	Mp	Mn	Mw	Mz	Mz+1	Mv	PD
1	408896	225425	441658	712193	978756	404757	1.95922

Processed Peaks

Peak No	Name	Start RT (mins)	Max RT (mins)	End RT (mins)	Pk Height (mV)	% Height	Area (mV.secs)	% Area
1		11.82	13.02	15.07	-9.6929	0	826.272	100

Figure S60. GPC of the copolymer from Table 1, Entry 5.

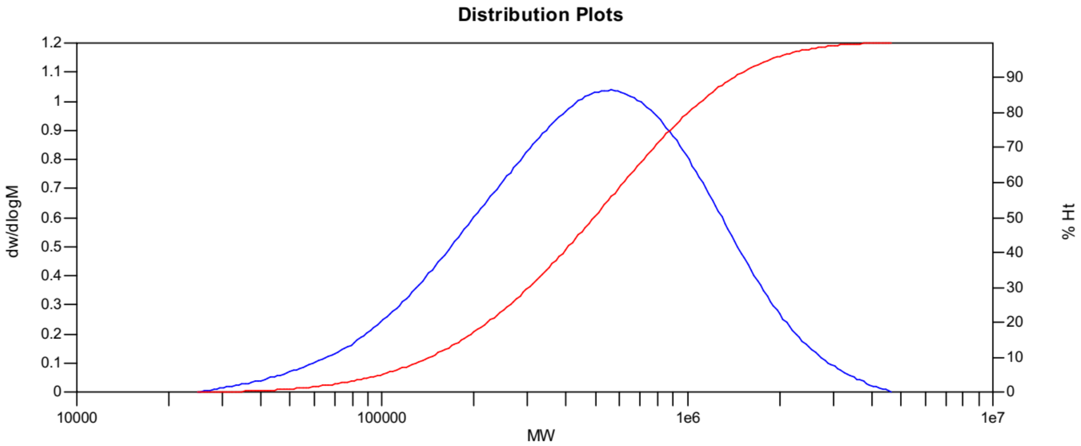

MW Averages

Peak No	Mp	Mn	Mw	Mz	Mz+1	Mv	PD
1	562931	305359	659056	1167054	1731294	594939	2.1583

Processed Peaks

Peak No	Name	Start RT (mins)	Max RT (mins)	End RT (mins)	Pk Height (mV)	% Height	Area (mV.secs)	% Area
1		11.35	12.80	14.93	-16.7254	0	1509.37	100

Figure S61. GPC of the copolymer from Table 1, Entry 6.

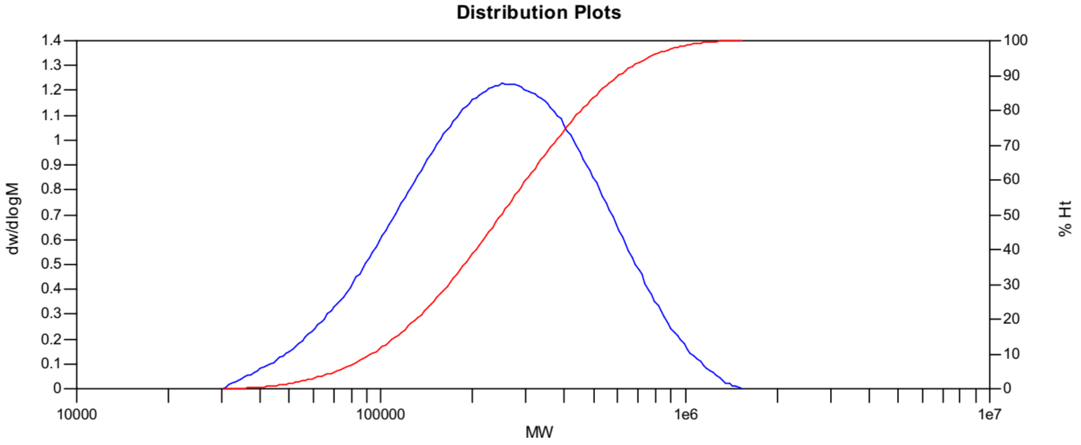

MW Averages

Peak No	Mp	Mn	Mw	Mz	Mz+1	Mv	PD
1	250027	184303	303358	456396	614319	282584	1.64597

Processed Peaks

Peak No	Name	Start RT (mins)	Max RT (mins)	End RT (mins)	Pk Height (mV)	% Height	Area (mV.secs)	% Area
1		12.12	13.35	14.80	-7.15339	0	545.728	100

Figure S62. GPC of the copolymer from Table 1, Entry 7.

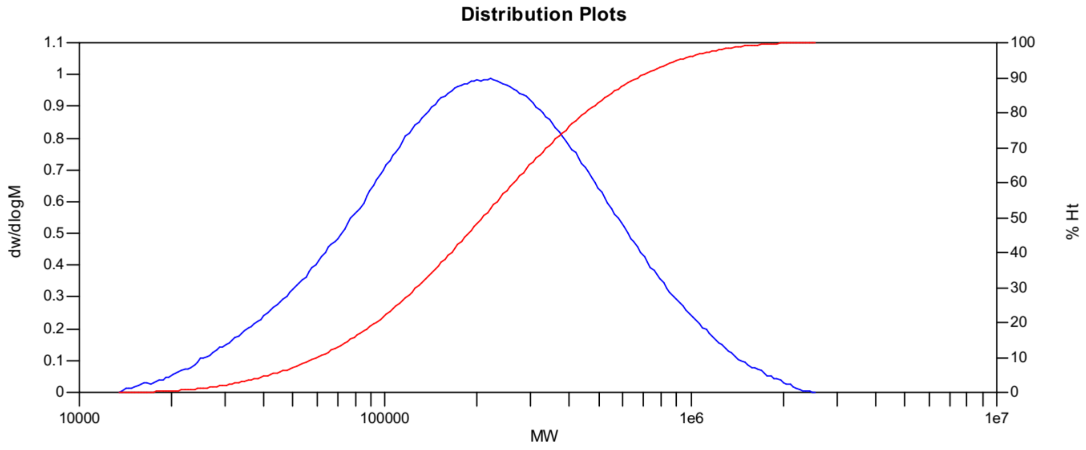

MW Averages

Peak No	Mp	Mn	Mw	Mz	Mz+1	Mv	PD
1	221118	130775	299509	587382	924688	265972	2.29026

Processed Peaks

Peak No	Name	Start RT (mins)	Max RT (mins)	End RT (mins)	Pk Height (mV)	% Height	Area (mV.secs)	% Area
1		11.77	13.43	15.37	-6.76051	0	641.1	100

Figure S63. GPC of the copolymer from Table 1, Entry 8.

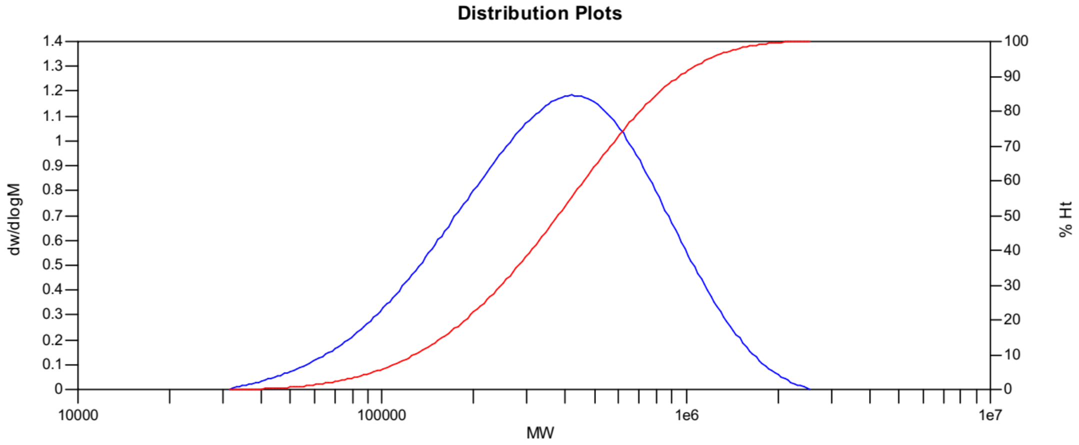

MW Averages

Peak No	Mp	Mn	Mw	Mz	Mz+1	Mv	PD
1	419079	261648	467330	728959	999190	431930	1.7861

Processed Peaks

Peak No	Name	Start RT (mins)	Max RT (mins)	End RT (mins)	Pk Height (mV)	% Height	Area (mV.secs)	% Area
1		11.77	13.00	14.77	-14.0757	0	1113.4	100

Figure S64. GPC of the copolymer from Table 1, Entry 9.

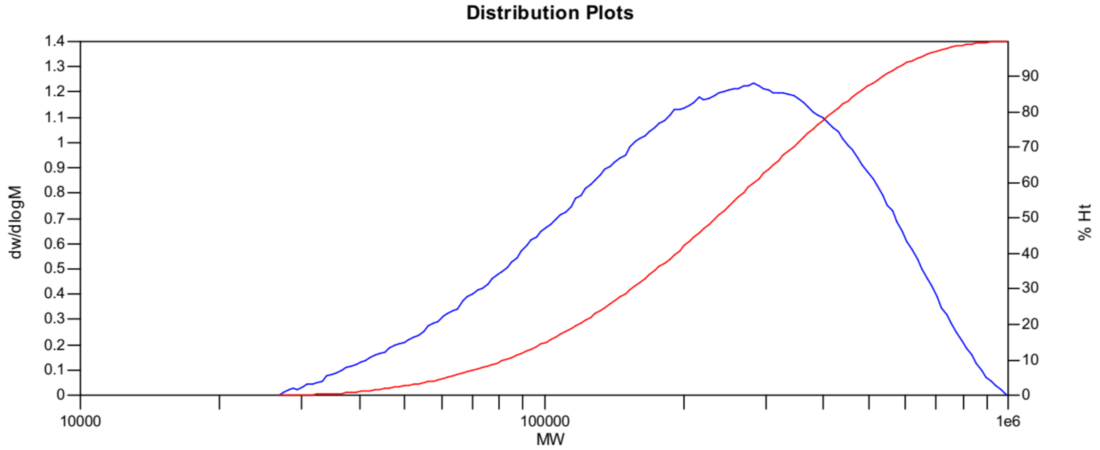

MW Averages

Peak No	Mp	Mn	Mw	Mz	Mz+1	Mv	PD
1	282732	166488	271782	385883	483753	254810	1.63244

Processed Peaks

Peak No	Name	Start RT (mins)	Max RT (mins)	End RT (mins)	Pk Height (mV)	% Height	Area (mV.secs)	% Area
1		12.42	13.27	14.88	-3.79636	0	288.134	100

Figure S65. GPC of the copolymer from Table 1, Entry 10.

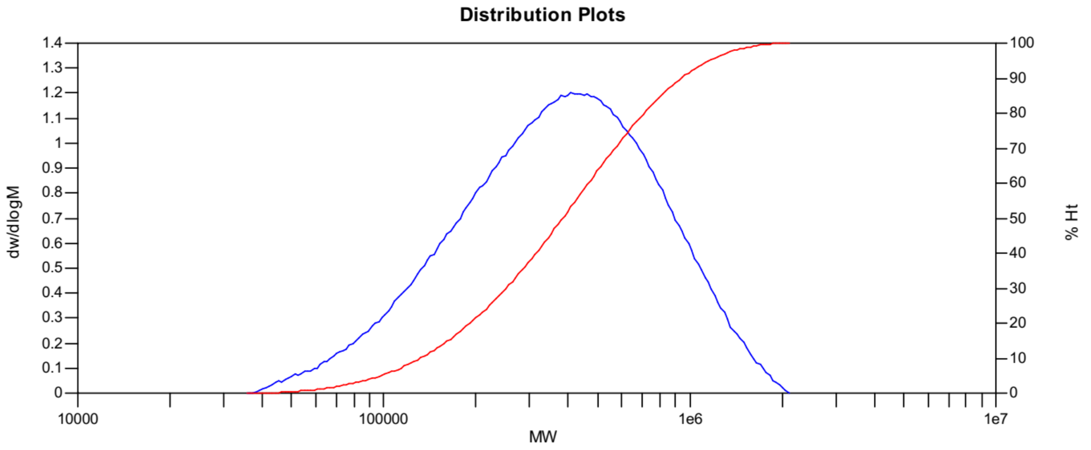

MW Averages

Peak No	Mp	Mn	Mw	Mz	Mz+1	Mv	PD
1	408896	269354	465801	702402	929102	432657	1.72933

Processed Peaks

Peak No	Name	Start RT (mins)	Max RT (mins)	End RT (mins)	Pk Height (mV)	% Height	Area (mV.secs)	% Area
1		11.90	13.02	14.68	-4.98574	0	388.416	100

Figure S66. GPC of the copolymer from Table 1, Entry 11.

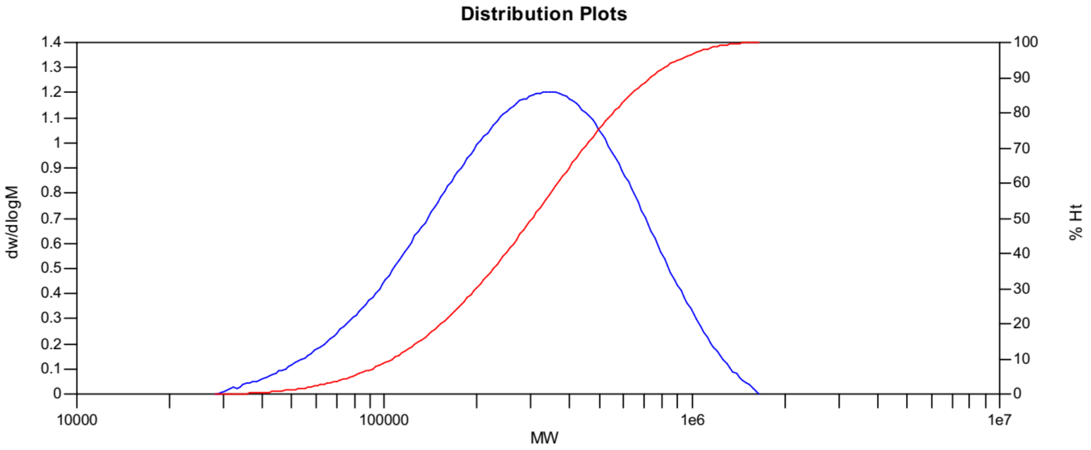

MW Averages

Peak No	Mp	Mn	Mw	Mz	Mz+1	Mv	PD
1	344214	213635	364974	547068	723203	339491	1.7084

Processed Peaks

Peak No	Name	Start RT (mins)	Max RT (mins)	End RT (mins)	Pk Height (mV)	% Height	Area (mV.secs)	% Area
1		12.07	13.13	14.85	-7.93537	0	616.549	100

Figure S67. GPC of the copolymer from Table 1, Entry 12.

13. References

[1] D. A. Davis, A. Hamilton, J. Yang, L. D. Cremar, D. V. Gough, S. L. Potisek, M. T. Ong, P. V. Braun, T. J. Martínez, S. R. White, J. S. Moore, N. R. Sottos, *Nature* **2009**, *459*, 68.

[2] G. R. Gossweiler, G. B. Hewage, G. Soriano, Q. Wang, G. Welshofer, X. Zhao, S. L. Craig, *ACS Macro Lett.* **2014**, *3*, 216.

[3] D. S. Achilleos, T. A. Hatton, M. Vamvakaki, *J. Am. Chem. Soc.* **2012**, *134*, 5726.

[4] P. Perrotin, J. J. McCahill, G. Wu, S. L. Scott, *Chem. Commun.* **2011**, *47*, 6948.
